# Molecular and Biochemical Analysis of Duplicated Cytosolic CuZn Superoxide Dismutases of Rice and *in silico* Analysis in Plants

**DOI:** 10.3389/fpls.2022.864330

**Published:** 2022-05-30

**Authors:** Ravi Prakash Sanyal, Vishal Prashar, Narendra Jawali, Ramanjulu Sunkar, Hari Sharan Misra, Ajay Saini

**Affiliations:** ^1^Molecular Biology Division, Bhabha Atomic Research Centre, Mumbai, India; ^2^Homi Bhabha National Institute, Mumbai, India; ^3^Radiation Biology and Health Sciences Division, Bhabha Atomic Research Centre, Mumbai, India; ^4^Centre for Natural Biological Resources and Community Development, Bengaluru, India; ^5^Department of Biochemistry and Molecular Biology, Oklahoma State University, Stillwater, OK, United States

**Keywords:** block duplication, cytosolic CuZn superoxide dismutase, heteromeric interaction, homology modeling, *in silico* analysis, *Oryza sativa*, oxidative stress, thermostability

## Abstract

Superoxide dismutases (SODs, EC 1.15.1.1) are ubiquitous antioxidant metalloenzymes important for oxidative stress tolerance and cellular redox environment. Multiple factors have contributed toward the origin and diversity of SOD isoforms among different organisms. In plants, the genome duplication events, responsible for the generation of multiple gene copies/gene families, have also contributed toward the SOD diversity. However, the importance of such molecular events on the characteristics of SODs has not been studied well. This study investigated the effects of divergence on important characteristics of two block-duplicated rice cytosolic CuZn SODs (*OsCSD1*, *OsCSD4*), along with *in silico* assessment of similar events in other plants. The analysis revealed heterogeneity in gene length, regulatory regions, untranslated regions (UTRs), and coding regions of two *OsCSDs*. An inconsistency in the database-predicted *OsCSD1* gene structure was also identified and validated experimentally. Transcript analysis showed differences in the basal levels and stress responsiveness of *OsCSD1* and *OsCSD4*, and indicated the presence of two transcription start sites in the *OsCSD1*. At the amino acid level, the two OsCSDs showed differences at 18 sites; however, both exist as a homodimer, displaying typical CuZn SOD characteristics, and enhancing the oxidative stress tolerance of *Escherichia coli* cells. However, OsCSD4 showed higher specific activity as well as stability. The comparison of the two OsCSDs with reported thermostable CSDs from other plants identified regions likely to be associated with stability, while the homology modeling and superposition highlighted structural differences. The two OsCSDs displayed heteromeric interaction capability and forms an enzymatically active heterodimer (OsCSD1:OsCSD4) on co-expression, which may have significance as both are cytosolic. *In silico* analysis of 74 plant genomes revealed the prevalence of block duplications for multiple *CSD* copies (mostly cytosolic). The divergence and clustering analysis of CSDs suggested the possibility of an ancestral duplication event in monocots. Conserved SOD features indicating retention of SOD function among CSD duplicates were evident in few monocots and dicots. In most other species, the CSD copies lacked critical features and may not harbor SOD function; however, other feature-associated functions or novel functions might be present. These aspects of divergent *CSD* copies encoding co-localized CSDs may have implications in plant SOD functions in the cytosol and other organelles.

## Introduction

Abiotic stress conditions affect all aspects of plant physiology, resulting in a negative impact on growth, development, and productivity (Boyer, 1982). Plants respond to various stress conditions by activation of appropriate adaptive responses, which involves re-programming of cellular machinery at multiple levels, diverse as well as overlapping pathways, and complex molecular crosstalks (Kreps et al., 2002; Rizhsky et al., 2004; Mittler, 2006; Zhu, 2016; Haak et al., 2017). The abiotic stresses cause damage to cellular integrity in multiple ways, leading to metabolic dysfunction (Zhu, 2016; Pandey et al., 2017). Most stress conditions lead to elevated levels of reactive oxygen species (ROS) and reactive nitrogen species (RNS), causing “oxidative and nitrosative stress” to the cell (Hausladen and Stamler, 1999; Mittler, 2002; Huang et al., 2019). The ROS/RNS are crucial for diverse physiological processes, signaling, and stress responses (Gill and Tuteja, 2010; Del Río, 2015; Dietz et al., 2016). The physiologically relevant ROS/RNS levels require an intricate balance between generation and scavenging, where both non-enzymatic and enzymatic antioxidants (including SODs) are involved in cellular ROS/RNS homeostasis and protection from oxidative damage (Gill and Tuteja, 2010; Mittler, 2017; Huang et al., 2019).

The superoxide radical (O_2_^⋅–^, half-life: 2–4 μs), generated in several oxidative metabolic reactions, is moderately reactive and relatively less damaging (Halliwell, 2006; Das and Roychoudhury, 2014). However, it participates in the formation of other ROS/RNS, including highly reactive hydroxyl radical (^•^OH) and peroxynitrite (ONOO^–^) that damages important components (DNA, proteins, lipids) vital to cellular functions (Wang et al., 2018; Staszek and Gniazdowska, 2020). Enzymatic scavenging of O_2_^⋅–^ (dismutation into O_2_ and H_2_O_2_) is catalyzed by SODs, important antioxidant metalloenzymes, present among diverse life forms (McCord and Fridovich, 1969; Alscher et al., 2002). The SODs directly scavenge O_2_^⋅–^ and also reduce levels of other ROS/RNS (^•^OH, ONOO^–^, etc.), and therefore considered the first line of defense against ROS-mediated damage (McCord and Fridovich, 1969; Alscher et al., 2002; Das and Roychoudhury, 2014). Based on the catalytic metal co-factors, plant SODs are divided into three groups, namely Fe, Mn, and CuZn SODs (Fink and Scandalios, 2002; Miller, 2012; Del Río et al., 2018). The differential localization of Fe SOD (chloroplast), Mn SOD (mitochondria), and the most abundant CuZn SOD (peroxisome, chloroplast, and cytosol) (Alscher et al., 2002; Gill et al., 2015) is critical for localized scavenging of the membrane-impermeable O_2_^⋅–^ (Takahashi and Asada, 1983). In addition, it is equally crucial for inter-organellar communication and regulation of gradient and flux of H_2_O_2_, an important signaling molecule (Jankù et al., 2019).

CuZn SODs (hereafter referred to as CSDs) exist as homodimers, homotetramers, and monomers (Fridovich, 1986; Sundaram et al., 2009; Mishra et al., 2014), with each subunit containing two cofactors, Cu^2+^ for catalysis and Zn^2+^ for dimerization and stability (Pelmenschikov and Siegbahn, 2005; Li H. T. et al., 2010). Eukaryotic CSDs, with high β-sheet and low α-helical content, exhibit conservation of structurally/functionally important sites (Khanna-Chopra and Sabarinath, 2004). However, variations in non-critical regions often affect important characteristics (e.g., specific activity, stability, subunit interaction, pH range), as reported in certain plant CSDs (Lin et al., 2002; Kumar et al., 2012, 2014, 2016b; Mahanty et al., 2012; Mishra et al., 2014; Tuteja et al., 2015; Sanyal et al., 2018; Fesharaki-Esfahani et al., 2021). The response of plant CSDs to different conditions have been extensively studied (Gupta et al., 1993; Gill et al., 2015; Negi et al., 2015; Guan et al., 2017; Lightfoot et al., 2017; Del Río et al., 2018; Sanyal et al., 2018; Tyagi et al., 2019), where few other candidates/mechanisms are also involved in modulation of their expression/function, viz. miR398 (Sunkar et al., 2006; Jagadeeswaran et al., 2009; Li Y. F. et al., 2010), post-translational modifications (PTMs, Yamakura and Kawasaki, 2010), natural antisense transcripts of miR398 genes (Li et al., 2020), and alternative splicing (Srivastava et al., 2009; Sagasti et al., 2014; Saini et al., 2021).

Superoxide dismutases are believed to have originated due to oxygenic photosynthesis-mediated transition of the atmosphere (reducing to oxidizing), which affected the metal co-factor availability leading to the evolution of three isoforms (Blankenship, 2010; Inupakutika et al., 2016; Case, 2017). The Fe and Mn SODs are the most primitive (Miller, 2012) while CSDs have evolved relatively late that also involved structural changes to accommodate the Cu cofactor (Bannister et al., 1991; Inupakutika et al., 2016; Dreyer and Schippers, 2019). During evolution, genomic mechanisms also contributed toward the diversity of genes among plants (Qiao et al., 2019). Most plants have undergone multiple duplication events (whole genome duplication, inter/intra chromosome block duplication, and tandem duplication), leading to multiple gene copies including many stress-responsive genes (Flagel and Wendel, 2009; Panchy et al., 2016). In such a scenario, some copies can accumulate variations and may diverge toward an altered regulation/function and gain of novel function, while others may become pseudogenes or undergo deletion (Barker et al., 2012; Panchy et al., 2016; Qiao et al., 2019).

Plants generally contain multiple CSDs, with each type for a specific compartment, including a single CSD for cytosol. However, many plants, such as *Sorghum bicolor*, *Gossypium raimondii*, *Gossypium arboretum*, and *Medicago truncatula*, harbor multiple CSD copies as a consequence of duplication events (Filiz and Tombuloğlu, 2015; Wang et al., 2016; Song et al., 2018); however, in general, the information at the protein level is limited. Rice genome contains a total of four loci coding for CSDs (LOC_Os03g22810, LOC_Os03g11960, LOC_Os07g46990, LOC_LOC_Os08g44770), of which two (LOC_Os03g22810 and LOC_Os07g46990) coding for cytosolic CSDs OsCSD1 and OsCSD4 (Sanyal et al., 2018)^1^ are located in the inter-chromosomal block duplicated segments involving chromosomes 3 and 7 (Thiel et al., 2009; Yadav et al., 2019). Previous studies have analyzed certain characteristics of different rice CSDs, viz. gene/regulatory regions, transcript and/or protein levels, and response to stress conditions including oxidative stress (Kanematsu and Asada, 1989; Sakamoto et al., 1995; Kaminaka et al., 1997, 1999; Pan et al., 1999; Guan et al., 2017; Sanyal et al., 2018; Saini et al., 2021). Studies have also shown that the rice cytosolic CSDs show differential responses to abscisic acid, H_2_O_2_, drought, and salinity at the transcript level (Kaminaka et al., 1999; Morita et al., 2011). The duplicated gene copies often evolve toward differential regulation/function or may acquire novel functions (Flagel and Wendel, 2009; Barker et al., 2012). The impact of duplication on the functioning of rice cytosolic CSDs has not been fully addressed and would be worth investigating.

This study investigated the regulatory, functional, and structural characteristics of duplicated rice cytosolic CSDs, OsCSD1, and OsCSD4. We observed that sequence divergence could affect organization, basal expression, stress response, and alternative splicing pattern of these two genes. Although, both OsCSDs retained typical SOD characteristics, protected *Escherichia coli* from oxidative stress, and showed heteromeric subunit interaction, the specific activity and stability of OsCSD4 were found to be higher than OsCSD1. Further, *in silico* analysis showed that the duplication events lead to CSD copies in 50–64% of plants across monocots and dicots. Less divergent CSD duplicates are likely to retain SOD function (as in rice), but higher divergence suggests altered function, limited/partial function, or altogether new function, with/without SOD activity, among different plants. Overall, this study highlighted the importance of block duplications in generating CSD diversity in plants, which may be important for cytosolic SOD function by yet uncharacterized mechanisms, under different conditions.

## Materials and Methods

### Plant Material, Growth Conditions, and Stress Treatment

Rice genotype NSICRc106 (salt tolerant, obtained from International Rice Research Institute, IRRI Philippines) was used in this study. Seeds were surface sterilized with 0.1% mercuric chloride (HgCl_2_), rinsed in de-ionized water, and kept for germination. Rice seedlings were transferred to 1X Hoagland media (HiMedia, India) and grown hydroponically in a plant growth chamber (MLR-351H, Sanyo, Japan) under 14-h light and 10-h dark period using following settings: 150 μmol m^–2^s^–1^ light intensity (photon flux density), temperature: 28 ± 1°C/26 ± 1°C (light period/dark period), and 65% humidity. The growth media were replaced every third day. Six-day-old seedlings were subjected to the following stress conditions: salinity (150 mM sodium chloride, NaCl), drought (15% polyethylene glycol, PEG), and oxidative stress (10 μM methyl viologen, MV). Samples (shoot tissue) were collected at 24 and 48 h time points from control and stressed seedlings. To study the effect of light, control tissue samples were also collected at different time points during the day cycle (0, 0.25, 0.5, 1, 3, 5, 7, and 11 h). Tissue samples were immediately frozen in the liquid nitrogen and stored at −70°C until further use. The chemicals and reagents used (if not specifically mentioned) were from Sigma-Aldrich (United States), and the molecular biology protocols were followed as per Sambrook and Russell (2001).

## Isolation of Total RNA, cDNA Synthesis, and Reverse Transcription-qPCR Analysis

Total RNA was isolated using TRIzol (Invitrogen, United States) and treated with DNase I (Roche Diagnostics, Germany). DNase I was subsequently heat inactivated and the RNA preparation was assessed for quality and quantity. The total RNA (10 μg) was reverse transcribed using a mixture of anchored oligo(dT)_35_ and random nonamers (dN_9_, New England Biolabs, United States) and SuperScript II reverse transcriptase (Invitrogen, United States), as per the protocol recommended by the manufacturer. First-strand cDNA preparation was quantified spectrophotometrically (on UV-1800, Shimadzu, Japan) and used for both reverse transcription-qPCR (RT-qPCR) analysis and full-length cDNA amplification. The RT-qPCR was carried out as described earlier (Sanyal et al., 2018) using oligonucleotide primers specific to *OsCSD1* and *OsCSD4*, designed using the exon organization information available at Rice Genome Annotation Project website (Kawahara et al., 2013; Supplementary Table 1).^1^^,2^ In brief, RT-qPCR assays were carried out on the LightCycler LC480 II real-time PCR (Roche Diagnostics, Germany) using the SYBR Green Jumpstart *Taq* Ready mix (Sigma-Aldrich, United States), with the following settings: 94°C (2 min), 45 cycles of 94°C (10 s), 60°C (15 s), and 68°C (20 s). The amplified products were subjected to melting curve analysis to assess the specificity. The data was analyzed as per Schmittgen and Livak (2008) using *Actin-2* (LOC_Os10g36650) and *GAPDH* (LOC_Os08g03290, additional reference gene for validation) (Supplementary Table 1). The analysis was carried out using three biological replicates (8–10 seedlings were pooled) and three technical replicates for each set of samples. The statistical analysis of control and treated samples was carried out by the Student’s *t*-test and the differences were considered significant only when the *P* < 0.05.

### Polymerase Chain Reaction Amplification for Exon Analysis, Full-Length cDNA Isolation, and Cloning

The oligonucleotide primers were designed using gene organization information available on RGAP website for PCR amplifications of *OsCSD1* and *OsCSD4* full-length cDNAs (FL-cDNAs), overlapping *OsCSD1* DNA fragments, exon analysis, and validation of transcription status of *OsCSD1* exon3 (Supplementary Table 1). The PCR reactions were carried out using *Pwo* DNA polymerase (Roche Diagnostics, Germany) on a Mastercycler Gradient PCR system (Eppendorf, Germany) using the following conditions: initial denaturation at 94°C (5 min), 35 cycles of 94°C for 45 s, 60°C for 45 s, and 72°C for (20 s-5 min, as per amplicon size), final extension at 72°C (5 min). FL-cDNAs were purified, double digested with *Nde*I and *Eco*RI, ligated to plasmid pET28a(+) (digested with the same restriction enzymes), and transformed into *E. coli* (DH5α) cells. The transformants were selected on LB-Agar plates containing kanamycin (25 μg ml^–1^) and screened for the presence of cDNA inserts by colony PCR and restriction analysis of plasmids. The cDNAs were sequenced and submitted to the GenBank (*OsCSD1*: MW091043; *OsCSD4*: MW091044), and hereafter the corresponding recombinant plasmids are referred to as pET28a-OsCSD1 and pET28a-OsCSD4).

### *In silico* Sequence Analysis and Homology Modeling

Several online/offline *in silico* tools were used for sequence analysis of *CSD* isoforms from rice and other monocot and dicot species. The sequences of genomic loci of *OsCSD1* and *OsCSD4* were retrieved from two databases, Rice Genome Annotation Project (RGAP)^1^ and Rice Annotation Project (RAP-DB),^3^ and used for comparison of gene structure and exon-intron organization. The sequences of monocot and dicot CuZn SOD isoforms (similar to rice CSDs) were identified by the Basic Local Alignment Search Tool^4^, retrieved from GenBank (NCBI)^5^ and PLAZA (version 4.5) web server^6^ and used for analysis (Supplementary Table 2). The theoretical molecular weight (Mw) and isoelectric point (pI) were estimated using the “Compute pI/Mw tool”,^7^ while the surface charge was estimated using the Protein Calculator (v3.4) tool.^8^ Secondary structure elements were predicted using the Chou and Fasman (1974) Secondary Structure Prediction server (CFSSP).^9^ Domain organization was analyzed using the Conserved Domain Database web resource at NCBI (CDD).^10^ The promoter analysis was carried out at the Plant Promoter Analysis Navigator tool (PlantPan3.0),^11^ *cis*-acting regulatory elements were searched at the PlantCARE database,^12^ and the presence of Transcription start sites (TSS) was analyzed using the TSSPlant tool.^13^ Transcript levels of the two CSDs were analyzed at the RGAP^1^ and Rice Expression database (RED).^14^ The comparative analysis of block and tandem duplication events among genomes of monocots and dicots was carried out at PLAZA (version 4.5) web resource (see text footnote 6) and represented using the Circleplot tool available at the server. The multiple sequence alignments were performed by ClustalX (Thompson et al., 1997) using the default parameters, followed by alignment editing by the BioEdit software (Hall, 1999). The analysis of sequence divergence and genetic relationships was carried out by the neighbor-joining method (Saitou and Nei, 1987) in the Molecular Evolutionary Genetic Analysis software (MEGA version X, Kumar et al., 2018), and the statistical analysis was performed by the bootstrap method (Felsenstein, 1985).

The homology models of the rice CSDs were generated using the SWISS-MODEL (Biasini et al., 2014) workspace.^15^ The dimeric rice CSD sequences were used as a target to generate the homology model against the *Solanum lycopersicum* CuZn SOD crystal structure template (PDB ID: 3PU7). The template showed high-sequence identity with the query sequence and the structure, refined to 1.8 Å resolution, included both Cu and Zn co-factors. The structural superposition of the two homology models was carried out using the molecular modeling software O (Jones et al., 1991), and figures were rendered using the computer program PyMOL (DeLano, 2002).

### Overexpression and Purification of Recombinant Proteins

The recombinant plasmids (pET28a-OsCSD1 and pET28a-OsCSD4) were transformed into *E. coli* SHuffle T7 Express strain (New England Biolabs, United States) for heterologous overexpression, as described previously (Sanyal et al., 2018). Briefly, *E. coli* SHuffle T7 Express cells containing recombinant pET28a-OsCSD1 or pE28a-OsCSD4 plasmid were grown overnight at 30°C in LB medium containing kanamycin (25 μg ml^–1^, LB-Kan^+^). The cultures were diluted (1:100) in the fresh LB-Kan medium containing 0.2 mM CuCl_2_ and ZnCl_2_. The cells in the mid-log phase were induced with IPTG (0.25 mM, temperature: 25°C, time: 16 h), harvested by centrifugation (4,500 × g, 10 min), and re-suspended in the re-suspension buffer (Tris-HCl: 20 mM; NaCl: 200 mM; PMSF: 1 mM, pH: 8.0). The cells were subjected to sonication (amplitude: 35%, ON time: 2 s, OFF time: 2 s, temperature: 4°C, processing time: 15 min, repeated twice). The cell lysate was centrifuged (4,500 × g, 10 min) to remove cell debris, followed by another centrifugation step (13,680 × g, 20 min, 4°C). The supernatant and pellet fractions were resolved on 15% SDS-PAGE and stained with Coomassie Brilliant Blue R-250 (Sigma-Aldrich, United States). The recombinant proteins were purified from the supernatant fraction by affinity chromatography using cOmplete™ His-Tag purification resin (Roche Diagnostics, Germany) and analyzed on 15% SDS-PAGE. Using the 12-kDa MWCO dialysis tube (Sigma-Aldrich, United States), the affinity-purified proteins were dialyzed against the dialysis buffer (Tris-HCl: 20 mM; NaCl: 200 mM; pH: 8.0), concentrated using Vivaspin 3-kDa MWCO column (GE Healthcare, United States), further purified by gel-filtration chromatography (detailed below), and quantitated by BCA method (Smith et al., 1985) using bovine serum albumin (BSA) as standard.

### Determination of Native and Subunit Molecular Weight

The native molecular weight of the proteins was determined by gel-filtration chromatography on Superdex™ 75 10/300 GL or Superdex 200 increase 10/300 GL (GE Healthcare, United States) columns, pre-equilibrated with Tris-HCl buffer (20 mM, NaCl: 200 mM, pH: 8.0). The columns were pre-calibrated with the following reference proteins: aldolase (158 kDa), bovine serum albumin (66.5 kDa), chicken egg albumin (45 kDa), carbonic anhydrase (29 kDa), and cytochrome C (12.4 kDa). For determination of the subunit’s molecular weight, the proteins were resolved on 15% SDS-PAGE along with molecular weight standards (New England Biolabs, United States).

### Biophysical Studies

The secondary structure of recombinant proteins was evaluated by the circular dichroism (CD) analysis on an M-500 CD spectrometer (BioLogic Science Instruments, France) at 25°C. In brief, the protein sample (5.0 μM) in potassium phosphate buffer (10 mM, pH: 8.0) containing 10 mM KCl was used. The CD spectra were recorded between wavelengths 190 and 260 nm using the following settings, path length: 0.5 cm, Acq duration: 0.2 s, and band width: 2.0 nm. Measurements from three scans were averaged and corrected for the sample buffer. The protein concentration estimated by the BCA method was used to determine the molar ellipticity.

The differential scanning fluorimetry (DSF) analysis was used to determine the melting temperature (Tm) of the recombinant proteins on the LightCycler LC480 II real time PCR (Roche Diagnostics, Germany) with wavelength settings of 465 nm (excitation) and 580 nm (emission). In brief, 0.5 mg ml^–1^ of purified protein in 50 mM Tris-HCl buffer (pH: 8.0) was mixed with 5X SYPRO orange (S5692, Sigma-Aldrich, United States) and fluorescence data were continuously recorded using the following settings: initial incubation at 25°C for 10 min, a gradual increase of temperature to 95°C (ramp rate: 0.04°C s^–1^) with 10 data point acquisitions °C^–1^. Tm calling function was used to generate the first derivative curve and to estimate the melting temperature (Tm) of the proteins.

### Biochemical Characterization

The superoxide dismutase (SOD) activity of recombinant rice CSDs was determined by a multi-well plate-based nitroblue-tetrazolium (NBT) reduction method (Ewing and Janero, 1995). Briefly, increasing concentration (0.0–2.0 μg ml^–1^) of purified protein was added to the reaction mixture (volume: 200 μl) containing NADH (78 μM), NBT (50 μM), and EDTA (0.1 mM), in Tris-HCl buffer (50 mM, pH 8.0). The reaction was initiated by the addition of PMS (1.65 μM), and the absorbance (A_560nm_) was recorded for 5 min on a multi-well plate reader (Infinite M200, Tecan, United Kingdom) and used to estimate the SOD activity. 1U SOD activity is defined as the protein amount required for inhibition of NBT reduction by 50%. The kinetic parameters (Vmax and Km) were estimated spectrophotometrically at different NADH concentrations (0–30 μM) in the SOD assay. Vmax and Km values were estimated from the activity vs. substrate concentration plot using non-linear regression analysis option in the GraphPad 7.0 software. An amount of purified protein equivalent to 1U SOD activity was used for the biochemical assays, as described previously (Sanyal et al., 2018). The effect of pH on the activity and stability of CSDs was investigated using the following buffers: acetate buffer (4.0–5.0), phosphate buffer (pH: 6.0–8.0), Tris-HCl buffer (pH range: 7.0–9.0), and bicarbonate (pH: 9.0–10.8). For determination of the pH optima, SOD activity was determined at different pH by the NBT reduction method (O_2_**^⋅–^** generation rate, A_560nm_ ∼0.05–0.06 min^–1^ kept constant). For the assessment of the effect of pH on stability, the proteins were pre-incubated at different pH and aliquots were taken out at the different time points (1–24 h) and assayed for SOD activity by NBT reduction assay at pH 8.0. For determining the effect of temperature, the proteins in 50 mM Tris-HCl buffer (pH: 8.0) were pre-incubated at different temperatures (range: 25–80°C) for 1 h. Afterward, the samples were immediately chilled on ice and then assayed for SOD activity.

The sensitivity of recombinant CSDs toward SOD inhibitors, diethyldithiocarbamate (DDC), sodium azide (NaN_3_), and hydrogen peroxide (H_2_O_2_) was analyzed. The proteins were pre-incubated with increasing concentrations of DDC (0–2.0 mM), NaN_3_ (0–10.0 mM), and H_2_O_2_, (0–5.0 mM), and after 1 h, SOD activity was determined by NBT reduction assay. In-gel SOD activity assay was performed as per Chen et al. (2001) protocol. The purified rice CSDs were analyzed by electrophoresis on a 10% native PAGE gel. The gel was incubated in “solution” A (50 mM sodium phosphate buffer, pH 8.0; 28 μM riboflavin and 28 mM TEMED), and after 30 min, “solution B” (50 mM sodium phosphate buffer, pH 8.0 and 1 mM NBT) was added. The gel was exposed to light (20 min) for development and photographed on a gel-documentation system (Syngene, United Kingdom). Bicarbonate-dependent peroxidase activity of rice CSDs was determined by monitoring the oxidation of dichlorodihydrofluorescein (DCFH) to dichlorofluorescein (DCF) as per Zhang et al. (2002), with minor modifications. Briefly, the assay was carried out at 25°C in potassium phosphate buffer (100 mM, pH: 7.4) containing diethylenetriaminepentaacetic acid (DTPA, 100 μM), DCFH (50 μM), sodium bicarbonate (NaHCO_3_, 25 mM), and increasing concentration (0–250 nM) of proteins. The reaction was initiated by the addition of 0.3 mM H_2_O_2_, and the fluorescence signal of DCF (excitation: 480 nm; emission: 524 nm) was monitored for 5 min on a fluorescence multi-well plate reader (Infinite M200, Tecan, United Kingdom).

### Analysis of Oxidative Stress Tolerance of *Escherichia coli* Cells Overexpressing Rice CSDs

The effect of methyl viologen (MV)-induced oxidative stress on the growth of *E. coli* SHuffle T7 Express cells harboring pET28a (vector) and recombinant plasmid (pET28a-OsCSD1 or pET28a-OsCSD4) was analyzed as per the method described previously (Sanyal et al., 2018), with minor modifications. Briefly, a single isolated colony was inoculated in LB media containing kanamycin (25 μg ml^–1^), grown at 30°C for 16 h, on an orbital shaker. The cultures were diluted (1:100) in the LB-Kan^+^ media containing metal cofactors (0.2 mM each of CuCl_2_ and ZnCl_2_), grown until absorbance (A_600nm_) reached ∼0.4, and induced with 0.5 mM IPTG for 4 h. The cultures were normalized again to absorbance (A_600nm_) of ∼0.1, subjected to increasing MV concentration (0–0.500 mM) in the presence of 0.5 mM IPTG for 24 h (temperature: 25°C), and the cell growth was measured at regular intervals on a UV-1800 spectrophotometer (Shimadzu, Japan). In addition, the cultures at 24-h time point were also serially diluted in saline and analyzed by spot test (10 μl respective dilutions were spotted) on an LB-agar plate containing kanamycin (25 μg ml^–1^). The experiments were repeated three times.

### Analysis of Heterodimeric Interaction Between Subunits of OsCSD1 and OsCSD4

The interaction between the subunits of two rice CSDs was evaluated by the Bacterial Adenylate Cyclase Two-Hybrid (BACTH) System (Karimova et al., 1998). Briefly, two proteins of interest are fused in-frame with T25 and T18 fragments of *Bordetella pertussis* adenylate cyclase (CyaA). The interaction between the proteins functionally complements T25 and T18 interaction, resulting in a cyclic AMP (cAMP)-mediated characteristic phenotype (Battesti and Bouveret, 2012). The OsCSD1 and OsCSD4 cDNAs were cloned in compatible vectors (pKT25 and pUT18C) to generate recombinant plasmids pKT25-OsCSD1/OsCSD4 and pUT18C-OsCSD4/OsCSD1 using the standard genetic engineering protocols. The two plasmids were co-transformed into *E. coli* BTH101 strain and transformants were screened on LB-Agar plates containing ampicillin (100 μg ml^–1^) and kanamycin (25 μg ml^–1^). Positive clones were inoculated in 3 ml LB-Kan^+^Amp^+^ media containing 0.5 mM IPTG and kept overnight on a shaker at 30°C. A culture volume of 5 μl was spotted on LB-Kan^+^Amp^+^ plates containing X-Gal (40 μg ml^–1^) and IPTG (0.5 mM), incubated overnight at 30°C, and scored for hybrid expression and interaction analysis. The analysis was repeated three times with appropriate controls.

The OsCSD1:OsCSD4 heterodimer was isolated by co-expression of the two OsCSDs (containing different tags) followed by affinity-tag-based purification approaches. OsCSD1 and OsCSD4 FL-cDNAs were cloned into pMAL-c5x vector (contain MBP-tag and ampicillin resistance gene). Plasmids pMAL-c5x-OsCSD1/OsCSD4 and pET28a-OsCSD1 (contain His-tag and kanamycin resistance gene) were co-transformed into SHuffle T7 Express strain (New England Biolabs, United States). Co-transformed *E. coli* cells were grown in LB-Kan^+^Amp^+^ and induced under the conditions mentioned above for heterologous expression of OsCSDs. The recombinant dimeric OsCSDs containing subunits with His-tag (homodimer), MBP tag (homodimer), or both His and MBP tags (heterodimer) were separated from soluble fraction using a combination of Ni-NTA affinity, amylose affinity, and gel-filtration chromatography. The purified proteins were analyzed for subunit configuration on SDS-PAGEs and SOD activity, using conditions detailed previously.

## Results

### Duplicated Rice *OsCSD1* and *OsCSD4* Genes Showed Heterogeneity in Gene Structure

**FIGURE 1:**
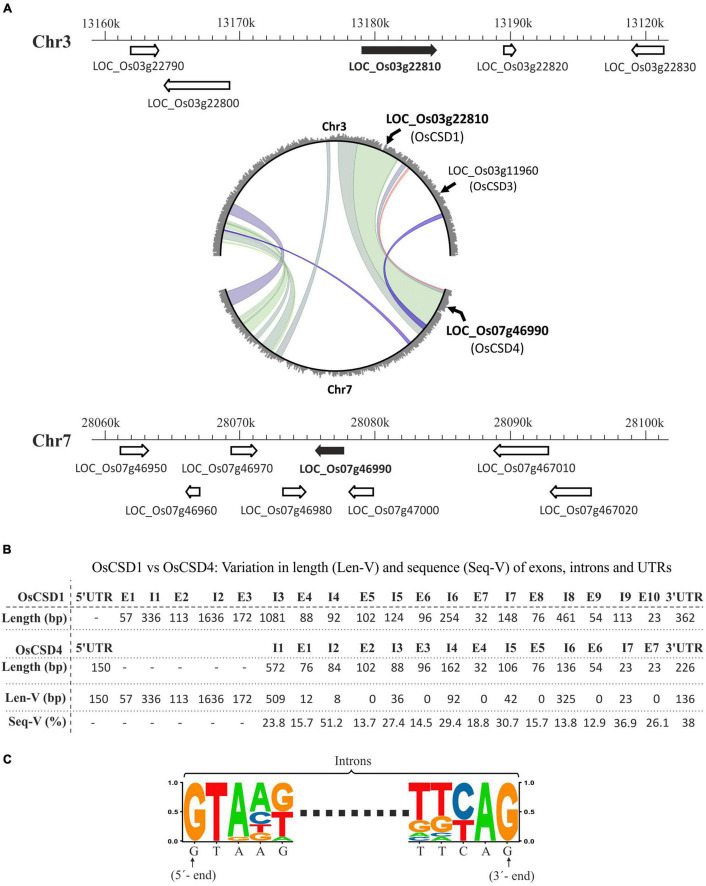
**(A)** Schematic representation of chromosomal locations encoding two rice cytosolic CuZn SODs, *OsCSD1* (LOC_Os03g22810, chromosome 3, Chr3) and *OsCSD4* (LOC_Os07g46990, chromosome 7, Chr7) as per RGAP database. The neighboring genes are shown with arrowheads indicating the direction of the transcription, while the scale indicates the chromosomal coordinates. The circle plot generated at the Monocot PLAZA 4.5 web server shows segmental duplications between chromosomes 3 and 7 along with positions of region-harboring rice cytosolic CSDs (LOC_Os03g22810, LOC_Os07g46990) and peroxisomal CSD (LOC_Os03g11960). **(B)** Overview of variation in length (Len-V) and sequence (Seq-V) between the UTRs, exons, and introns of *OsCSD1* and *OsCSD4* genes. **(C)** Sequence logo generated by aligning the 5′- and 3′-ends of the all introns of *OsCSD1* and *OsCSD4* genes.

The rice genome contains a total of four CSD loci, of which two (LOC_Os03g22810, *OsCSD1* and LOC_Os07g46990, *OsCSD4*) coding for cytosolic CSDs have originated due to an inter-chromosomal block duplication event between chromosomes 3 and 7 (Figure 1A; Thiel et al., 2009; Yadav et al., 2019). The comparison of the two genes in the RGAP database showed heterogeneity in length and organization (UTR-intron-exon). The Os*CSD1* gene is 5.422 kbp long (10 exons, 9 introns, only 3′-UTR) compared to relatively small *OsCSD4* (2.119 kbp: 7 exons, 7 introns, UTRs at 5′ and 3′ ends) (Supplementary Figures 1A,B). The predicted *OsCSD1* cDNA (813 bp) codes for a 270-amino-acid (AA)-long protein compared to *OsCSD4* (cDNA: 459 bp, protein length: 152 AA). The additional 118 AAs at the N-terminal of OsCSD1, contributed by exons 1, 2, 3, and 4 (partially), are not present in OsCSD4 (Supplementary Figure 1C). The analysis of the corresponding regions of *OsCSD1* and *OsCSD4* genes showed heterogeneity. In general, the exon pairs showed conserved length and low sequence variation (13.7–26.1%) compared to the intronic regions (length heterogeneity: 8–509 bp; sequence heterogeneity: 13.8–51.2%) (Figure 1B). However, exceptions were observed in few pairs of exons (e.g., OsCSD1-E4 vs. OsCSD4-E1) and introns (e.g., OsCSD1-I4 vs. OsCSD4-I2 and OsCSD1-I8 vs. OsCSD4-I6) (Figure 1B). 3′-UTRs also showed heterogeneity in the length (136–150 bp) and sequence (38%) (Figure 1B). Despite variations, the donor-acceptor splice site signatures (GT-AG) were conserved in all introns of the two *OsCSDs* (Figure 1C). The introns and UTRs predominantly contributed toward the length variation between the duplicated rice cytosolic CSD genes.

### PCR and *in silico* Analysis Indicate an Inconsistency in the Predicted *OsCSD1* Gene Structure

Oligonucleotide primers were designed to amplify the predicted full-length cDNAs (FL-cDNAs) of *OsCSD1* (CSD1-E1_F_ + CSD1_R_) and *OsCSD4* (CSD4_F_ + CSD4_R_) (Supplementary Table 1 and Supplementary Figures 2A,B). The longer *OsCSD1* FL-cDNA (813 bp) failed to amplify (lane 1, Supplementary Figure 2C, lane 1, Supplementary Figure 2D), whereas *OsCSD4* FL-cDNA (459 bp) was successfully amplified (lane 4, Supplementary Figure 2C). Then, amplification of two overlapping fragments of *OsCSD1* was attempted using combinations of internal and end primers (CSD1-E1_F_ + CSD1-E4a_R_ and CSD1-E4_F_ + CSD1-E1_R_, Supplementary Table 1 and Supplementary Figure 2A) with an aim to generate FL-*OsCSD1* cDNA. However, the region corresponding to exon 1-exon 3 did not yielded any product (lane 2, Supplementary Figure 2D), while the exon4-10 region was amplified successfully (lane 3, Supplementary Figure 2D).

The PCR amplification using genomic DNA template with *OsCSD1* exon-specific forward primers (CSD1-E1_F_, CSD1-E2_F_, CSD1-E3_F_, CSD1-E4_F_) and common reverse primer (CSD1_R_) (Supplementary Table 1 and Supplementary Figure 2A) yielded expected-sized products (Supplementary Figure 2E) and ruled out any problems with primers or assay conditions. Therefore, the predicted *OsCSD1* gene structure and organization were further ascertained using exon specific primers (Supplementary Table 1 and Supplementary Figure 2A). All primer combinations yielded expected-sized products with genomic DNA template (gDNA panel, lanes 1–4, Supplementary Figure 2F). However, with the cDNA template, exons 1, 2, and 3 did not amplify (cDNA panel, lanes 1–3, Supplementary Figure 2F), while exon4-10 yielded expected-sized product (cDNA panel, lane 4, Supplementary Figure 2F). This indicated that the size of *OsCSD1*-cDNA is smaller than the RGAP prediction of 813 bp. The results of *in silico* analysis also supported this contention as the Blastn analysis did not detected any EST entry with similarity in 1–210 bp of exon 1-exon 3 regions of *OsCSD1*-cDNA (Supplementary Figure 3A), no domain was identified in the N-terminal 118 AA region (Supplementary Figure 3B), and no probes were evident in this region for microarrays and SAGE-based transcriptomics approaches (Supplementary Figure 3C). Overall, these results show that the predicted exons 1, 2, and 3 of the *OsCSD1* gene do not correspond to the protein coding region, and exon 1 and exon 2 are not part of the transcript.

### Exon3 Mapping Indicates Two Transcription Start Sites in the *OsCSD1* Gene

**FIGURE 2:**
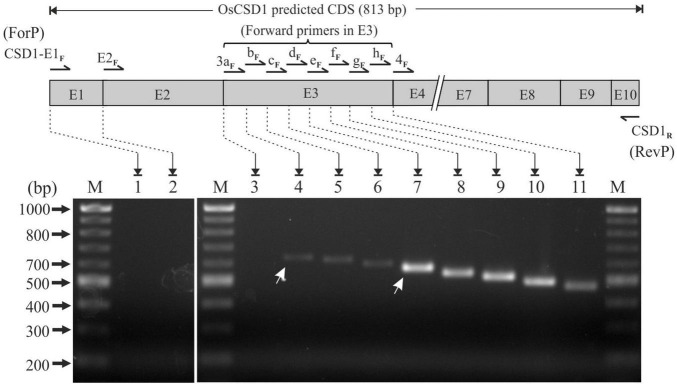
RT-PCR-based analysis of transcription status of *OsCSD1* exon1, exon2, and fine mapping of exon3 (E3 box) using different forward and common reverse primers (CSD1_R_). Lane 1, CSD1-E1_F_ + CSD1_R_; lane 2, CSD1-E2_F_ + CSD1_R_; lane 3, CSD1-E3a_F_ + CSD1_R_; lane 4, CSD1-E3b_F_ + CSD1_R_; lane 5, CSD1-E3c_F_ + CSD1_R_; lane 6, CSD1-E3d_F_ + CSD1_R_; lane 7, CSD1-E3e_F_ + CSD1_R_; lane 8, CSD1-E3f_F_ + CSD1_R_; lane 9, CSD1-E3g_F_ + CSD1_R_; lane 10, CSD1-E3h_F_ + CSD1_R_; lane 11, CSD1-E4_F_ + CSD1_R_. White arrows indicate the two differentially abundant *OsCSD1* transcripts. Lane M, 100 bp DNA ladder.

The use of forward primers in exons 1, 2, and 3 (in combination with CSD1_R_) did not yielded any product in the PCR (lanes 1 and 2, Figure 2). For exon 3, the transcription status of the complete region was evaluated by RT-PCR using eight overlapping forward primers (CSD1-E3a_F_, CSD1-E3b_F_, CSD1-E3c_F_, CSD1-E3d_F_, CSD1-E3e_F_, CSD1-E3f_F_, CSD1-E3g_F_, and CSD1-E3h_F_) in combination with CSD1_R_ (Figure 2 and Supplementary Table 1). Expected-sized PCR products were obtained until primer CSD1-E3b_F_, indicating that complete exon 3 is not part of the *OsCSD1* transcript (lane 3, Figure 2). Interestingly, higher PCR amplification efficiency was observed until primer CSD1-E3e_F_ compared to the primers binding upstream (CSD1-E3b_*F*,_ CSD1-E3c_F_, CSD1-E3d_F_). This indicates a lower abundance of the larger transcript (compare lanes 4–6 and 7–11, Figure 2), which may also be due to two transcription start sites (TSS) in *OsCSD1*. The results of this and the previous section indicate that the *OsCSD1* coding region actually starts from the “ATG” in exon 4 and not from position “1” in exon 1 (RGAP prediction). This makes the *OsCSD1* coding region equal to *OsCSD4* (459 bp, Supplementary Figures 2A,B), and it was used for cloning and overexpression of OsCSD1 and OsCSD4 for comparative analysis.

### *OsCSD1* and *OsCSD4* Genes Showed Differences in Upstream Regulatory Elements

**FIGURE 3:**
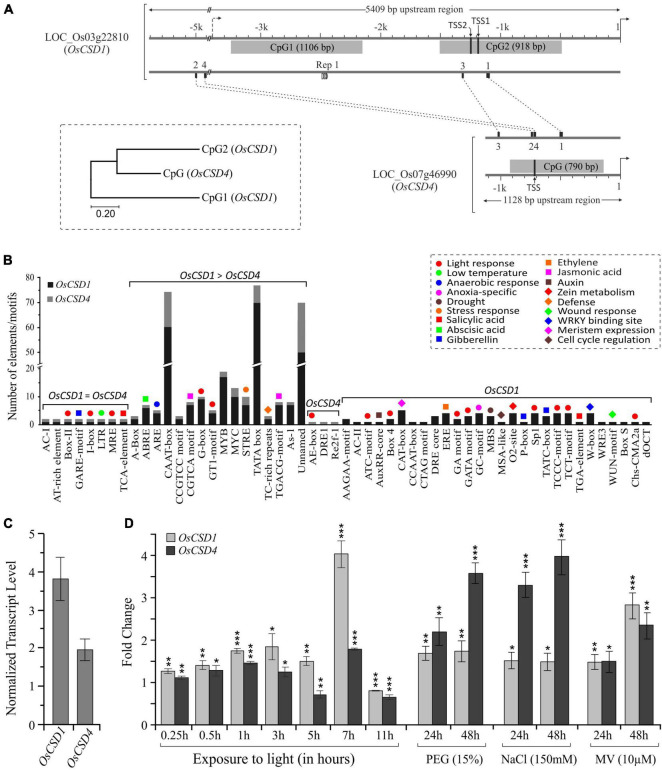
Promoter region differences and expression pattern of *OsCSD1* and *OsCSD4* genes. **(A)** The length and location of CpG islands (gray boxes), positions of conserved motifs (1, 2, 3, 4), repetitive motif (Rep1), and two potential TSS sites in *OsCSD1*-CpG2 and one in *OsCSD4-*CPG are indicated. The scale on the top indicates the length of the upstream region. The dendrogram shows the divergence between the *OsCSD1* and *OsCSD4* CpG islands. **(B)** The bar plot shows the abundance of different categories of *cis*-elements (indicated by color-coded symbols) in the promoter of two *OsCSD*s. Single copy, differentially abundant, and gene-specific *cis*-elements are grouped separately. **(C)** Reverse transcription-quantitative PCR (RT-qPCR)-based analysis of relative abundance of *OsCSD1* and *OsCSD4* transcripts in rice shoot tissue. **(D)** Expression pattern of *OsCSD1* and *OsCSD4* transcripts in light (0–11 h) and in response to polyethylene glycol (PEG, 15%), sodium chloride (NaCl, 150 mM), and methyl viologen (MV, 10 μM) at 24 and 48 h time points after stress treatment. The transcript levels were estimated using actin as a reference gene. The experiment was repeated three times and data are represented as mean value ± SD. Statistical significance is indicated by *(*p* < 0.05), **(*p* < 0.01), ***(*p* < 0.001).

Among the two *CSDs*, the upstream regulatory region of *OsCSD1* was considerably longer (5,409 bp, up to LOC_Os03g22790, Figure 1A) and contained two CpG island regions (CpG1: 1106 bp and CpG2: 918 bp), of which CpG2 contained the two TSS sites (Figure 3A). The *OsCSD4* regulatory region was only 1,128 bp and harbored a single CpG island region (790 bp), which showed more similarity to CpG2 than CpG1 of *OsCSD1* and contained a single TSS (Figure 3A). The upstream regions of *OsCSDs* differed in the relative organization of transcription factor binding sites (TFBs, for bHLH, B3, TCP, bZIP, MYB SAINT, etc.) and a repetitive motif (length: 14 bp, copy number: 2.7) specific to *OsCSD1-*CpG1 island (Figure 3A). The two genes also differed in presence/absence, copy number, and position of *cis*-regulatory elements associated with physiological and stress conditions (Figure 3B and Supplementary Figure 4). The smaller *OsCSD4* upstream region contained ∼50% less *cis*-elements than *OsCSD1* (Figure 3B). Among the common *cis-*elements, eight were single copy (e.g., gibberellin, low temperature, salicylic acid response motifs) and sixteen (e.g., light, jasmonic acid, abscisic acid, anaerobic induction response motifs) showed higher copies in *OsCSD1* (Figure 3B). Twenty-eight motifs (meristem specific expression, anoxia, zein metabolism, auxin response, defense, certain light specific and gibberellin response elements, etc.) were specific to *OsCSD1* and three were specific to *OsCSD4* (Figure 3B). These differences indicate the possibility of the differential response of the *OsCSDs* to abiotic factors, viz. light, salinity, osmotic stress, as observed in this study.

### *OsCSD1* and *OsCSD4* Showed Differential Levels in Tissues and Response to Stress

The analysis of transcript data at RGAP and RED databases revealed the differential expression of rice cytosolic *CSDs* in different tissues and development stages. *OsCSD1* showed higher levels in leaves, pre-emergence inflorescence, anthers, and at the early seed stage, whereas *OsCSD4* was abundant in pistil, embryo, endosperm, and at the late seed stage (Supplementary Figure 5). In the NSICRc106 genotype, *OsCSD1* showed higher transcript levels in shoot tissue (Figure 3C) and rapid as well as higher response (up to ∼four-fold) to light (Figure 3D). *OsCSD4* was more responsive to NaCl and PEG treatments (Figure 3D), and both showed similar responses under oxidative stress (Figure 3D). The impact of coding region heterogeneity on the characteristics of rice OsCSD enzymes was further investigated.

### Structurally/Functionally Important Residues Are Conserved in OsCSD1 and OsCSD4

The NSICRc106 *OsCSD1* and *OsCSD4* cDNAs (GenBank accession numbers: *OsCSD1*, MW091043; *OsCSD4*, MW091044) showed 100% identity to the corresponding Nipponbare cDNA sequences (see text footnote 1). The duplicated OsCSDs showed amino acid substitutions at 18/152 sites due to variations in the exon regions; however, important residues involved in metal ion coordination (Cu^2+^: His-45, His-47, His-62, His-119; Zn^2+^: His-62, His-70, His-79, Aps-82), active site (His-45, His-47, His-62, His-79, Aps-82, His-119), and intra-subunit disulfide bond (Cys-56 and Cys-145) were found to be conserved (Supplementary Figure 1C). Variations were observed at two (AA at 19 and 29) of the total eleven residues of the subunit interaction interface; however, the substituted residues in OsCSD4 (H19F, T29S) were physicochemically similar and capable of similar surface interactions (CDD-NCBI prediction) to form a dimeric protein.

### OsCSD1 and OsCSD4 Exist as a Homodimer in the Native State

**FIGURE 4:**
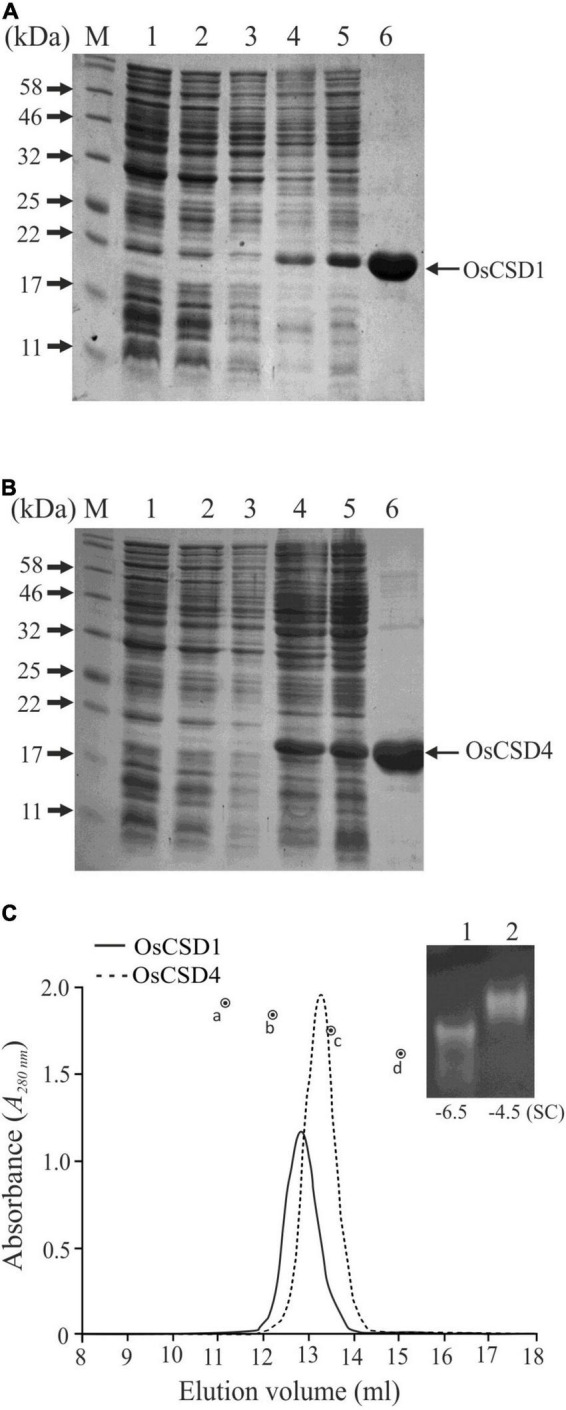
Overexpression and purification of recombinant rice cytosolic OsCSDs. **(A)** OsCSD1: lane 1, pET28a(+) uninduced; lane 2, pET28a(+) induced; lane 3, pET28(+)-OsCSD1 uninduced; lane 4, pET(+)-OsCSD1 induced; lane 5, pET(+)-OsCSD1-induced supernatant fraction; lane 6, Ni-NTA affinity-purified OsCSD1; lane M, Protein molecular weight standard. **(B)** OsCSD4: lane 1, pET28a(+) uninduced; lane 2, pET28a(+) induced; lane 3, pET28(+)-OsCSD4 uninduced; lane 4, pET(+)-OsCSD4 induced; lane 5, pET(+)-OsCSD4-induced supernatant fraction; lane 6, Ni-NTA column-purified OsCSD4; lane M, Protein molecular weight standard. **(C)** Gel-filtration chromatography elution profiles of OsCSD1 and OsCSD4. The standard protein markers used were (a) bovine serum albumin (66.5 kDa), (b) chicken egg albumin (45 kDa), (c) carbonic anhydrase (29 kDa), and (d) cytochrome C (12.4 kDa). The gel photograph shows relative mobility and SOD activities of native OsCSD1 (1) and OsCSD4 (2) in a non-denaturing polyacrylamide gel. “SC” indicates net surface charge on the proteins.

Recombinant OsCSD1 and OsCSD4 were successfully overexpressed in *E. coli* and purified using the Ni-NTA affinity approach (Figures 4A,B). The analysis by gel-filtration chromatography yielded single-peak profiles, indicating that the two OsCSDs exist as homodimer in the native form (Figure 4C). Substitutions at the subunit interaction interface of OsCSD4 (H19F and T29S) showed a minor effect on the profile, but the homodimer status remained unaffected. Native homodimeric configurations of both the OsCSDs were enzymatically active; however, they showed differences in the mobility on native PAGE (Figure 4C). This was likely due to the variation in the net negative charge on the two OsCSDs, which was also predicted by the protein calculator tool (OsCSD1: −6.5; OsCSD4: −4.5, at pH 8). The effect of amino acid variations on SOD characteristics was further investigated.

### Amino Acid Variations Affected Biochemical Characteristics of OsCSDs

**FIGURE 5:**
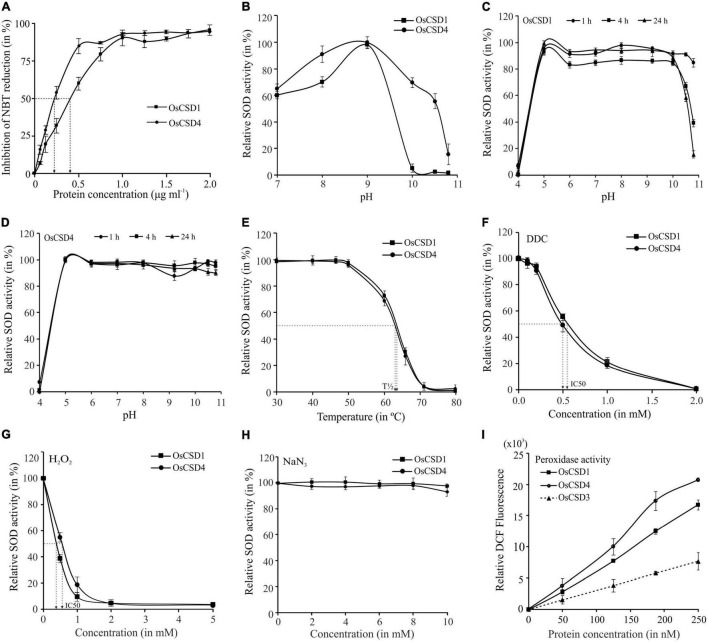
Specific activity, effect of pH, and temperature. **(A)** Estimation of specific activity: increasing protein amount (0–2 μg ml^–1^) was used for SOD assay, and % inhibition of NBT reduction was measured to determine the SOD activity (1 U of SOD activity—amount of protein required to inhibit the NBT reduction by 50%). **(B)** Optimum pH: SOD activity of OsCSDs was assayed at different pH (range: 7.0–10.8) and relative SOD activity was estimated by considering the maximum activity as 100%. **(C,D)** Effect of pH on stability: purified OsCSDs were incubated at different pH (range: 4.0–10.8), aliquots were removed at different time points (1–24 h) and assayed for SOD activity (activity before pre-incubation was considered as 100%). **(E)** Effect of temperature: purified OsCSDs were pre-incubated at different temperatures (30–80°C) for 1 h, and assayed for SOD activity (activity before pre-incubation was considered as 100%), and plotted as a function of temperature. The vertical dotted lines indicate T1/2 value (temperature at 50% of SOD activity is lost). **(F–H)** Effect of SOD inhibitors on enzyme activity, diethyldithiocarbamate (DDC, 0–2.0 mM), hydrogen peroxide (H_2_O_2_, 0–5.0 mM), and sodium azide (NaN_3_, 0–10.0 mM). Relative SOD activity was estimated by considering the enzyme activity before pre-incubation with inhibitor as 100%. The arrows indicate IC50 value (concentration that inhibits 50% of SOD activity). **(I)** For analysis of bicarbonate-dependent peroxidase activity of OsCSDs, dichlorofluorescein (DCF) formation was monitored with increasing amount of protein (0–250 nM). For the SOD activity-based assays, 1U equivalent of purified protein was used, and data are represented as mean ± SD of three independent replicates.

Despite 18 amino acid variations, the duplicated OsCSDs were enzymatically active, with OsCSD4 displaying higher specific activity (4,317 ± 337 U mg^–1^ml^–1^) than OsCSD1 (2,402 ± 171 U mg^–1^ml^–1^) (Figure 5A). The two CSDs showed comparable Vmax (OsCSD1: 4,162 ± 270.3 U mg^–1^ min^–1^; OsCSD4: 3,845 ± 187.9 U mg^–1^ min^–1^) and Km values (OsCSD1: 4.57 ± 1.06 μM; OsCSD4: 3.96 ± 0.74 μM). Both showed similar pH optima (9.0); however, OsCSD4 showed relatively higher SOD activity below and beyond the optimum pH (Figure 5B). Change in pH affected the two OsCSDs to different extents, as both showed complete loss of SOD activity at lower pH and comparable activity between pH 5.0–9.0, but OsCSD1 was affected more at pH > 10.0 (as a function of incubation time) compared to OsCSD4 (Figures 5C,D). The OsCSDs showed similar thermal inactivation profiles with a comparable T1/2 value of 63°C (the temperature at which 50% of the activity is lost) and complete inactivation at 70°C (Figure 5E). They exhibited comparable sensitivity toward SOD inhibitors, DDC, H_2_O_2_, and sodium NaN_3_. DDC inhibited the two OsCSDs to similar extent (IC50, OsCSD1: 0.52 mM; OsCSD4: 0.50 mM) with complete inhibition at 2 mM (Figure 5F). Both showed comparable sensitivity to H_2_O_2_ (IC50, OsCSD1: 0.38 mM, and OsCSD4: 0.55 mM; Figure 5G) and remained unaffected by NaN_3_ treatment (Figure 5H). In addition, the two cytosolic OsCSD harbored bicarbonate-dependent peroxidase activity, with OsCSD4 displaying higher activity than OsCSD1 (Figure 5I) and OsCSD3 isoform characterized previously (Sanyal et al., 2018).

### Secondary Structure Element Variations Affected the Thermostability of OsCSDs

**FIGURE 6:**
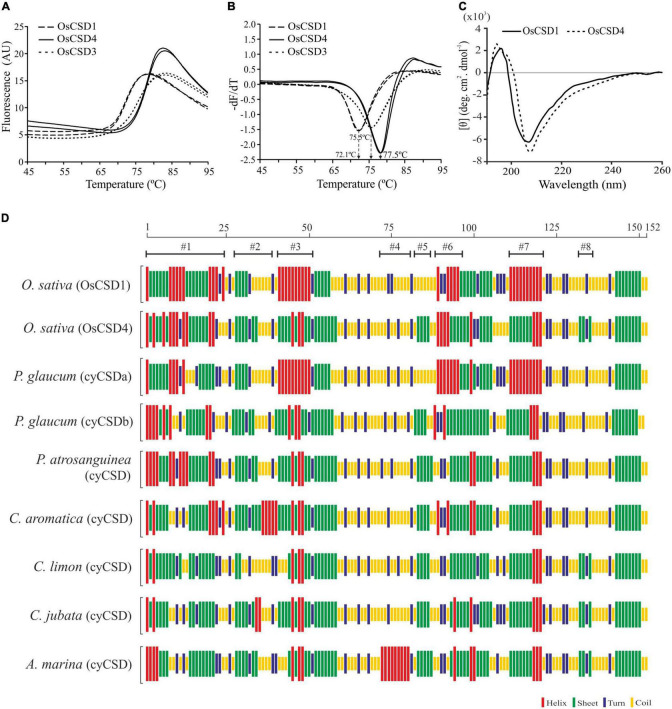
Differential scanning fluorimetry (DSF) and secondary structure analysis. **(A)** DSF profiles of OsCSD1, OsCSD4, and OsCSD3 generated using a Roche LightCycler LC480 II real-time PCR system. **(B)** First derivative curve (-dF/dT vs. Temperature) generated using the Tm calling software function for estimation of melting temperature (Tm) of respective OsCSDs, as indicated by arrows. **(C)** Circular dichroism spectra of OsCSD1 and OsCSD4 (parameters, path length: 0.5 cm, Acq duration: 0.2 s, and band width: 2.0 nm). **(D)** Analysis of plant cytosolic CSDs (cyCSDs) of *Oryza sativa* (OsCSD1 and OsCSD4, this study), *P*. *glaucum* (cyCSDa, ABP65325, Mahanty et al., 2012), *P*. *glaucum* (cyCSDb, this study), *P*. *atrosanguinea* (EU532614, Kumar et al., 2012), *C*. *aromatica* (FJ5896638, Kumar et al., 2014), *C*. *limon* (AF318938, Lin et al., 2002), *C*. *jubata* (EF530044, Kumar et al., 2016b), and *A*. *marina* (ACA50531.1, Fesharaki-Esfahani et al., 2021) by the CFSSP online tool. The scale on the top indicates the position of amino acids while #1-#8 indicate the regions affected with substitutions.

Differential scanning fluorimetry (DSF) analysis showed that OsCSD4 with a higher melting temperature (Tm: 77.5°C) is more thermostable than OsCSD1 (Tm: 72.1°C) (Figures 6A,B) and OsCSD3 (Tm value: 75.5°C) isoform characterized previously (Sanyal et al., 2018). Although circular dichroism (CD) analysis showed CuZn SOD-specific signatures (high β-sheet, low α-helix content) in both, some discernible differences in shape and/or intensity of spectra (increase of the signal of OsCSD4 at ∼210 nm, shift in the intensity and peak maximum at 196 nm toward 193 nm) indicated differences in secondary elemental regions in two OsCSDs (Figure 6C). The analysis based on CFSSP online tool identified local variations in OsCSD1 and OsCSD4 due to amino acid substitutions. Four major (#1, #3, #6, #7) and three minor (#2, #5, #8) region-specific variations in OsCSD4 seem associated with its higher stability (Figure 6D). CFSSP-based identification of similar local variations among some more plant cytosolic CSDs with differential thermostability supported the above contention (Figure 6D). The thermostable cytosolic CSDs of *Potentilla atrosanguinea*, *Curcuma aromatica*, *Citrus limon*, *Caragana jubata*, and *Avicennia marina* showed patterns similar to OsCSD4, whereas relatively less thermostable *Pennisetum glaucum* CSD (designated as cyCSDa) matched OsCSD1 pattern (Figure 6D). A second uncharacterized cytosolic CSD in *P. glaucum* (referred to as cyCSDb) with a pattern similar to OsCSD4 is likely to be more thermostable than cyCSDa; however, this needs to be verified experimentally. These results indicate that the presence of certain amino acids can cause local changes in the secondary elements, and contribute toward enhanced stability as seen in OsCSD4 and reported thermostable cytosolic CSDs of other plants.

### OsCSD1 and OsCSD4 Showed Structural Variations in Two Important Loop Regions

**FIGURE 7:**
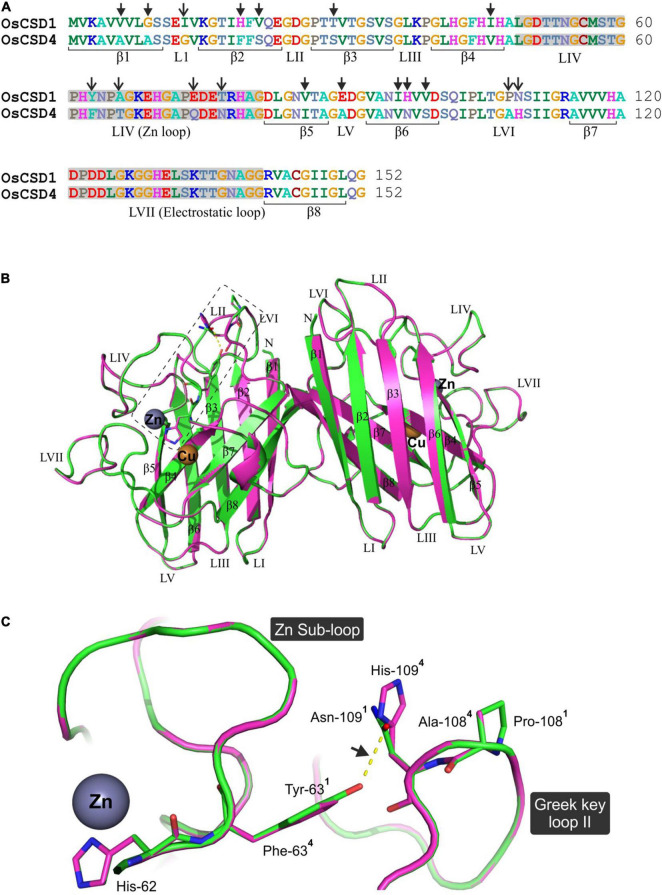
Comparative *in silico* structural analysis of two rice cytosolic CSDs. **(A)** Pair-wise alignment of OsCSD1 and OsCSD4 amino acid sequences. The arrows show the positions of 18 amino acid variations affecting the beta strands (β) and loop (L) regions. **(B)** Dimeric superposed 3D homology models of OsCSD1 (green) and OsCSD4 (magenta) generated using the SWISS MODEL workspace using *S. lycopersicum* CuZn SOD crystal structure (PDB ID: 3PU7) as template. Beta strands (β1- β8), loops (LI-LVII), and positions of Cu and Zn metal co-factors are indicated. **(C)** Enlarged view of the rectangular region (marked in **B**) to show the effect of amino acid substitutions on structural variations in Zn sub-loop and Greek key loop II regions of OsCSD1 and OsCSD4. The superscripts indicate the residues in two proteins (^1^: OsCSD1 and ^2^: OsCSD4), while an arrow shows H-bond between Asn-109 and Tyr-63 (OsCSD1).

The homodimeric homology models of OsCSD1 and OsCSD4 were generated using *Solanum lycopersicum* CuZn SOD template (PDB ID: 3PU7) and superposed to assess the structural impact of 18 amino acid variations on OsCSDs (Figure 7A). Each of the subunits folded as an eight-stranded, Greek-key β-barrel with seven connecting loops (typical of CuZn SODs); however, when superposed, the OsCSDs showed structural differences in certain regions (Figure 7B). The amino acid variations were localized to six β-strands (β1: 2, β2: 2, β3: 1, β4: 1, β5: 1, β6: 3) and four loops (LI: 1, LIV/Zn loop: 4, LV:1, LVI/Greek-key loop-II: 2), which comprise important structural features of CSDs (Figure 7A). Of the four amino acid variations (Y63F, A66T, E74Q, and T77N) in loop LIV (connects strands β4 and β5), Y63F follows a bridging histidine (His-62) that coordinates with Cu^2+^ and Zn^2+^ (Figure 7C). Structural differences were also evident in the Greek-key loop II region due to interaction differences between Tyr-63 and Asn-109 (OsCSD1) compared to Phe-63 and His-109 (OsCSD4). Additionally, the presence of Pro-108 (OsCSD1) or Ala-108 (OsCSD4) also seems to affect the local conformation in OsCSD1 and OsCSD4 proteins (Figure 7C).

### OsCSD1 and OsCSD4 Subunits Interact to Yield Enzymatically Active Heterodimeric OsCSD

**FIGURE 8:**
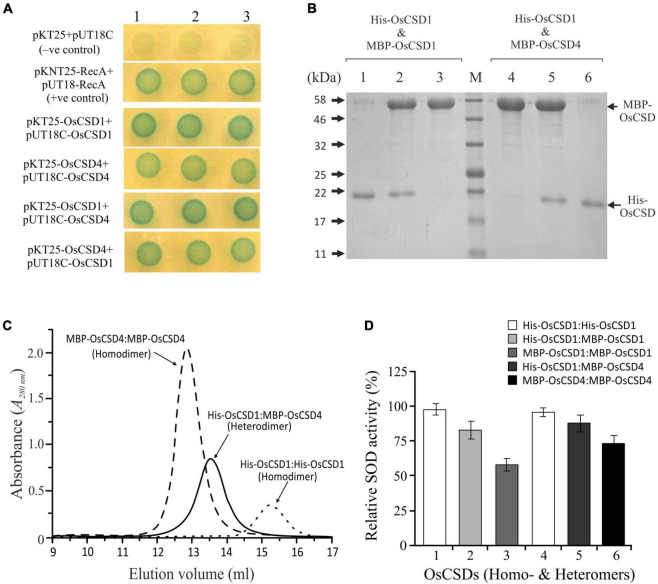
Analysis of heteromeric interaction between rice OsCSD1 and OsCSD4 subunits by BACTH system. **(A)** *E. coli* (BTH101) cells co-transformed with different combinations of pKT25 and pUT18C plasmids expressing OsCSD1 and OsCSD4 fusion proteins were analyzed by spot test assay. Cells containing empty plasmids served as negative control, while the positive control contained plasmids pKNT25-RecA + pUT18-RecA. **(B)** SDS-PAGE analysis of heterodimer and homodimer forms separated after Ni-NTA/amylose affinity purification of co-expressed OsCSDs, lane 1, His-OsCSD1:His-OsCSD1 (homodimer); lane 2, His-OsCSD1:MBP-OsCSD1 (heterodimer); lane 3, MBP-OsCSD1:MBP-OsCSD1 (homodimer); lane 4, MBP-OsCSD4:MBP-OsCSD4 (homodimer); lane 5, His-OsCSD1:MBP-OsCSD4 (heterodimer); lane 6, His-OsCSD1:His-OsCSD1 (homodimer); lane M, protein molecular weight standards. **(C)** Gel-filtration profiles of two different homodimers (MBP-OsCSD4:MBP-OsCSD4 and His-OsCSD1:His-OsCSD1) and heterodimer (His-OsCSD1:MBP-OsCSD4) forms. **(D)** Analysis of SOD activity of OsCSD homodimer and heterodimer forms by NBT reduction method.

The duplicated rice OsCSD1 and OsCSD4 exist as homodimer; however, as both are cytosolic, the possibility of heteromeric interaction was investigated by BACTH assay. *E. coli* (BTH101) cells transformed with two different plasmid combinations (pKT25-OsCSD1 + pUT18C-OsCSD4 and pKT25-OsCSD4 + pUT18C-OsCSD1), and co-expressing fusion proteins T18-OsCSD1/OsCSD4 and T25-OsCSD4/OsCSD1, restored the adenylate cyclase function. This showed that the subunits of two OsCSDs are capable of heteromeric interaction (Figure 8A); however, whether the heterodimer harbor SOD activity was not evident. An approach involving cloning OsCSDs into two different plasmids (pET28a: His-Tag, Kan*^R^* and pMAL-c5x: MBP-Tag, Amp*^R^*), co-transformation, and expression into *E. coli* (Supplementary Figure 6) successfully separated the heterodimeric OsCSD (subunits with His-tag and MBP-tag) from the homodimeric forms (both subunits with MBP-tag/His-tag) (Figures 8B,C). The OsCSD1:OsCSD4 heterodimer was found to be enzymatically active; however, the presence of bulky MBP Tag (42 kDa) affected the activity in both the MBP-OsCSD heterodimer (MBP Tag in one subunit) and MBP-OsCSD homodimer (MBP tags in Figure 8D).

### Overexpression of OsCSD1 and OsCSD4 Enhanced Oxidative Stress Tolerance of *Escherichia coli* Cells

**FIGURE 9:**
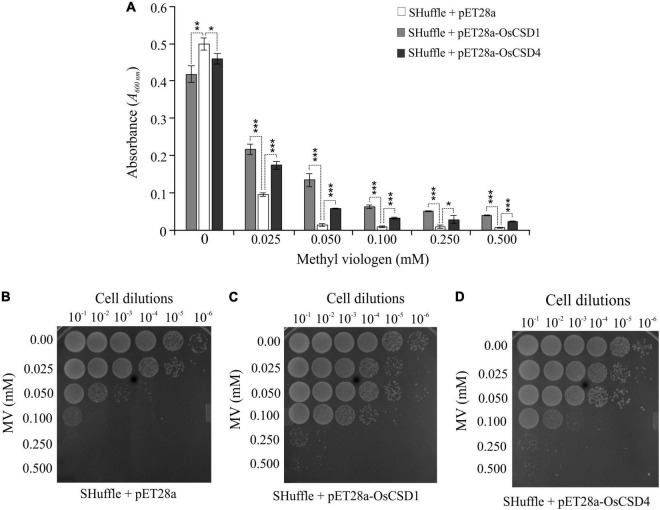
Analysis of oxidative stress tolerance of *E. coli* SHuffle T7 Express cells overexpressing OsCSD1 and OsCSD4. **(A)** Comparative analysis of growth of *E. coli* cells (absorbance at 600 nm) containing pET28a (empty vector), pET28a-OsCSD1, and pET28a-OsCSD4 plasmids. Cells induced with IPTG were treated with increasing methyl viologen concentration (0–0.500 mM) and monitored spectrophotometrically. The experiment was repeated three times and data are represented as mean value ± SD. Statistical significance is indicated by *p < 0.05, **p < 0.01, ***p < 0.001. **(B–D)** Spot test assay for evaluation of oxidative stress tolerance of *E. coli* SHuffle T7 Express cells containing empty pET28a **(B)**, pET28a-OsCSD1, **(C)** and pET28a-OsCSD4 (D). Cells were induced with IPTG, treated with methyl viologen (0.0–0.500 mM), and analyzed by spot test. The numbers on the top indicates the serial dilution of the *E. coli* cells.

The oxidative stress protection function of the two rice cytosolic CSDs was evaluated in *E. coli* cells containing either empty pET28a or pET28a-OsCSD1/OsCSD4. IPTG-induced *E. coli* cells subjected to no MV showed similar growth and viability (Figures 9A–D), which was substantially reduced with an increase in MV concentration (Figures 9A,B). Heterologous overexpression of OsCSD1 and OsCSD4 enhanced the tolerance of *E. coli* toward MV-mediated oxidative stress, as evident from the cell growth (Figure 9A) and spot test analysis (Figures 9B–D).

### Block Duplications Are Predominant Events Responsible for Multiple CSDs in Plants

**FIGURE 10:**
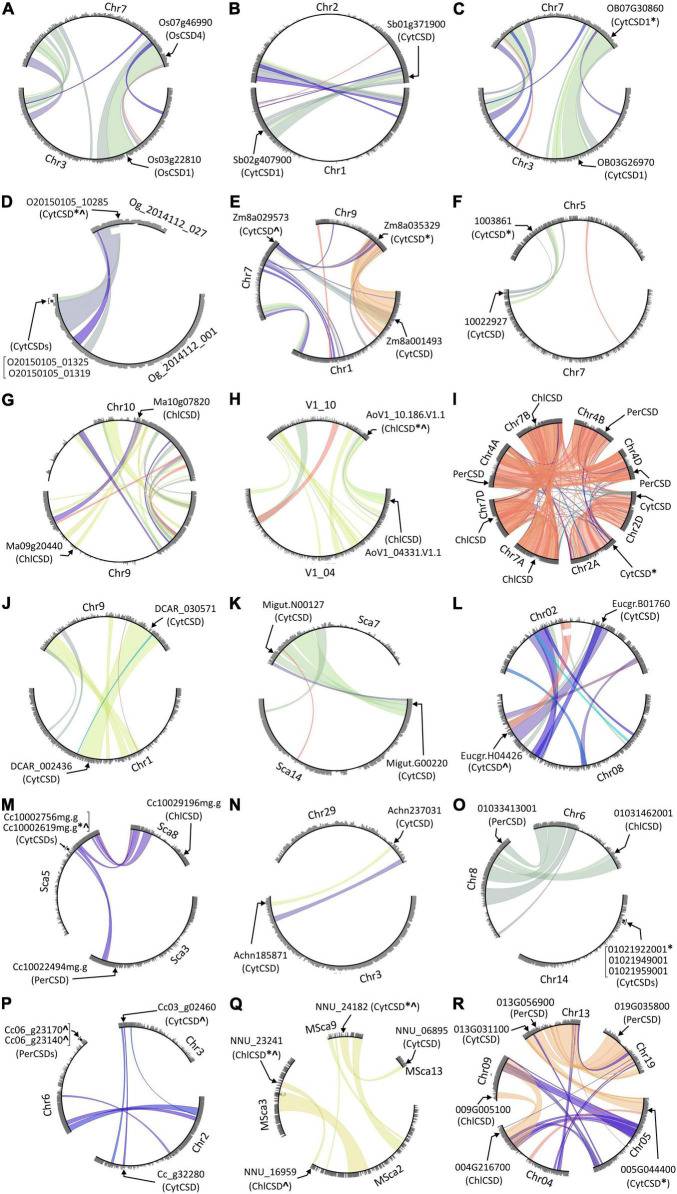
Circle plots generated at PLAZA database showing tandem/block duplications of cytosolic (CytCSDs; OsCSD1, OsCSD4 in rice), chloroplastic (ChlCSD), and peroxisomal (PerCSDs) CSD isoforms among some monocots [**(A)** *O. sativa*, **(B)** *S. bicolor*, **(C)** *O. brachyantha*, **(D)** *O. thomaeum*, **(E)** *Z. mays* (PH207), **(F)** *C. americanus*, **(G)** *M. acuminata*, **(H)** *A. officinalis*, and **(I)** *T. aestivum*] and dicots [**(J)** *D. carota*, **(K)** *E*. *guttata*, **(L)** *E. grandis*, **(M)** *C. clementina*, **(N)** *A. chinensis*, **(O)** *V. vinifera*, **(P)** *C. canephora*, **(Q)** *N. nucifera*, and **(R)** *P. trichocarpa*]. In general, the chromosomes involved in block/tandem are shown with designations (Chr, chromosome; LG, linkage group; Sca, scaffold, etc.) and gene ids as per the PLAZA database. The arrows indicate the location of CSDs, “*” indicate copies with variations in SOD domain/important features, and “^” indicate copies containing variation due to indels.

In view of the impact of block-duplication event on cytosolic rice OsCSDs, the prevalence of similar events on CSDs was investigated among 28 monocot and 46 dicot genomes at PLAZA database using InterPro id: IPR018152 (superoxide dismutase, copper/zinc, binding site). Duplicated CSDs were detected in 17 monocots and 23 dicots, with inter-chromosomal block duplications being more prevalent (35–40%) than tandem (up to 6.4%) and combined events (block + tandem, up to 1.2%). Among the CSDs, the cytosolic isoforms (CytCSDs) were more affected than chloroplastic (ChlCSDs)/peroxisomal (PerCSDs) types in both monocots and dicots (Figure 10). Block duplications also generated additional ChlCSDs or PerCSDs, except in *Coffea canephora* where a tandem event was involved (Figure 10P). Six dicots harbored more than one duplicated CSD (CytCSD + ChlCSD/PerCSD), while *Triticum aestivum*, *Saccharum spontaneum*, and *Populus trichocarpa* contained extra copies of all the three isoforms (Figures 10I,R and Supplementary Tables 3, 4).

### Length and/or Sequence Heterogeneity Affected Important Features of Duplicated CSD Copies

Multiple alignment of 92 cytosolic CSDs (monocots: 43; dicots: 49) including duplicates identified 15 (#1-#15) major indels (Supplementary Figure 7). The CSD copies among monocots were more divergent (“d” range: 0.105, *Sorghum bicolor* to 0.412, *Zea mays*) than in dicots (“d” range:0.007, *Vitis vinifera* to d: 0.075, *P*. *trichocarpa*). Minor variation in length (<5 bp) and sequence (≤13% sites) among CSD copies did not affect the structurally/functionally important features as evident from the domain analysis at CDD-NCBI (Supplementary Tables 3, 4 and Supplementary Figures 8, 9). However, higher length and/or sequence variation among CSD copies (both monocots and dicots) affected important SOD features, viz. active site (*Oryza brachyantha*, *Zostera japonica*), Cu^2+^ binding site (*S*. *spontaneum; Cajanus cajan*, *V*. *vinifera*), Zn^2+^ binding site (*O*. *brachyantha*, *Z*. *japonica*, *T*. *aestivum*), disulfide bond (*Elaeis guineensis*, *Oropetium thomaeum, C*. *cajan*), subunit interaction interface (*O*. *thomaeum*, *Z*. *mays*, *Asparagus officinalis*, *T*. *aestivum*, *S*. *spontaneum*; *C*. *cajan*, *P*. *trichocarpa*, *Actinidia chinensis*), and disruption of SOD domain (*Cenchrus americanus*, *Nelumbo nucifera*) (Supplementary Tables 3, 4 and Supplementary Figures 8, 9). Interestingly, cytosolic CSD copies of *Z*. *mays*, *V. vinifera*, and *Malus domestica* harbored additional domains along with the SOD domain (Supplementary Tables 3, 4). A similar effect on the characteristic CSD features was evident among the few divergent peroxisomal/chloroplastic CSD duplicates in both the plant groups (Supplementary Tables 3, 4 and Supplementary Figures 8, 9).

### Phylogenetic Analysis Revealed a Differential Divergence of Duplicated CSDs

**FIGURE 11:**
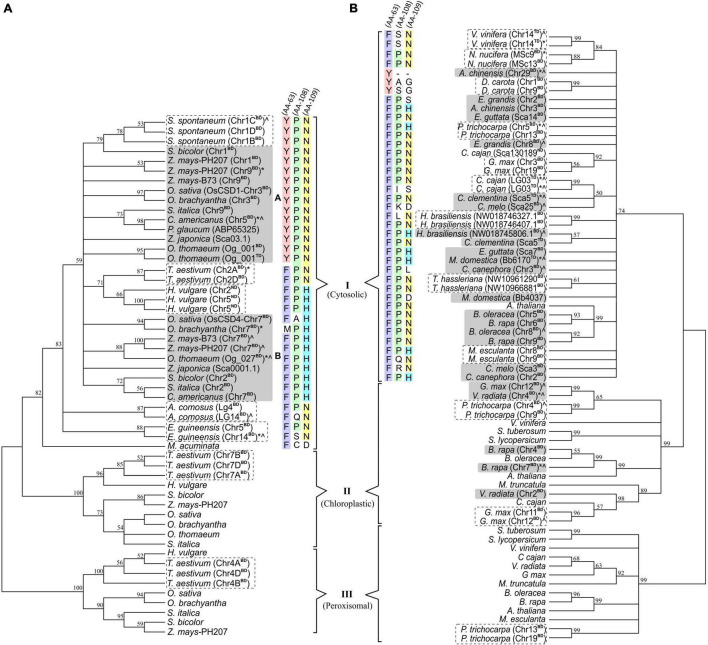
Phylogenetic analysis of cytosolic, chloroplastic, and peroxisomal CSDs (including duplicate copies) among monocots **(A)** and dicots **(B)** using neighbor-joining algorithm (pairwise-deletion option) in the MEGA software. Clusters specific to cytosolic (I), chloroplastic (II), and peroxisomal (III) CSD isoforms are indicated, and the numbers at the nodes represent bootstrap values (in%) for a 500-replicate analysis. Taxa designation includes the species name followed by chromosomal designation and duplication type (BD: block duplication and TD: tandem duplication) in parenthesis and symbols indicating extent of variation (“*” variation in SOD domain/important features; “^” variation due to indels). Conservation of amino acid residues at three important positions (63 in Loop IV; 108 and 109 in Loop VI) are shown with different color codes for cytosolic CSD copies in monocots and dicots.

Phylogenetic analysis of 53 monocot and 68 dicot-specific CSDs (including block/tandem duplicates) placed the sequences into three clusters (I, II, III) corresponding to cytosolic, chloroplastic, and peroxisomal isoforms (Figure 11). Most monocot cytosolic CSD duplicates were divergent (d range: 0.105–0.412; OsCSD1/OsCSD4, d: 0.126) and placed in different subgroups (A and B), while the less divergent (d: 0.007–0.089) copies of *S*. *spontaneum*, *T*. *aestivum*, *Ananas comosus*, and *E. guineensis* remained together (Figure 11A). On the contrary, duplicate cytosolic CSDs in most dicots (d range: 0.007–d: 0.175) clustered together, and only in few dicots (*A. chinensis*, *Eucalyptus grandis*, *Erythranthe guttata*, *Citrus clementina*, *Cucumis melo*, *C. canephora*, *M. domestica*), the divergent copies were placed in different subgroups of cluster I (Figure 11B). Likewise, the duplicate ChlCSD copies in *Glycine max*, *Vigna radiata*, and *Brassica rapa* showed high divergence and were placed in separate subgroups (cluster II, Figure 11B), whereas less divergent ChlCSD and PerCSD copies of *T*. *aestivum* and *P*. *trichocarpa* were grouped together in respective clusters (Figures 11A,B). Interestingly, amino acids at structurally/functionally important sites (AA 63, 108, and 109) that differed in OsCSD1 and OsCSD4 (Figure 7) showed distinct patterns among monocot CSD duplicates specific to subgroup A [combination: Tyr(63)-Pro(108)-Asn(109) and subgroup B (combination: Phe(63)-Pro(108)-His(109)] (Figure 11A). Such a distinction was not observed among dicot-specific CSD duplicates, where Phe(63)-Pro(108)-Asn(109) was the most common combination, and species-specific CSD copies in separate subgroups contained different combinations (Figure 11B).

## Discussion

This study investigated the effect of post-duplication divergence on the characteristics of two block-duplicated rice cytosolic CSDs and the prevalence of similar events in other plants. Multiple SOD isoforms involved in cellular ROS homeostasis and oxidative stress protection (Halliwell, 2006) have evolved due to the interplay of multiple factors (Alscher et al., 2002; Miller, 2012; Dreyer and Schippers, 2019). Among plant genomes, duplication events have contributed toward the evolution of divergent gene copies with altered expression, characteristics, or novel function (Flagel and Wendel, 2009; Barker et al., 2012; Qiao et al., 2019). The two rice cytosolic CSDs retained the SOD function but diverged toward differential response and protein characteristics, whereas many other plants contained substantially divergent CSD copies indicative of loss of SOD characteristics and/or functional divergence.

Antioxidant systems maintain cellular ROS homeostasis and play important roles in physiological processes, stress responses, and protection from oxidative damage (Gill and Tuteja, 2010; Nath et al., 2013; Mittler, 2017; Leonowicz et al., 2018). Multiple, compartment-specific SODs are crucial for local O_2_^⋅–^ scavenging, modulation of ROS/RNS levels, inter-organellar and cellular signaling, and cellular redox environment (Gill et al., 2015; Leonowicz et al., 2018; Wang et al., 2018; Jankù et al., 2019; Hasanuzzaman et al., 2020). The SOD function is also important for the H_2_O_2_ gradient and flux, and modulation of redox-sensitive pathways (Wang et al., 2018; Jankù et al., 2019; Hasanuzzaman et al., 2020). The most abundant CuZn SOD isoforms (CSDs) are present in multiple compartments including cytosol (Yesbergenova et al., 2005) and respond to diverse conditions including biotic and abiotic stresses (Jagadeeswaran et al., 2009; Kim et al., 2012; Lightfoot et al., 2017; Sanyal et al., 2018; Tyagi et al., 2019; Saini et al., 2021).

Since duplication event-generated gene copies often diverge to different extents and fates (Barker et al., 2012), it was important to investigate the effect of divergence on important characteristics of CSDs in rice and other plants. Rice genome harbor 24 duplicated blocks, of which duplicated segments on chr3 and chr7 contain *OsCSD1* and *OsCSD4 loci* (Thiel et al., 2009; Yadav et al., 2019). The database-predicted gene structure variation of the two *OsCSDs* suggested post-duplication divergence. However, experimental validation and *in silico* analysis showed that the annotation of the predicted *OsCSD1* exon 1-3 region seems incorrect. The actual *OsCSD1* coding region is of the same length (459 bp) as *OsCSD4* and is similar to RAP-DB prediction (Supplementary Figure 10). Hence, the RGAP-predicted *OsCSD1* model needs revision to avoid prediction errors during the comparative analysis, as seen in the analysis at the Indica Rice Database (IRDB).^16^

Further, the *OsCSD1* 5′-UTR was also found to be longer than the RAP-DB prediction (102 bp) and harbored two TSSs (TSS1 and TSS2) in the *OsCSD1-*CpG2 island (Figure 3A). The CpG islands often support dispersed transcription initiation events (Deaton and Bird, 2011), which was also evident in *OsCSD1*, but the upstream TSS2 was relatively a weak transcription initiator than TSS1 (Figure 2). The prediction of additional putative TSSs (TSSPlant tool) in *OsCSD1* and a higher abundance of smaller *OsCSD1* ESTs (NCBI-EST database) supports the above contention (Supplementary Figure 3). An assessment of modulation of efficiency of the two TSSs under different conditions and the effect of the extra 5′-UTR region on transcript dynamics is worth investigating for insights into the expression and regulation of *OsCSD1*. Additionally, the differences in the upstream regulatory regions (promoter length/sequence, CpG islands, TFBS, *cis-*elements) play important roles in regulation at transcription level (Haak et al., 2017). Collectively, these factors seem important for differential response of the two cytosolic *OsCSDs* in the present and previous reports (Kaminaka et al., 1999; Morita et al., 2011; Kim et al., 2012; Sanyal et al., 2018; Saini et al., 2021).

The *OsCSD1* and *OsCSD4* divergence has also affected the post-transcriptional control mediated by miR398 that regulates *CSDs* under different conditions (Sunkar et al., 2006; Jagadeeswaran et al., 2009; Beauclair et al., 2010; Zhu et al., 2011; Guan et al., 2013; Li et al., 2019, 2020). The sequence divergence has affected the miR398 target sites of the two *OsCSDs*, with differential cleavage efficacy (Li Y. F. et al., 2010), which might lead to different transcript levels. An additional layer of regulation is mediated by a natural antisense transcript that controls the biogenesis of miR398 (Li et al., 2020). The sequence divergence also affected the splicing pattern of the duplicated *OsCSDs*, with *OsCSD4* yielding two transcripts differing at 5′-UTR (Saini et al., 2021; see text footnote 1), which might affect the transcript characteristics or regulation (Mignone et al., 2002). Recent studies have shown the importance of Arabidopsis AtFSD3 alternative isoforms in chloroplast development (Lee et al., 2019).

Variations in the coding region did not affect the functionally important amino acids (Robinett et al., 2018) in the two OsCSDs, and the activities were at par with the reported plant CSDs (Mahanty et al., 2012; Mishra et al., 2014; Sanyal et al., 2018). Both enhanced the oxidative stress tolerance of the *E. coli* cells, as reported for other recombinant plant SODs (Krieger-Liszkay et al., 2011; Mahanty et al., 2012; Sanyal et al., 2018). The response to SOD inhibitors and loss of activity due to low pH-mediated effect on subunit interaction/cofactor leaching (Mahanty et al., 2012; Mishra et al., 2014; Tuteja et al., 2015) was also comparable. However, OsCSD4 displayed higher specific activity, and the sequence variations seem associated with its stability at higher pH and temperature. The effects on characteristics due to non-critical amino acid changes might be associated with the observed local structural variations in SODs (Lin et al., 2002; Kumar et al., 2012, 2014, 2016b; Mahanty et al., 2012; Mishra et al., 2014; Tuteja et al., 2015; Sanyal et al., 2018; Fesharaki-Esfahani et al., 2021).

The differences in CD spectral signatures of the OsCSDs were indicative of variations in sequence/secondary structure elements, as often reported in other CSDs (Fridovich, 1975; Lyons et al., 1998; Mishra et al., 2014; Tuteja et al., 2015; Sanyal et al., 2018). The comparative CFSSP analysis of cytosolic CSDs of rice (OsCSD4, OsCSD1), *C. limon*, *P. atrosanguinea*, *C. aromatica*, *C. jubata*, and *A. marina*, identified regions likely to be associated with stability (Figure 6). It also predicted higher stability of *P. glaucum* cyCSDb (block duplicate of cyCSDa) and can be utilized for such analysis. Overall, the two OsCSDs displayed structural features typical of CuZn SODs (Yogavel et al., 2008; Perry et al., 2010). Variations in LIV and proximity of Y63F to His-62 (conserved bridging residue that divides LIV into disulfide and Zn-binding sub-loops) seem crucial for inter-related stabilities of the sub-loops (Shin et al., 2009). Moreover, certain sites (AA108: OsCSD1-Pro/OsCSD4- Ala) and bonds (H-bond: OsCSD1-Tyr-63 and Asn-109, Greek-key loop) may affect the Zn sub-loop and GKL-II dynamics, while the interaction between His-109 (OsCSD4) and Gln-102 (and rotamer flip possibility) might alter the active-site channel (Yogavel et al., 2008). These variations might be responsible for differences in the OsCSD characteristics. It is also evident that amino acids flanking the CSD core are crucial for retaining activity after denaturation (Kumar et al., 2016a).

This study also revealed heteromeric interaction capability between the two cytosolic OsCSDs, which might offer a certain functional advantage, as reported in the case of three chloroplastic Fe SODs (AtFSDs) in Arabidopsis (Gallie and Chen, 2019). While AtFSD1 localizes to multiple compartments under different conditions (Dvoøák et al., 2021), its block duplicate, AtFSD2, forms a heterocomplex with AtFSD3, which is important for chloroplast development and oxidative stress protection (Myouga et al., 2008). The OsCSD1:OsCSD4 heterodimer might have certain physiological significance, as it is enzymatically active, and the constituent CSDs differ in characteristics like specific activity, stability, and surface charge. However, deciphering the role of OsCSD1:OsCSD4 heterodimer in cytosolic SOD function, if any, will need further analysis.

**FIGURE 12:**
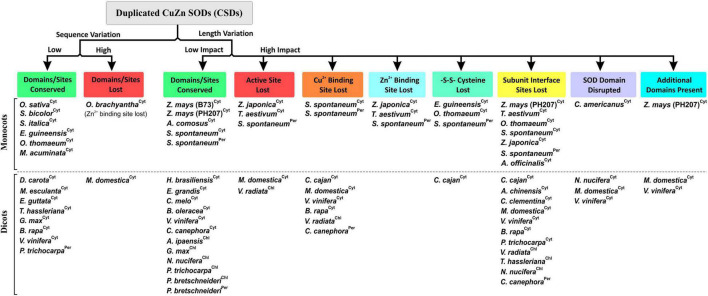
Simplified grouping of divergence-mediated effects on the important features of duplicated copies of CuZn SOD isoforms (Cyt, cytosolic; Per, peroxisomal; Chl, chloroplastic) among monocots and dicots.

The effect of sequence divergence on characteristics of block-duplicated rice cytosolic CSDs was intriguing; however, the impact of such events on CSDs of other plants is relatively less known. Most plant genomes have undergone duplication events leading to multiple gene copies, which often diverge for different fates, viz. neofunctionalization, subfunctionalization, deletion, or pseudogenization (Flagel and Wendel, 2009; Barker et al., 2012; Qiao et al., 2019). These possibilities were also evident among duplicated CSD copies of different plants. While functional partitioning seems likely for the retention of duplicated cytosolic CSDs in rice, along with six monocots and eight dicots, post-duplication deletion (Flagel and Wendel, 2009) of duplicated copies was evident among 40% of monocots and 50% of dicots analyzed. Moreover, CSD duplicates with additional N-/C-terminal region and typical SOD features may form heterodimers (with normal CSD subunits), where the extra region might contribute toward novel interactions/functions, as reported for AtFSD2:AtFSD3 heteromeric complex in Arabidopsis (Myouga et al., 2008). Furthermore, in both the plant groups, high divergence affected critical SOD features, of which some were more prevalent, viz. subunit interaction interface (19 CSDs), Cu^2+^ binding site (09), active site (06), disulfide bond (04), SOD domain (04), and Zn^2+^ binding site (04), while three CSDs harbored additional domains (Figure 12). It would be interesting to see whether these altered CSDs interfere with cellular SOD function or harbor novel functions. Disruption of subunit interaction affected the spectral and enzyme characteristics of *Ipomoea carnea* CSD (Mishra et al., 2014).

The clustering pattern of monocot cytosolic CSDs indicated a likelihood of a duplication event in a primitive ancestor. A whole genome duplication event (∼20 Mya), suggest to have occurred before the divergence of certain cereal crops (Thiel et al., 2009), might be responsible for the monocot-specific pattern. Subsequently, species-specific evolution patterns might have led to the retention, divergence, or loss of extra CSD copies. For example, cytosolic CSD copy in *O. brachyantha* (genus *Oryza*) accumulated high-sequence variation and lost the Zn^2+^ binding site (and part of active site) compared to less divergent copies in *Setaria italica* and *S*. *bicolor*, which belong to different genera. Distinct divergence was also evident in residues (AA 63, 108, and 109) among the monocot- and dicot-specific CSD copies, suggesting different time scales of duplication events in the two plant groups.

This study investigated the impact of duplication events on cytosolic CuZn SODs in plants by experimental analysis of two block-duplicated cytosolic rice CSDs and *in silico* analysis of duplicated CSD copies among 74 plant genomes. The rice CSDs displayed altered expression, regulation, post-transcriptional regulation, and enzyme characteristics, as a consequence of post-duplication divergence but retained the SOD function. Moreover, the heteromeric interaction capability might be crucial for dynamics for cytosolic SOD function in rice. Duplicated CSDs in few species showed retention of conserved features, whereas in most other plants, high divergence resulted in the loss of domains/sites, disrupted SOD domains, or novel domains, indicating complete/partial loss of SOD activity or functional diversification. It is important to decipher the functional significance of CSDs lacking typical features, viz. co-factor binding sites and interaction interface. CSDs with single co-factor are reported in bacteria and fungi (Robinett et al., 2018), but the lack of a cofactor may also lead to its inactivation (Nedd et al., 2014). Additional CSDs might also show non-redundant functions mediated by specific interactions or post-translational modifications (PTMs), which might be crucial for physiology, developmental, and stress responses (Yamakura and Kawasaki, 2010; Lallemand et al., 2020). In view of these important aspects of duplication events, this study advocates further investigations for insights into the complex regulation and functioning of SODs in plants.

## Data Availability Statement

The datasets presented in this study can be found in online repositories. The names of the repository/repositories and accession number(s) can be found in the article/Supplementary Material.

## Author Contributions

RPS performed the experiments, analyzed the results, and wrote the manuscript. VP conducted the biophysical analysis and homology modeling and analyzed the results. NJ and RS planned the experiments, analyzed and reviewed the data. HM planned the experiments, analyzed and reviewed the data, and wrote the manuscript. AS conceived the idea, planned and conducted the experiments, analyzed the data, wrote the manuscript, and communicated. All authors contributed to the article and approved the submitted version

## Conflict of Interest

The authors declare that the research was conducted in the absence of any commercial or financial relationships that could be construed as a potential conflict of interest.

## Publisher’s Note

All claims expressed in this article are solely those of the authors and do not necessarily represent those of their affiliated organizations, or those of the publisher, the editors and the reviewers. Any product that may be evaluated in this article, or claim that may be made by its manufacturer, is not guaranteed or endorsed by the publisher.

## Footnotes

1
http://rice.uga.edu/

2
http://rice.plantbiology.msu.edu/

3
https://rapdb.dna.affrc.go.jp/

4
https://blast.ncbi.nlm.nih.gov/Blast.cgi

5
http://www.ncbi.nlm.nih.gov

6
https://bioinformatics.psb.ugent.be/plaza/

7
http://web.expasy.org/compute_pi/

8
http://protcalc.sourceforge.net/

9
http://www.biogem.org/tool/chou-fasman/index.php

10
https://www.ncbi.nlm.nih.gov/Structure/cdd/wrpsb.cgi

11
http://PlantPAN.itps.ncku.edu.tw/

12
http://bioinformatics.psb.ugent.be/webtools/plantcare/html/

13
http://www.softberry.com/

14
http://expression.ic4r.org

15
http://swissmodel.expasy.org/workspace

16
http://www.genomeindia.org/irdb
