# Supplementary material for: Molecular and Biochemical Analysis of Duplicated Cytosolic CuZn Superoxide Dismutases of Rice and in silico Analysis in Plants
Source: Front Plant Sci. 2022 May 30;13:864330. doi: 10.3389/fpls.2022.864330 (PMC9191229; doi:10.3389/fpls.2022.864330)
Supplement: Supplementary file 1 [file Image_1.pdf]

Supplementary Figure 1: Predicted gene structure organization of *loci* encoding two rice cytosolic CuZn Superoxide dismutase (*CSD*) genes as per Rice Genome Annotation Project database (http://rice.uga.edu). (A) LOC_Os03g22810 (*OsCSD1*), (B) LOC_Os07g46990 (*OsCSD4*). Regions corresponding to the exons/coding regions (blue boxes, indicated by 'E'), introns (connecting lines), and UTRs (white boxes) are indicated. Scale on the top indicates the chromosomal coordinates. (C) Sequence alignment of the RGAP predicted amino acid sequences of OsCSD1 and OsCSD4. Predicted OsCSD1 is 270 AA long, while OsCSD4 is 152 AA long (length variation: 118 AA). Important residues are indicated by different coloured arrows: co-ordination with Cu^{2+} (red) and co-ordination with Zn^{2+} (green), intra-subunit disulfide bond (purple), variations at subunit interaction interface (black).

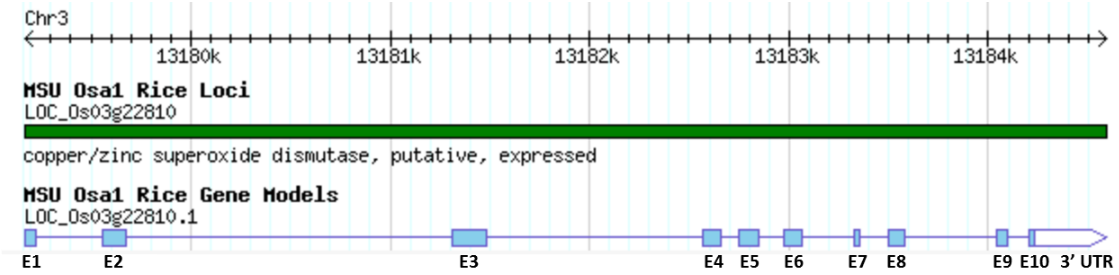

(A)

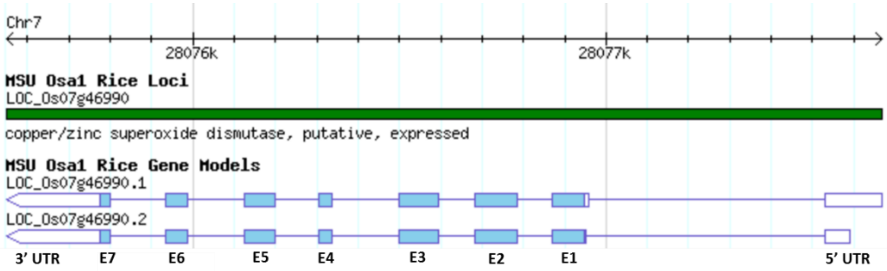

(B)

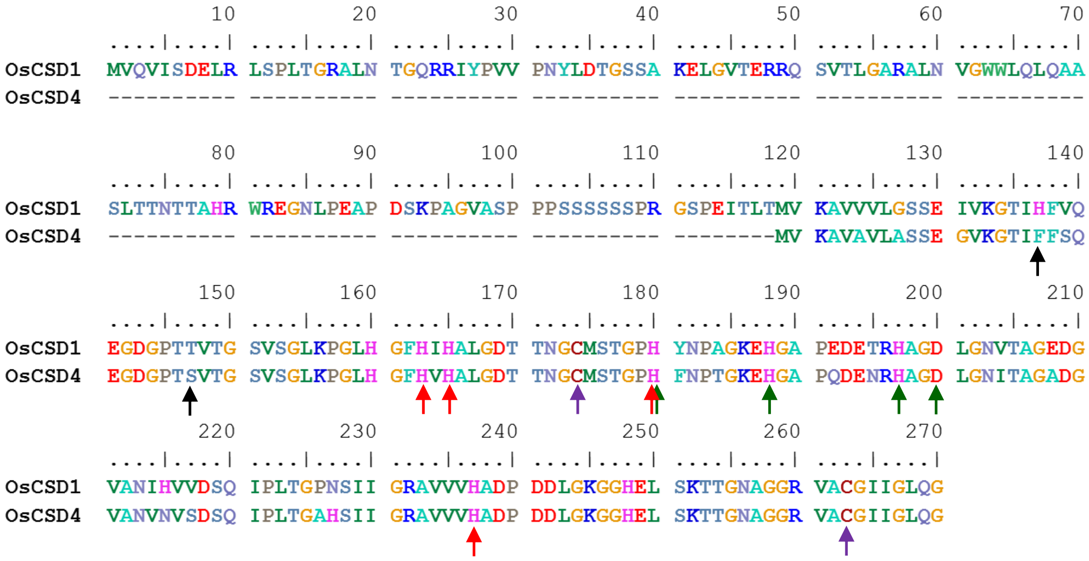

(C)

Supplementary Figure 2: Schematic representation of RGAP-predicted gene structures (exon-intron-UTR organization) of rice cytosolic *CSD* genes: (A) of LOC_Os03g22810 (*OsCSD1*) and (B) LOC_Os07g46990 (*OsCSD4*). Exons (grey boxes), introns (thick dashed line), UTRs (white boxes), and start codons (ATG) in the *OsCSD1* (w.r.t. cDNA) and *OsCSD4* genes are shown along with length of the coding region. Positions of various forward and reverse oligonucleotide primers used for PCR amplification of FL-cDNAs (RGAP predicted) and individual exons are also shown. (C-F): PCR amplification of *OsCSD1* and *OsCSD4* FL-cDNA, and validation of exon organization of RGAP predicted *OsCSD1* gene. (C) FL-cDNA amplification of *OsCSD1* (primers: CSD1-E1$_F$ + CSD1$_R$) and *OsCSD4* (primers: CSD4$_F$ and CSD4$_R$) in '+RT' (with reverse transcriptase) and '-RT' control (without reverse transcriptase) samples. (D) PCR amplification of different *OsCSD1* cDNA regions, exon1-10 (lane 1, primers: CSD1-E1$_F$ + CSD1$_R$), exon1-4 (lane 2, primers: CSD1-E1$_F$ + CSD-E4a$_R$) and exon4-10 (lane 3, primers: CSD1-E4a$_F$ + CSD1$_R$). (E) PCR amplification of *OsCSD1* regions using genomic DNA template and different primers: CSD1-E1$_F$ + CSD1$_R$ (lane 1), CSD1-E2$_F$ + CSD1$_R$ (lane 2), CSD1-E3a$_F$ + CSD1$_R$ (lane 3), CSD1-E4$_F$ + CSD1$_R$ (lane 4). (F) PCR amplification of *OsCSD1* exon/ exon-combinations with genomic DNA (left panel) or cDNA templates (right panel), and different primers: CSD1-E1$_F$ + CSD1-E2a$_R$ (lane 1); CSD1-E2$_F$ + CSD1-E2$_R$ (lane 2); CSD1-E3a$_F$ + CSD1-3$_R$ (lane 3); CSD1-E4$_F$ + CSD1$_R$ (lane 4). Lane M indicates DNA markers, 100 bp ladder (Figure B, D, F) and 500 bp ladder (Figure E).

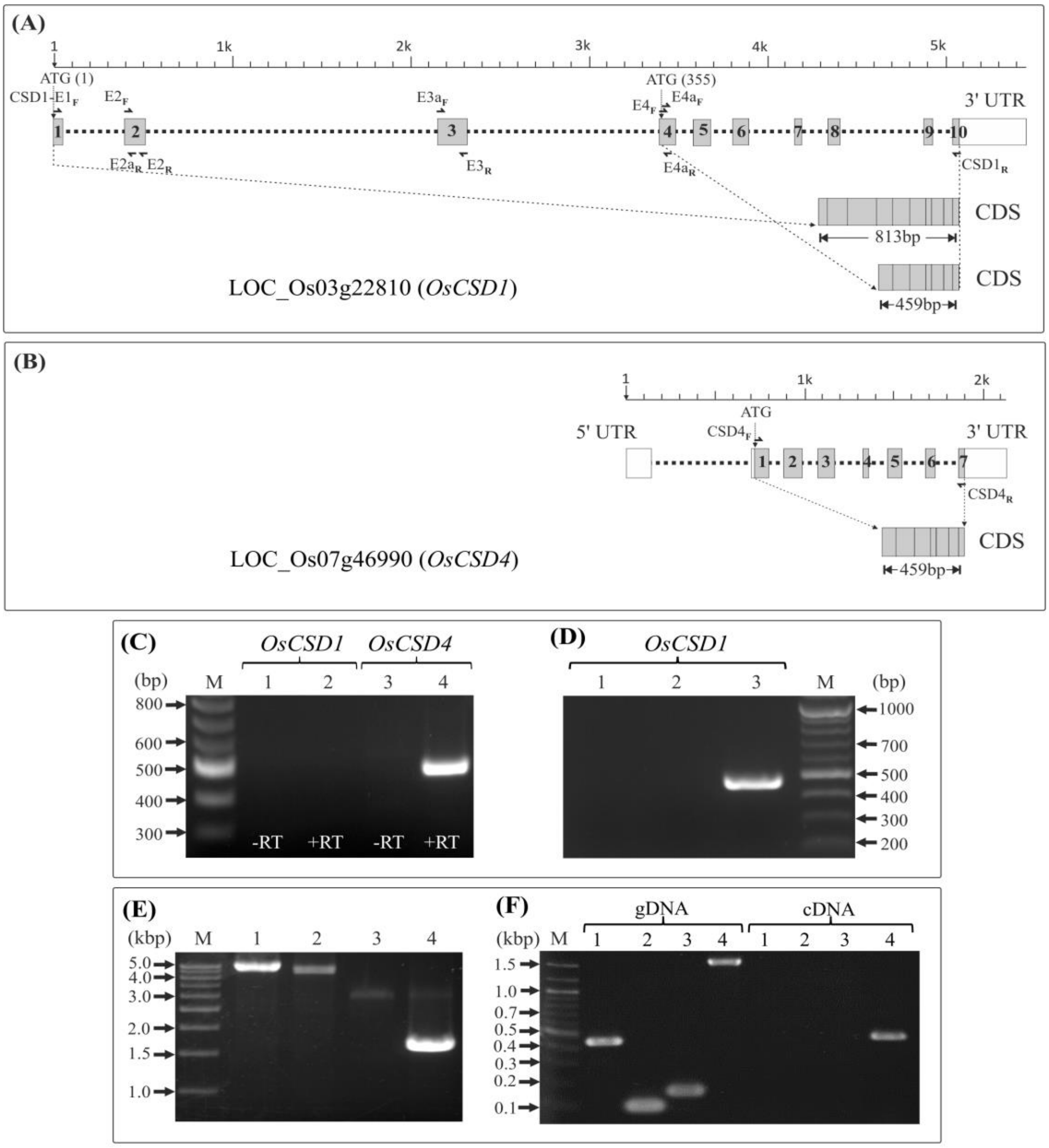

Supplementary Figure 3: Database search analysis of cDNA region (and encoded amino acid sequence) of the region corresponding to exon1, exon2, exon3, and exon4 (till ATG at 355) of the RGAP predicted *OsCSD1* gene structure. (A) Top hits obtained in 'Blastn' search of the *OsCSD1* 1-354 bp cDNA region. No similarity observed in region 1-210 bp with any available EST. (B) Results of conserved domain analysis (at CDD-Blast, NCBI) of RGAP predicted OsCSD1 protein sequence (length: 270 AA). No conserved domains detected in the N-terminal 118 AA region of OsCSD1. (C) Schematic representation of location of SAGE Tags and microarray probes (Affymetrix and Agilent) along the length of RGAP predicted *OsCSD1* gene structure. No Tags/probes are designed/utilized in exon1-exon4 region.

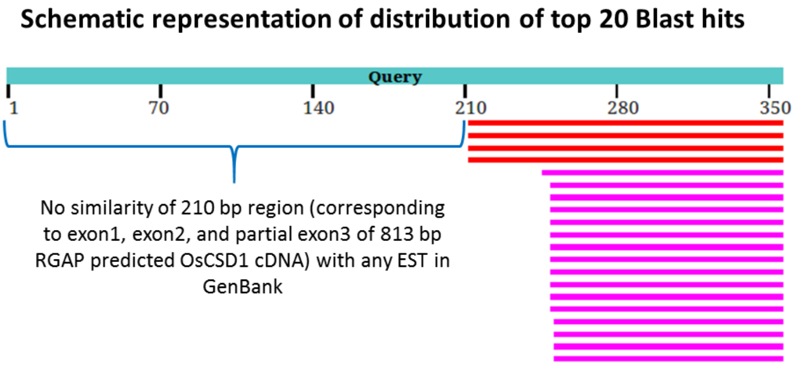

(A)

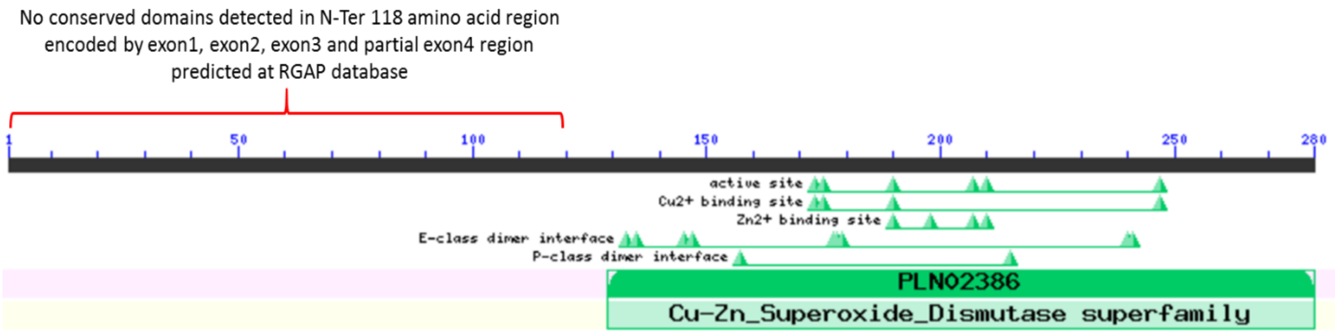

(B)

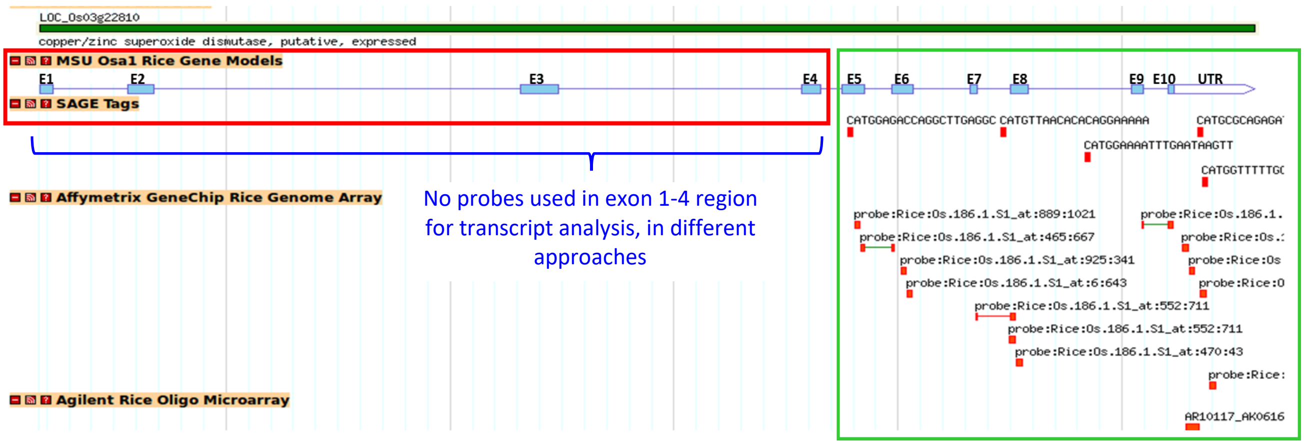

(C)

Supplementary Figure 4: Locations of relative positions of the cis- regulatory elements in the upstream region of rice cytosolic CSD genes, (A) *OsCSD1* and (B) *OsCSD4*. Analysis of *cis*-elements was carried at PlantCARE database (http://bioinformatics.psb.ugent.be/webtools/plantcare/html). Scale at the top is indicative of relative positions of *cis*-elements relative to the translation start site (+1), and ‘+’ and ‘-’ indicates the plus and minus strands, respectively. Positions of common promoter elements (TATA, CAAT etc.) and unnamed motifs are not indicated.

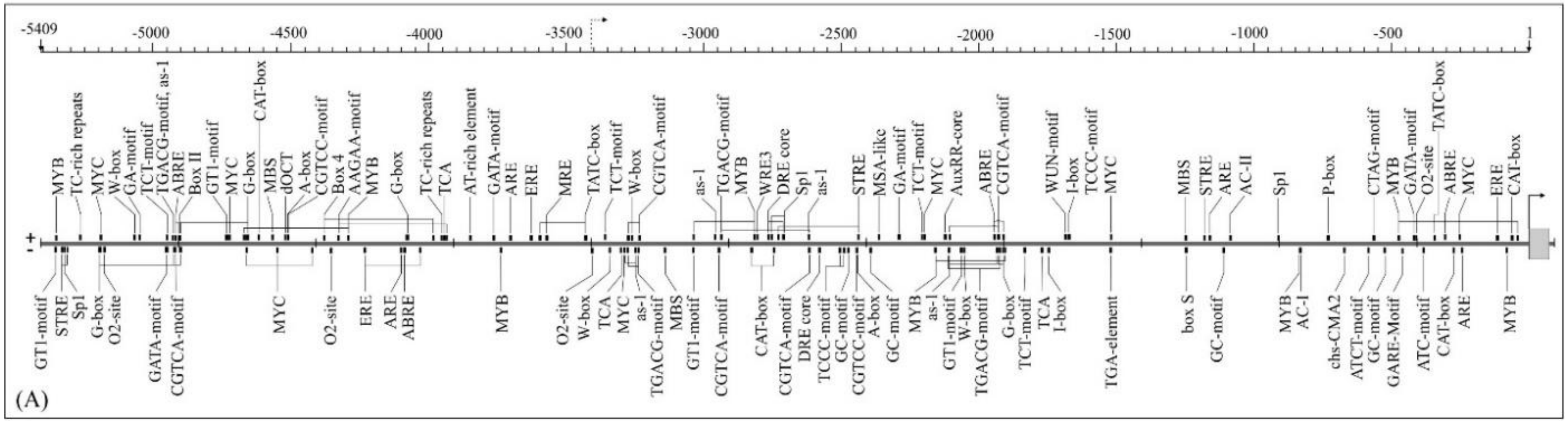

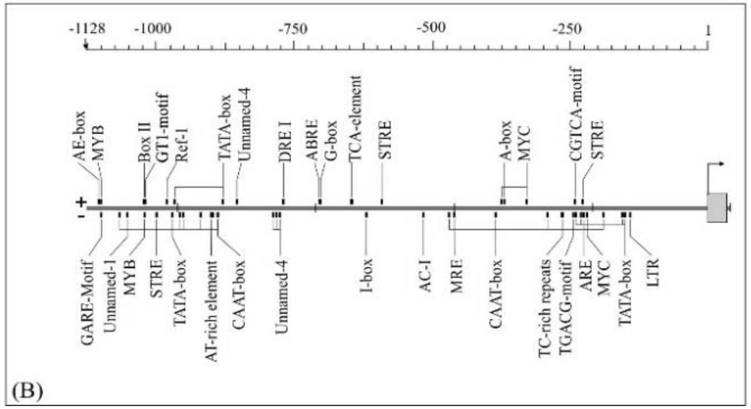

Supplementary Figure 5: Relative transcript levels of two rice cytosolic CSDs, *OsCSD1* and *OsCSD4*: (A) Transcript levels of *OsCSD1* and *OsCSD4* in different rice tissues/developmental stages as per RGAP database (http://rice.uga.edu). (B-C) Expression level of *OsCSD1* and *OsCSD4* transcripts in different tissues/developmental stages as per rice expression database (RED, http://expression.ic4.org). FPKM: Fragments per kilo base per million mapped reads.

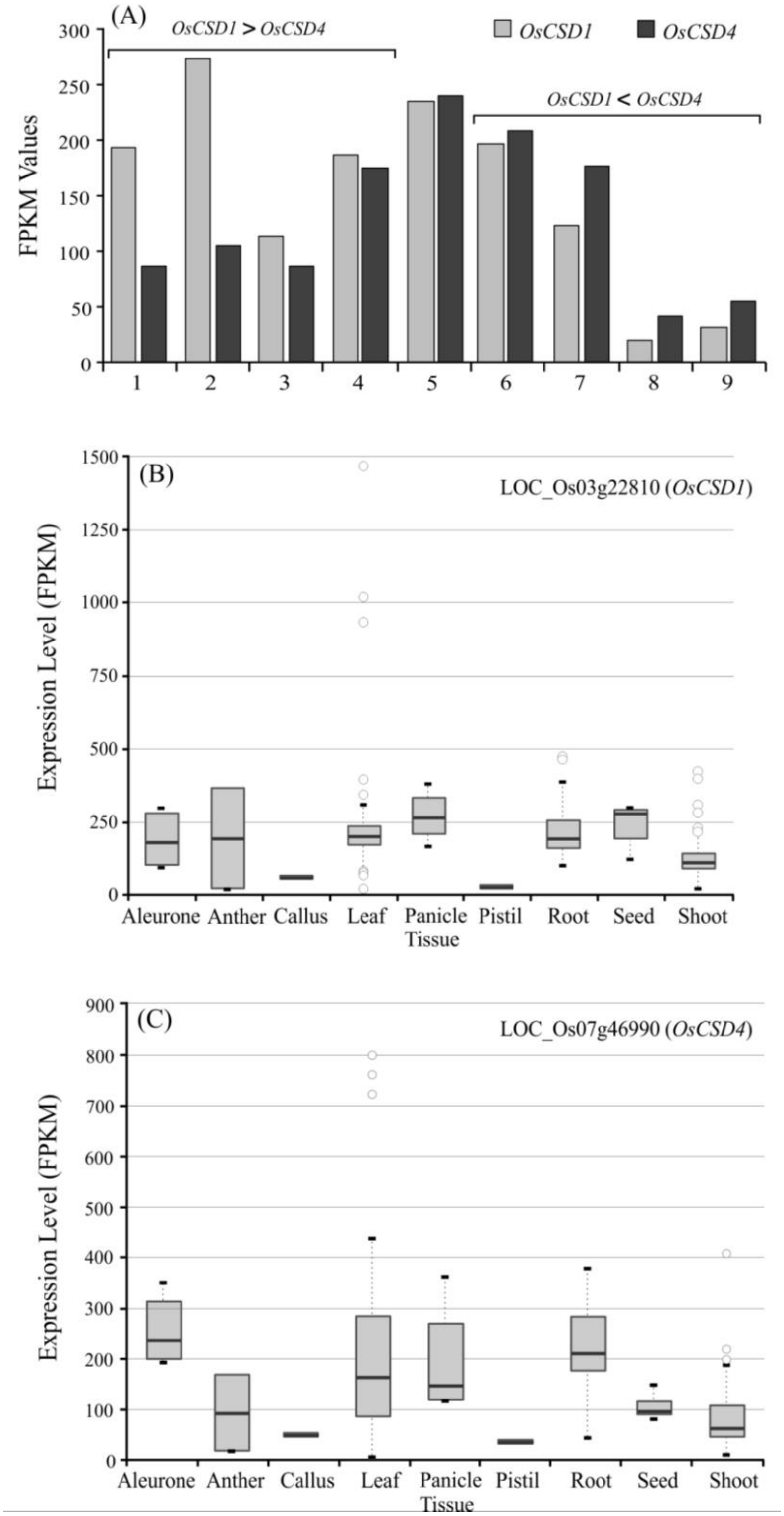

1 : Leaves- 20 days
2 : Pre-emergence inflorescence
3 : Anther
4 : Seeds- 5 days after pollination
5 : Post-emergence inflorescence
6 : Pistil
7 : Embryo- 25 days after pollination
8 : Endosperm: 25 days after pollination
9 : Seeds- 10 days after pollination

Supplementary Figure 6: Schematic representation of approach for co-expression and isolation of homodimeric and heterodimeric subunit configurations of OsCSD1 and OsCSD4. His-tagged OsCSD1 (cloned in pET28 plasmid, kanamycin selection marker) and MBP-tagged OsCSD4/OsCSD1 (cloned in pMAL-c5x, ampicillin selection marker) were co-transformed in *E. coli* SHuffle T7 Express host and over-expressed. Cells were lysed and homodimers (His-OsCSD1:OsCSD1, MBP-OsCSD4:MBP-OsCSD4) and heterodimer (His-OsCSD1:MBP-OsCSD4) subunit configurations were isolated and purified by combination of affinity (Ni-NTA and Amylose based) and gel-filtration purification methods. For convenience different colour codes are used for the two affinity tags (His: dark blue; MBP: light blue-green) and OsCSDs (OsCSD1: red; OsCSD4: green).

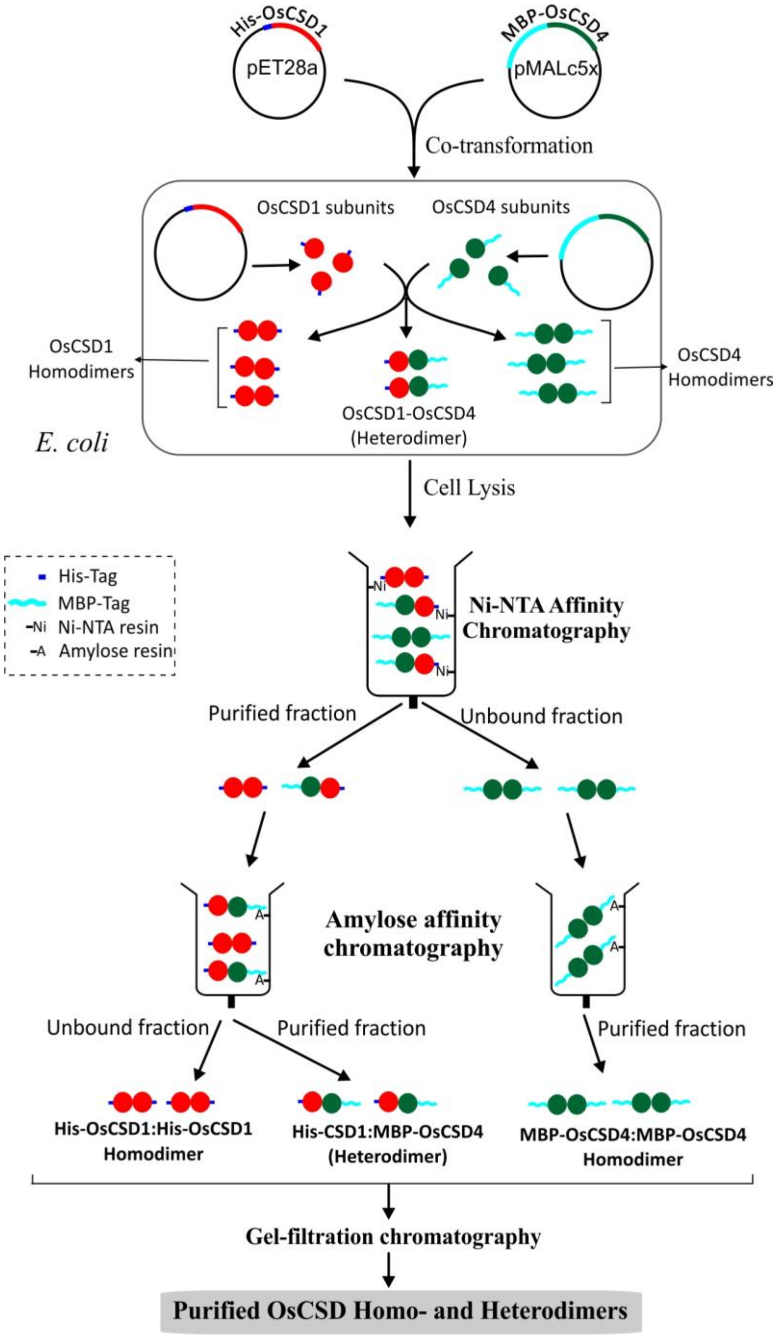

Supplementary Figure 7: Multiple sequence alignment (MSA) of 92 cytosolic CuZn SODs (monocots: 1-43, dicots: 44-92) retrieved from the PLAZA web resource for monocot (https://bioinformatics.psb.ugent.be/plaza/versions/plaza_v4_5_monocots/) and dicots (https://bioinformatics.psb. ugent.be/plaza/versions/plaza_v4_5_dicots/). MSA was carried out using ClustalX software (Thompson et al., 1997) using default gap opening and gap extension penalties, followed by alignment editing by BioEdit Software (Hall, 1999). The name of the species is followed by genomic location (chromosome/linkage group/scaffold etc.) in the parenthesis. For convenience, the duplicated sequences (due to block/tandem duplication) in a genome are arranged next each other. Regions (#1- #15) with length variations are indicated below the alignment. (*Note: C. sim*, *C. simplicifolius*; *H. bra*, *H. brasiliensis*; *T. has*, T. *hassleriana*).

```
                                           10         20         30         40         50         60         70
                                   ....|....| ....|....| ....|....| ....|....| ....|....| ....|....| ....|....|
Monocots
O.sativa(Chr3-CSD1)                ---------- ---------- ---------- ---------- ---------- ---------- ----------
O.sativa(Chr7-CSD4)                ---------- ---------- ---------- ---------- ---------- ---------- ----------
O.brachyantha(Chr3-CSD)            ---------- ---------- ---------- ---------- ---------- ---------- ----------
O.brachyantha(Chr7-CSD)            ---------- -------MLQ SAHHLLLLLL SSKGSPEFRS TGCRALQDWR FWSSSVSNFQ YELLASQVVT
S.italica(Chr2-CSD)                ---------- ---------- ---------- ---------- ---------- ---------- ----------
S.italica(Chr9-CSD)                ---------- ---------- ---------- ---------- ---------- ---------- ----------
S.bicolor(Chr1-CSD)                ---------- ---------- ---------- ---------- ---------- ---------- ----------
S.bicolor(Chr2-CSD)                ---------- ---------- ---------- ---------- ---------- ---------- ----------
E.guineensis(Chr5-CSD)             ---------- ---------- ---------- ---------- ---------- ---------- ----------
E.guineensis(Chr14-CSD)            ---------- ---------- ---------- ---------- ---------- ---------- ----------
O.thomaeum(001-CSDa)               ---------- ---------- ---------- ---------- ---------- ---------- ----------
O.thomaeum(001-CSDb)               ---------- ---------- ---------- ---------- ---------- ---------- ----------
O.thomaeum(027-CSD)                ---------- ---------- ---------- ---------- ---------- ---------- ----------
Z.mays(B73)(Chr7-CSD)              ---------- ---------- ---------- -----MRSFV YAGYEIDQER RQIYAFFGPG NETVWDFVGK
Z.mays(B73)(Chr9-CSD)              ---------- ---------- ---------- ---------- ---------- ---------- ----------
Z.mays(PH207)(Chr1-CSD)            ---------- ---------- ---------- ---------- ---------- ---------- ----------
Z.mays(PH207)(Chr7-CSD)            ---------- ---------- ---------- ---------- ---------- ---------- ----------
Z.mays(PH207)(Chr9-CSD)            ---------- ---------- ---------- ---------- ---------- ---------M KATRASRIEN
C.americanus(Chr5-CSD)             ---------- ---------- ---------- ---------- ---------- ---------- ----------
C.americanus(Chr7-CSD)             ---------- ---------- ---------- ---------- ---------- ---------- ----------
A.comosus(LG4-CSD)                 ---------- ---------- ---------- ---------- ---------- ---------- ----------
A.comosus(LG14-CSD)                ---------- ---------- ---------- ---------- ---------- ---------- ----------
M.acuminata(Chr8-CSD)              ---------- ---------- ---------- ---------- ---------- ---------- ----------
T.aestivum(Ch2A-CSD)               ---------- ---------- ---------- ---------- ---------- ---------- ----------
T.aestivum(Ch2D-CSD)               ---------- ---------- ---------- ---------- ---------- ---------- ----------
S.spontaneum(Chr1B-CSD)            ---------- ---------- ---------- ---------- ---------- ---------- ----------
S.spontaneum(Chr1C-CSD)            ---------- ---------- ---------- ---------- ---------- ---------- ----------
S.spontaneum(Chr1D-CSD)            ---------- ---------- ---------- ---------- ---------- ---------- ----------
S.spontaneum(Chr2C-CSD)            ---------- ---------- ---------- ---------- ---------- ---------- ----------
A.officinalis(V102-CSD)            ---------- ---------- ---------- ---------- ---------- ---------- ----------
A.officinalis(V108-CSD)            ---------- ---------- ---------- ---------- ---------- ---------- ----------
A.officinalis(V103-CSD)            ---------- ---------- ---------- ---------- ---------- ---------- ----------
Z.japonica(Sca0001-CSD)            ---------- ---------- ---------- ---------- ---------- ---------- ----------
Z.japonica(Sca0003.1-CSD)          ---------- ---------- ---------- ---------- ---------- ---------- ----------
H.vulgare(Chr2-CSD)                ---------- ---------- ---------- ---------- ---------- ---------- ----------
H.vulgare(Chr5-CSD)                ---------- ---------- ---------- ---------- ---------- ---------- ----------
H.vulgare(Chr5-CSDb)               ---------- ---------- ---------- ---------- ---------- ---------- ----------
C.sim(Sca247-CSD)                  ---------- ---------- ---------- ---------- ---------- ---------- ----------
C.sim(Sca212-CSD)                  ---------- ---------- ---------- ---------- ---------- ---------- ----------
B.distachyon-CSD                   ---------- ---------- ---------- ---------- ---------- ---------- ----------
S.polyrhiza-CSD                    ---------- ---------- ---------- ---------- ---------- ---------- ----------
P.glaucum-CSD                      ---------- ---------- ---------- ---------- ---------- ---------- ----------
Z.marina-CSD                       ---------- ---------- ---------- ---------- ---------- ---------- ----------
Dicots
D.carota(Chr1-CSD)                 ---------- ---------- ---------- ---------- ---------- ---------- ----------
D.carota(Chr9-CSD)                 ---------- ---------- ---------- ---------- ---------- ---------- ----------
M.esculanta(Chr8-CSD)              ---------- ---------- ---------- ---------- ---------- ---------- ----------
M.esculanta(Chr9-CSD)              ---------- ---------- ---------- ---------- ---------- ---------- ----------
E.guttata(Sca7-CSD)                ---------- ---------- ---------- ---------- ---------- ---------- ----------
E.guttata(Sca14-CSD)               ---------- ---------- ---------- ---------- ---------- ---------- ----------
C.cajan(LG03-CSDa)                 ---------- ---------- ---------- ---------- ---------- ---------- ----------
C.cajan(LG03-CSDb)                 ---------- ---------- ---------- ---------- ---------- ---------- ----------
C.cajan(SC130189-CSD)              ---------- ---------- ---------- ---------- ---------- ---------- ----------
H.bra(NW018746327.1-CSD)           ---------- ---------- ---------- ---------- ---------- ---------- ----------
H.bra(NW018745806.1-CSD)           ---------- ---------- ---------- ---------- ---------- ---------- ----------
H.bra(NW018746407.1-CSD)           ---------- ---------- ---------- ---------- ---------- ---------- ----------
E.grandis(Chr2-CSD)                ---------- ---------- ---------- ---------- ---------- ---------- ----------
E.grandis(Chr8-CSD)                ---------- ---------- ---------- ---------- ---------- ---------- ----------
C.melo(Sca3-CSD)                   ---------- ---------- ---------- ---------- ---------- ---------- ----------
C.melo(Sca25-CSD)                  ---------- ---------- ---------- ---------- ---------- ---------- ----------
C.clementina(Sca5-CSDa)            ---------- ---------- ---------- ---------- -----MERTA SIKLVALLAV LFCFVNATKS
C.clementina(Sca5-CSDb)            ---------- ---------- ---------- ---------- ---------- ---------- ----------
B.oleracea(Chr5-CSD)               ---------- ---------- ---------- ---------- ---------- ---------- ----------
B.oleracea(Chr8-CSD)               ---------- ---------- --------MS TSRAYIQVHK DKPATTKQLL PPDYSLPRHF ILFTSKGITK
A.chinensis(Chr3-CSD)              ---------- ---------- ---------- ---------- MPITRSTDQQ RLVSQIYATP TTINWTLDHS
A.chinensis(Chr29-CSD)             ---------- ---------- ---------- ---------- ---------- ---------- ----------
V.vinifera(Chr14-CSDa)             ---------- ---------- ---------- ---------- ---------- ---------- ----------
V.vinifera(Chr14-CSDb)             ---------- ---------- ---------- ---------- ---------- ---------- ----------
M.domestica(Bb4037-CSD)            ---------- ---------- ---------- ---------- ---------- ---------- ----------
M.domestica(Bb6170-CSD)            ---------- ---------- ---------- ---------- ---------- ---------- ----------
T.has(NW_010961290.1-CSD)          ---------- ---------- ---------- ---------- ---------- ---------- ----------
T.has(NW_010966881.1-CSD)          ---------- ---------- ---------- ---------- ---------- ---------- ----------
G.max(Chr3-CSD)                    ---------- ---------- ---------- ---------- ---------- ---------- ----------
G.max(Chr19-CSD)                   ---------- ---------- ---------- ---------- ---------- ---------- ----------
B.rapa(Chr6-CSD)                   ---------- ---------- ---------- ---------- ---------- ---------- ----------
B.rapa(Chr9-CSD)                   ---------- ---------- ---------- ---------- ---------- ---------- ----------
N.nucifera(MSc13-CSD)              ---------- ---------- ---------- ---------- ---------- ---------- ----------
N.nucifera(MSc9-CSD)               ---------- ---------- ---------- ---------- ---------- ---------- ----------
C.canephora(Chr2-CSD)              ---------- ---------- ---------- ---------- ---------- ---------- ----------
C.canephora(Chr3-CSD)              MSKRVNSEAK NAPQGAGVGE TSVLLIATAI AHAPHSLKIP LHFLHLFDTG TAVDLSLFIS FNNTSRGVLR
P.trichocarpa(Chr5-CSD)            ---------- ---------- ---------- ---------- ---------- ---------- ----------
P.trichocarpa(Chr13-CSD)           ---------- ---------- ---------- ---------- ---------- ---------- ----------
A.thaliana-CSD                     ---------- ---------- ---------- ---------- ---------- ---------- ----------
P.axilleris-CSD                    ---------- ---------- ---------- ---------- ---------- ---------- ----------
P.bretschneideri-CSD               ---------- ---------- ---------- ---------- ---------- ---------- ----------
S.lycopersicum-CSD                 ---------- ---------- ---------- ---------- ---------- ---------- ----------
M.truncatula-CSD                   ---------- ---------- ---------- ---------- ---------- ---------- ----------
V.radiata-CSD                      ---------- ---------- ---------- ---------- ---------- ---------- -------MVR
P.atrosanguinea-CSD                ---------- ---------- ---------- ---------- ---------- ---------- ----------
C.limon-CSD                        ---------- ---------- ---------- ---------- ---------- ---------- ----------
C.annuum-CSD                       ---------- ---------- ---------- ---------- ---------- ---------- ----------
C.aromatica-CSD                    ---------- ---------- ---------- ---------- ---------- ---------- ----------
A.trichopoda-CSD                   ---------- ---------- ---------- ---------- ---------- MVCGACVECL NFKLLAAFDE

                                   <------------------------------------ #1 ---------------------------------
```

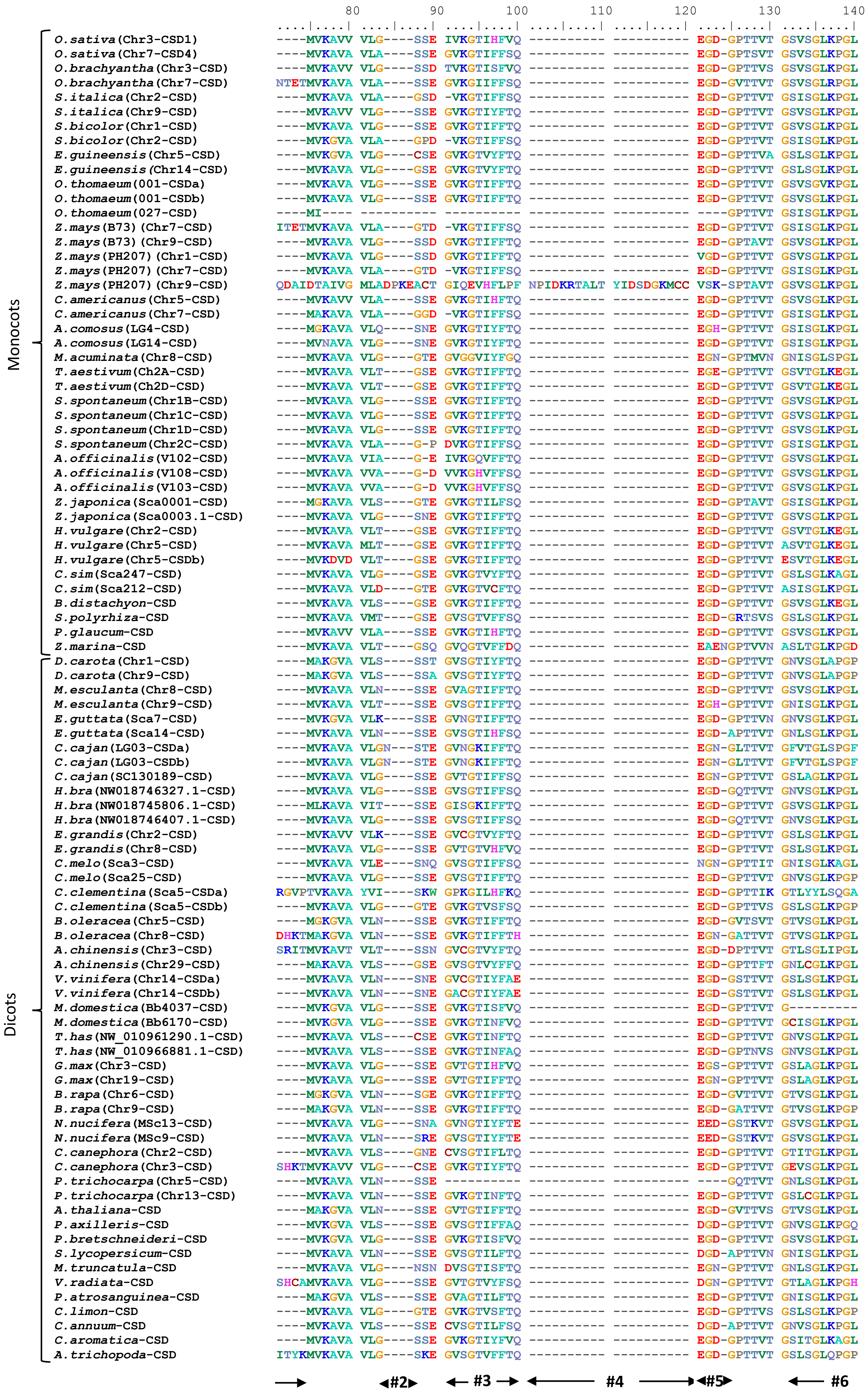
80 90 100 110 120 130 140
O.sativa(Chr3-CSD1) ----MVKAVV VLG----SSE IVKGTIHFVQ ---------- ---------- EGD-GPTTVT GSVSGLKPGL
O.sativa(Chr7-CSD4) ----MVKAVA VLA----SSE GVKGTIFFSQ ---------- ---------- EGD-GPTSVT GSVSGLKPGL
O.brachyantha(Chr3-CSD) ----MVKAVV VLG----SSD TVKGTISFVQ ---------- ---------- EGD-GPTTVS GSVSGLKPGL
O.brachyantha(Chr7-CSD) NTETMVKAVA VLA----SSE GVKGIIFFSQ ---------- ---------- EGD-GVTTVT GSVSGLRPGL
S.italica(Chr2-CSD) ----MVKAVA VLA----GSD -VKGTIFFSQ ---------- ---------- EGD-GPTTVT GSISGLKPGL
S.italica(Chr9-CSD) ----MVKAVV VLG----SSE GVKGTIYFTQ ---------- ---------- EGD-GPTTVT GSVSGLKPGL
S.bicolor(Chr1-CSD) ----MVKAVA VLG----SSE GVKGTIFFTQ ---------- ---------- EGD-GPTTVT GSVSGLKPGL
S.bicolor(Chr2-CSD) ----MVKGVA VLA----GPD -VKGTIFFSQ ---------- ---------- EGD-GPTTVT GSISGLKPGL
E.guineensis(Chr5-CSD) ----MVKGVA VLG----CSE GVKGTVYFTQ ---------- ---------- EGD-GPTTVA GSLSGLKPGL
E.guineensis(Chr14-CSD) ----MVKAVA VLG----GSE GVKGTVYFTQ ---------- ---------- EGD-GPTTVT GSLSGLKPGL
O.thomaeum(001-CSDa) ----MVKAVA VLG----SSE GVKGTIFFTQ ---------- ---------- EGD-GPTTVT GSVSGVKPGL
O.thomaeum(001-CSDb) ----MVKAVA VLG----SSE GVKGTIFFTQ ---------- ---------- EGD-GPTTVT GSVSGLKPGL
O.thomaeum(027-CSD) ----MI---- ---------- ---------- ---------- ---------- ----GPTTVT GSISGLKPGL
Z.mays(B73)(Chr7-CSD) ITETMVKAVA VLA----GTD -VKGTIFFSQ ---------- ---------- EGD-GPTTVT GSISGLKPGL
Z.mays(B73)(Chr9-CSD) ----MVKAVA VLG----SSD GVKGTIFFTQ ---------- ---------- EGD-GPTAVT GSVSGLKPGL
Z.mays(PH207)(Chr1-CSD) ----MVKAVA VLG----SSD GVKGTIFFTQ ---------- ---------- VGD-GPTTVT GSVSGLKPGL
Z.mays(PH207)(Chr7-CSD) ----MVKAVA VLA----GTD -VKGTIFFSQ ---------- ---------- EGD-GPTTVT GSISGLKPGL
Z.mays(PH207)(Chr9-CSD) QDAIDTAIVG MLADPKEACT GIQEVHFLPF NPIDKRTALT YIDSDGKMCC VSK-SPTAVT GSVSGLKPGL
C.americanus(Chr5-CSD) ----MVKAVV VLA----SSE GVKGTIHFTQ ---------- ---------- EGD-GPTTVT GSVSGLKPGL
C.americanus(Chr7-CSD) ----MAKAVA VLA----GGD -VKGTIFFSQ ---------- ---------- EGD-GPTTVT GSISGLKPGL
A.comosus(LG4-CSD) ----MGKAVA VLQ----SNE GVKGTIYFTQ ---------- ---------- EGH-GPTTVT GSISGLKPGL
A.comosus(LG14-CSD) ----MVNAVA VLG----SNE GVKGTIYFTQ ---------- ---------- EGD-GPTTVT GSISGLKPGL
M.acuminata(Chr8-CSD) ----MVKAVA VLG----GTE GVGGVIYFGQ ---------- ---------- EGN-GPTMVN GNISGLSPGL
T.aestivum(Ch2A-CSD) ----MVKAVA VLT----GSE GVKGTIFFTQ ---------- ---------- EGE-GPTTVT GSVTGLKEGL
T.aestivum(Ch2D-CSD) ----MVKAVA VLT----GSE GVKGTIFFTQ ---------- ---------- EGD-GPTTVT GSVTGLKEGL
S.spontaneum(Chr1B-CSD) ----MVKAVA VLG----SSE GVKGTIFFTQ ---------- ---------- EGD-GPTTVT GSVSGLKPGL
S.spontaneum(Chr1C-CSD) ----MVKAVA VLG----SSE GVKGTIFFTQ ---------- ---------- EGD-GPTTVT GSVSGLKPGL
S.spontaneum(Chr1D-CSD) ----MVKAVA VLG----SSE GVKGTIFFTQ ---------- ---------- EGD-GPTTVT GSVSGLKPGL
S.spontaneum(Chr2C-CSD) ----MVKAVA VLA----G-P DVKGTIFFSQ ---------- ---------- EGD-GPTTVT GSISGLKPGL
A.officinalis(V102-CSD) ----MVKAVA VIA----G-E IVKGQVFFTQ ---------- ---------- EGD-GPTTVT GSVSGLKPGL
A.officinalis(V108-CSD) ----MVKAVA VVA----G-D VVKGHVFFSQ ---------- ---------- EGD-GPTTVT GSVSGLKPGL
A.officinalis(V103-CSD) ----MVKAVA VVA----G-D VVKGHVFFSQ ---------- ---------- EGD-GPTTVT GSVSGLKPGL
Z.japonica(Sca0001-CSD) ----MGKAVA VLS----GTE GVKGTILFSQ ---------- ---------- EGD-GPTAVT GSISGLKPGL
Z.japonica(Sca0003.1-CSD) ----MVKAVA VLG----SNE GVKGTIFFTQ ---------- ---------- EGD-GPTTVT GSVSGLKPGL
H.vulgare(Chr2-CSD) ----MVKAVA VLT----GSE GVKGTIFFTQ ---------- ---------- EGD-GPTTVT GSVTGLKEGL
H.vulgare(Chr5-CSD) ----MVKAVA MLT----GSE GVKGTIFFTQ ---------- ---------- EGD-GPTTVT ASVTGLKEGL
H.vulgare(Chr5-CSDb) ----MVKDVD VLT----GSE GVKGTIFFTQ ---------- ---------- EGD-GPTTVT ESVTGLKEGL
C.sim(Sca247-CSD) ----MVKAVA VLG----GSE GVKGTVYFTQ ---------- ---------- EGD-GPTTVT GSLSGLKAGL
C.sim(Sca212-CSD) ----MVKAVA VLD----GTE GVKGTVCFTQ ---------- ---------- EGD-GPTTVT ASISGLKPGL
B.distachyon-CSD ----MVKAVA VLS----GSE GVKGTIFFTQ ---------- ---------- EGD-GPTTVT GSVSGLKEGL
S.polyrhiza-CSD ----MVKAVA VMT----GSE GVSGTVFFSQ ---------- ---------- EGD-GRTSVS GSLSGLKPGL
P.glaucum-CSD ----MVKAVV VLA----SSE GVKGTIHFTQ ---------- ---------- EGD-GPTTVT GSVSGLKPGL
Z.marina-CSD ----MVKAVA VLT----GSQ GVQGTVFFDQ ---------- ---------- EAENGPTVVN ASLTGLKPGD
D.carota(Chr1-CSD) ----MAKGVA VLS----SST GVSGTIYFTQ ---------- ---------- EGD-GPTTVT GNVSGLAPGP
D.carota(Chr9-CSD) ----MAKGVA VLS----SSA GVSGTIYFTQ ---------- ---------- EGD-GPTTVT GNVSGLAPGP
M.esculanta(Chr8-CSD) ----MVKAVA VLN----SSE GVAGTIFFTQ ---------- ---------- EGD-GPTTVT GSVSGLKPGL
M.esculanta(Chr9-CSD) ----MVKAVA VLT----SSE GVSGTIFFTQ ---------- ---------- EGH-GPTTVT GNISGLKPGL
E.guttata(Sca7-CSD) ----MVKGVA VLK----SSE GVNGTIFFTQ ---------- ---------- EGD-GPTTVN GNVSGLKPGL
E.guttata(Sca14-CSD) ----MVKAVA VLN----SSE GVSGTIHFSQ ---------- ---------- EGD-APTTVT GNLSGLKPGL
C.cajan(LG03-CSDa) ----MVKAVA VLGN---STE GVNGKIFFTQ ---------- ---------- EGN-GLTTVT GFVTGLSPGF
C.cajan(LG03-CSDb) ----MVKAVA VLGN---STE GVNGKIFFTQ ---------- ---------- EGN-GLTTVT GFVTGLSPGF
C.cajan(SC130189-CSD) ----MVKAVA VLG----SSE GVTGTIFFSQ ---------- ---------- EGN-GPTTVT GSLAGLKPGL
H.bra(NW018746327.1-CSD) ----MVKAVA VLG----SSE GVSGTIFFSQ ---------- ---------- EGD-GQTTVT GNVSGLKPGL
H.bra(NW018745806.1-CSD) ----MLKAVA VIT----SSE GISGKIFFTQ ---------- ---------- EGD-GPTTVT GSVSGLKPGL
H.bra(NW018746407.1-CSD) ----MVKAVA VLG----SSE GVSGTIFFSQ ---------- ---------- EGD-GQTTVT GNVSGLKPGL
E.grandis(Chr2-CSD) ----MVKAVV VLK----SSE GVCGTVYFTQ ---------- ---------- EGD-GPTTVT GSLSGLKPGL
E.grandis(Chr8-CSD) ----MVKAVA VLG----SSE GVTGTVHFVQ ---------- ---------- EGD-GPTTVT GSLSGLKPGL
C.melo(Sca3-CSD) ----MVKAVA VLE----SNQ GVSGTIFFSQ ---------- ---------- NGN-GPTTIT GNISGLKAGL
C.melo(Sca25-CSD) ----MVKAVA VLG----SSE GVSGTIFFTQ ---------- ---------- EGD-GPTTVT GNVSGLKPGP
C.clementina(Sca5-CSDa) RGVPTVKAVA YVI----SKW GPKGILHFKQ ---------- ---------- EGD-GPTTIK GTLYYLSQGA
C.clementina(Sca5-CSDb) ----MVKAVA VLG----GTE GVKGTVSFSQ ---------- ---------- EGD-GPTTVS GSLSGLKPGP
B.oleracea(Chr5-CSD) ----MGKGVA VLN----SSE GVKGTIFFTQ ---------- ---------- EGD-GVTSVT GTVSGLKPGL
B.oleracea(Chr8-CSD) DHKTMAKGVA VLN----SSE GVKGTIFFTH ---------- ---------- EGN-GATTVT GTVSGLKPGL
A.chinensis(Chr3-CSD) SRITMVKAVT VLT----SSN GVCGTVYFTQ ---------- ---------- EGD-DPTTVT GTLSGLIPGL
A.chinensis(Chr29-CSD) ----MAKAVA VLS----GSE GVSGTVYFFQ ---------- ---------- EGD-GPTTFT GNLCGLKPGL
V.vinifera(Chr14-CSDa) ----MVKAVA VLN----SNE GVCGTIYFAE ---------- ---------- EGD-GSTTVT GSLSGLKPGL
V.vinifera(Chr14-CSDb) ----MVKAVA VLN----SNE GACGTIYFAE ---------- ---------- EGD-GSTTVT GSLSGLKPGL
M.domestica(Bb4037-CSD) ----MVKGVA VLG----SSE GVKGTISFVQ ---------- ---------- EGD-GPTTVT G---------
M.domestica(Bb6170-CSD) ----MVKGVA VLG----SSE GVKGTINFVQ ---------- ---------- EGD-GPTTVT GCISGLKPGL
T.has(NW_010961290.1-CSD) ----MVKAVA VLS----CSE GVKGTINFTQ ---------- ---------- EGD-GPTTVT GNVSGLKPGL
T.has(NW_010966881.1-CSD) ----MVKAVA VLS----SSE GVKGTINFAQ ---------- ---------- EGD-GPTNVS GNVSGLKPGL
G.max(Chr3-CSD) ----MVKAVA VLG----SSE GVTGTIHFVQ ---------- ---------- EGS-GPTTVT GSLAGLKPGL
G.max(Chr19-CSD) ----MVKAVA VLG----SSE GVTGTIFFTQ ---------- ---------- EGN-GPTTVT GSLAGLKPGL
B.rapa(Chr6-CSD) ----MGKGVA VLN----SGE GVKGTIFFTQ ---------- ---------- EGD-GVTTVT GTVSGLKPGL
B.rapa(Chr9-CSD) ----MAKGVA VLN----SSE GVKGTIFFTQ ---------- ---------- EGD-GATTVT GTVSGLKPGP
N.nucifera(MSc13-CSD) ----MVKAVA VLG----SNA GVNGTIYFTE ---------- ---------- EED-GSTKVT GSVSGLKPGL
N.nucifera(MSc9-CSD) ----MVKAVA VLN----SRE GVSGTIYFTE ---------- ---------- EED-GSTKVT GSVSGLKPGL
C.canephora(Chr2-CSD) ----MVKAVA VLS----GNE CVSGTIFLTQ ---------- ---------- EGD-GPTTVT GTITGLKPGL
C.canephora(Chr3-CSD) SHKTMVKAVV VLG----CSE GVKGTIYFTQ ---------- ---------- EGD-GPTTVT GEVSGLKPGL
P.trichocarpa(Chr5-CSD) ----MVKAVA VLN----SSE ---------- ---------- ---------- ----GQTTVT GNLSGLKPGL
P.trichocarpa(Chr13-CSD) ----MVKAVA VLN----SSE GVKGTINFTQ ---------- ---------- EGD-GPTTVT GSLCGLKPGL
A.thaliana-CSD ----MAKGVA VLN----SSE GVTGTIFFTQ ---------- ---------- EGD-GVTTVS GTVSGLKPGL
P.axilleris-CSD ----MVKGVA VLS----SSE GVSGTIFFAQ ---------- ---------- DGD-GPTTVT GNVSGLKPGQ
P.bretschneideri-CSD ----MVKGVA VLG----SSE GVKGTISFVQ ---------- ---------- EGD-GPTTVT GSVSGLKPGL
S.lycopersicum-CSD ----MVKAVA VLN----SSE GVSGTILFTQ ---------- ---------- DGD-APTTVN GNISGLKPGL
M.truncatula-CSD ----MVKAVA VLG----NSN DVSGTISFTQ ---------- ---------- EGN-GPTTVT GNLSGLKPGL
V.radiata-CSD SHCAMVKAVA VLG----SSE GVTGTVYFSQ ---------- ---------- DGN-GPTTVT GTLAGLKPGH
P.atrosanguinea-CSD ----MAKGVA VLS----SSE GVAGTILFTQ ---------- ---------- EGD-GPTTVT GNISGLKPGL
C.limon-CSD ----MVKAVA VLG----GTE GVKGTVSFTQ ---------- ---------- EGD-GPTTVS GSLSGLKPGP
C.annuum-CSD ----MVKAVA VLS----SSE CVSGTILFSQ ---------- ---------- DGD-APTTVT GNVSGLKPGL
C.aromatica-CSD ----MVKAVA VLG----SSE GVKGTIYFVQ ---------- ---------- EGD-GPTTVT GSITGLKAGL
A.trichopoda-CSD ITYKMVKAVA VLG----SKE GVSGTVFFTQ ---------- ---------- EGD-GPTTVT GSLSGLQPGP
Monocots
Dicots
#2 #3 #4 #5 #6

```
                                    150        160        170        180        190        200        210
                              ....|....| ....|....| ....|....| ....|....| ....|....| ....|....| ....|....|
O.sativa(Chr3-CSD1)           HGFHIHALGD TTNGCMS--- ---TGPHYNP AGKEHGAPED ETRHAGDLGN VTAGEDG--- ----------
O.sativa(Chr7-CSD4)           HGFHVHALGD TTNGCMS--- ---TGPHFNP TGKEHGAPQD ENRHAGDLGN ITAGADG--- ----------
O.brachyantha(Chr3-CSD)       HGFHIHALGD TTNGCMS--- ---TGPHYNP AGKEHGAPED ETRHAGDLGN VTAGEDG--- ----------
O.brachyantha(Chr7-CSD)       HGFHVHALGD TTNGCMS--- ---TG-EMDY GVLITGEIAI CSYCR---AT LQSYWEG--- ----------
S.italica(Chr2-CSD)           HGFHVHALGD TTNGCMS--- ---TGPHFNP AGKEHGAPED ENRHAGDLGN VTAGEDG--- ----------
S.italica(Chr9-CSD)           HGFHVHALGD TTNGCMS--- ---TGPHYNP AGKEHGAPED DNRHAGDLGN VTAGADG--- ----------
S.bicolor(Chr1-CSD)           HGFHVHALGD TTNGCMS--- ---TGPHYNP ASKEHGAPED ENRHAGDLGN VTAGADG--- ----------
S.bicolor(Chr2-CSD)           HGFHVHALGD TTNGCMS--- ---TGPHFNP AGKEHGAPED ENRHAGDLGN VTAGEDG--- ----------
E.guineensis(Chr5-CSD)        HGFHVHALGD TTNGCMS--- ---TGPHFNP AGKEHGAPED DNRHAGDLGN VTVGDDG--- ----------
E.guineensis(Chr14-CSD)       HGFHVHALGD TTNGCMS--- ---TGPHFNP AGHEHGAPED EIRHAGDLGN VTAGDDG--- ----------
O.thomaeum(001-CSDa)          HGFHVHAFGD TTNGCMS--- ---TGPHYNP SGKEHGAPED EIRHAGDLGN VTAGPDG--- ----------
O.thomaeum(001-CSDb)          HGFHVHALGD TTNGCMS--- ---TGPHYNP AGKEHGAPED EIRHAGDFGN VTAGPDG--- ----------
O.thomaeum(027-CSD)           HGFHVHALGD TTNGCMS--- ---TGPHFNP TGKEHGAPED DNRHAGDLGN VTAGQDG--- ----------
Z.mays(B73)(Chr7-CSD)         HGFHVHALGD TTNGCMS--- ---TGPHFNP VGKEHGAPED EDRHAGDLGN VTAGEDG--- ----------
Z.mays(B73)(Chr9-CSD)         HGFHVHALGD TTNGCMS--- ---TGPHYNP ASKEHGAPED ENRHAGDLGN VTAGADG--- ----------
Z.mays(PH207)(Chr1-CSD)       HGFHVHALGD TTNGCMS--- ---TGPHYNP ASKEHGAPED ENRHAGDLGN VTAGADG--- ----------
Z.mays(PH207)(Chr7-CSD)       HGFHVHALGD TTNGCMS--- ---TGPHFNP VGKEHGAPED EDRHAGDLGN VTAGEDG--- ----------
Z.mays(PH207)(Chr9-CSD)       HGFHVHALGD TTNGCMS--- ---TGPHYNP ASKEHGAPED ENRHAGDLGN VTAGADG--- ----------
C.americanus(Chr5-CSD)        HGFHVHALGD TTNGCMS--- ---TGPHYNP AGKEHGAPED ENRHAGDLGN VTAGADGKDL LLIVPICCIP
C.americanus(Chr7-CSD)        HGFHVHALGD TTNGCMS--- ---TGPHFNP AGKEHGAPED ENRHAGDLGN VTAGEDG--- ----------
A.comosus(LG4-CSD)            HGFHVHALGD TTNGCMS--- ---TGPHFNP TGKEHGAPED ENRHAGDLGN VTAGEDGS-- ----------
A.comosus(LG14-CSD)           HGFHVHALGD TTNGCMS--- ---TGPHFNP AGKEHGAPED EHRHAGDLGN VTAGEDG--- ----------
M.acuminata(Chr8-CSD)         HGFHVHEFGD TTNGCMS--- ---TGPHFNP TGEDHGDRED PVRHIGDLGN VIAGDDG--- ----------
T.aestivum(Ch2A-CSD)          HGFHVHALGD TTNGCMS--- ---TGPHFNP AGHVHGAPED EIRHAG---- ---------- ----------
T.aestivum(Ch2D-CSD)          HGFHVHALGD TTNGCMS--- ---TGPHFNP AGHVHGAPED EIRHAGDLGN VTAGADG--- ----------
S.spontaneum(Chr1B-CSD)       HGFHVHALGD TTNGCIS--- ---TGPHYNP ASKEHGAPED ENRHAGDLGN VTAGADG--- ----------
S.spontaneum(Chr1C-CSD)       HGFHVHALGD TTNGCIS--- ---TGPHYNP ASMEHGAPED ENRHAGDLGN VTAGADG--- ----------
S.spontaneum(Chr1D-CSD)       HGFHVHALGD TTNGCIS--- ---TGPHYNP ASKEHGAPED ENRHAGDLGN VTAGADG--- ----------
S.spontaneum(Chr2C-CSD)       HGFHVHALGD TTNGCMS--- ---TGPHFNP AGKEHGAPED ENRHAGDLGN VTAGEDG--- ----------
A.officinalis(V102-CSD)       HGFHVHALGD TTNGCMS--- ---TGPHFNP AGKEHGAPND ENRHAGDLGN VTATEDG--- ----------
A.officinalis(V108-CSD)       HGFHVHALGD TTNGCMS--- ---TGPHFNP AGKEHGAPED ENRHAGDLGN VTVAEDG--- ----------
A.officinalis(V103-CSD)       HGFHVHALGD TTNGCMS--- ---TGPHFNP AGKEHGAPED ENRHAGDLGN VTVAEDG--- ----------
Z.japonica(Sca0001-CSD)       HGFHVHALGD TTNGCNS--- ---TGPHFNP TGKEHGAPED ENRHAGDLGN VTAGQDG--- ----------
Z.japonica(Sca0003.1-CSD)     HGFHVHALGD TTNGCMS--- ---TGPHYNP AGKEHGAPED EIRHAGDLGN VTAGADG--- ----------
H.vulgare(Chr2-CSD)           HGFHVHALGD TTNGCMS--- ---TGPHFNP AGHVHGAPED EIRHAGDLGN VTAGADG--- ----------
H.vulgare(Chr5-CSD)           HGFHVHALGD TTNGCMS--- ---TGPHFNP TGHVHGAPED EIRHAGDLGN VTAGADG--- ----------
H.vulgare(Chr5-CSDb)          HGFHVHALGD TTNGCMS--- ---TGPHFNP AGHVHGAPED EIRHAGDLGN VTAGADG--- ----------
C.sim(Sca247-CSD)             HGFHVHALGD TTNGCMS--- ---TGPHFNP AGKEHGAPED ENRHAGDLGN VTVGDDG--- ----------
C.sim(Sca212-CSD)             HGFHVHALGD TTNGCMS--- ---TGPHFNP AGKEHGAPED ENRHAGDLGN VIAGDDG--- ----------
B.distachyon-CSD              HGFHVHALGD TTNGCMS--- ---TGPHFNP AGKEHGAPED ETRHAGDLGN VTAGVDG--- ----------
S.polyrhiza-CSD               HGFHVHSLGD TTNGCMS--- ---TGPHFNP SGKEHGAPED EIRHAGDLGN VTAGEDG--- ----------
P.glaucum-CSD                 HGFHVHALGD TTNGCMS--- ---TGPHYNP AGKEHGAPED ENRHAGDLGN VTAGADG--- ----------
Z.marina-CSD                  HGFHIHALGD TTNGCMS--- ---TGPHFNP HGKVHGSPLD EIRHAGDLGN VTAKEDG--- ----------
D.carota(Chr1-CSD)            HGFHVHALGD TTNGCMS--- ---TGPHYNP AGKEHGAPED ENRHAGDLGN VTVGEDG--- ----------
D.carota(Chr9-CSD)            HGFHVHALGD TTNGCMS--- ---TGPHYNP AGKEHGAPED ENRHAGDLGN VTVGEDG--- ----------
M.esculanta(Chr8-CSD)         HGFHVHALGD TTNGCMS--- ---TGPHFNP GGKEHGAPED DIRHAGDLGN VTAGDDG--- ----------
M.esculanta(Chr9-CSD)         HGFHVHALGD TTNGCMS--- ---TGPHFNP SGKDHGAPED EIRHAGDLGN VTAGDDG--- ----------
E.guttata(Sca7-CSD)           HGFHVHALGD TTNGCLS--- ---TGPHFNP AGKEHGAPED EVRHAGDLGN VTVGEDG--- ----------
E.guttata(Sca14-CSD)          HGFHVHALGD TTNGCMS--- ---TGPHFNP AGKEHGAPDD EVRHAGDLGN VTVGEDG--- ----------
C.cajan(LG03-CSDa)            HGFHIHALGD TTNGCLS--- ---TGEHFDP IGKVHGGPTD VNRHAGDLGN LDVAGDG--- ----------
C.cajan(LG03-CSDb)            HGFHIHALGD TTNGCLS--- ---TGEHFDP IGKVHGGPTD VNRHAGDLGN LDVAGDG--- ----------
C.cajan(SC130189-CSD)         HGFHVHALGD TTNGCLS--- ---TGAHFNP NGKEHGAPED DNRHAGDLGN VNVGDDG--- ----------
H.bra(NW018746327.1-CSD)      HGFHVHALGD TTNGCMS--- ---TGPHFNP ASKEHGAPED ENRHAGDLGN INVGDDG--- ----------
H.bra(NW018745806.1-CSD)      HGFHVHTFGD TTNGCLSTGI VNWQGLHFNP ASKDHGGPED ENRHAGDLGN VNVGDDG--- ----------
H.bra(NW018746407.1-CSD)      HGFHVHALGD TTNGCMS--- ---TGPHFNP ASKEHGAPED ENRHAGDLGN INVGDDG--- ----------
E.grandis(Chr2-CSD)           HGFHVHALGD TTNGCMS--- ---TGPHFNP CGKEHGAPED QNRHAGDLGN VNVGDDG--- ----------
E.grandis(Chr8-CSD)           HGFHVHALGD TTNGCMS--- ---TGPHFNP VGKEHGAPED ENRHAGDLGN VTVGDNG--- ----------
C.melo(Sca3-CSD)              HGFHVHALGD TTNGCLS--- ---TGPHFNP EGKDHGAPDD ENRHVGDLGN VVAGDNG--- ----------
C.melo(Sca25-CSD)             HGFHVHALGD TTNGCMS--- ---TGPHFNP AGKQHGAPED ENRHAGDLGN IIVGEDG--- ----------
C.clementina(Sca5-CSDa)       HGFHIHVYGD MAHFCQS--- ---TGDHFNP FRKHHGGPED WIRHAGDLGN IYVGFDG--- ----------
C.clementina(Sca5-CSDb)       HGFHVHALGD TTNGCMS--- ---TGPHFNP AGKEHGAPED ENRHAGDLGN VNVGDDG--- ----------
B.oleracea(Chr5-CSD)          HGFHVHALGD TTNGCMS--- ---TGPHFNP DGKQHGAPED ANRHAGDLGN IIVGDDG--- ----------
B.oleracea(Chr8-CSD)          HGFHVHALGD TTNGCMS--- ---TGPHFNP DGKTHGAPED ANRHAGDLGN IIVGDDG--- ----------
A.chinensis(Chr3-CSD)         HGFHVHALGD TTNGCMS--- ---TGPHFNP GSKEHGAPED ENRHAGDLGN ITVGEDG--- ----------
A.chinensis(Chr29-CSD)        HGFHVHALGD TTNGCMS--- ---TGPHYNP ASKEHGAPGD ENRHAGDLGN VEVGED---- ----------
V.vinifera(Chr14-CSDa)        HGFHVHALGD TTNGCMS--- ---TGPHFNP AGKEHGAPED ENRHAGDLGN VIVGEDG--- ----------
V.vinifera(Chr14-CSDb)        HGFHVHALGD TTNGCMS--- ---TGPHFNP AGKEHGAPED ENRHAGDLGN VIVGEDG--- ----------
M.domestica(Bb4037-CSD)       ---------- ---------- ----RPHFNP AGKEHGAPED EVRHAGDLGN ITAGDDG--- ----------
M.domestica(Bb6170-CSD)       HGFHVHAFGD TTNGCLSTGK GPLTGPHFNP NGKEHGAPE- EDRHAGDLGN VTVGDDG--- ----------
T.has(NW_010961290.1-CSD)     HGFHVHALGD TTNGCMS--- ---TGPHFNP AGKEHGAPDD GNRHAGDLGN ITVGDDG--- ----------
T.has(NW_010966881.1-CSD)     HGFHVHALGD TTNGCMS--- ---TGPHFNP AGKEHGAPED EHRHAGDLGN ITVGADG--- ----------
G.max(Chr3-CSD)               HGFHVHALGD TTNGCLS--- ---TGSHFNP NNKEHGAPED ENRHAGDLGN VNVGDDG--- ----------
G.max(Chr19-CSD)              HGFHVHALGD TTNGCLS--- ---TGAHFNP NNNEHGAPED ENRHAGDLGN VNVGDDG--- ----------
B.rapa(Chr6-CSD)              HGFHVHALGD TTNGCMS--- ---TGPHFNP DGKQHGAPED ANRHAGDLGN IIVGDDG--- ----------
B.rapa(Chr9-CSD)              HGFHVHALGD TTNGCMS--- ---TGPHFNP DGKTHGAPED ANRHAGDLGN IIVGDDG--- ----------
N.nucifera(MSc13-CSD)         HGFHVHALGD TTNGCMS--- ---TGPHFNP HGKEHGAPED ENRHAGDLGN VTVGEDG--- ----------
N.nucifera(MSc9-CSD)          HGFHVHALGD TTNGCMS--- ---TGPHFNP QSKEHGAPED ENRHAGDLGN VTVGEDGRCS SVFSLSCMAI
C.canephora(Chr2-CSD)         HGFHVHALGD TTNGCMS--- ---TGPHFNP KGKEHGAPCD ENRHAGDLGN VTAGQDG--- ----------
C.canephora(Chr3-CSD)         HGFHVHALGD TTNGCLS--- ---TGPHFNP GGKEHGAPED ENRHAGDLGN ITVGEDG--- ----------
P.trichocarpa(Chr5-CSD)       HGFHVHALGD TTNGCMS--- ---TGPHFNP VGKEHGAPED ENRHAGDLGN VTVGDDG--- ----------
P.trichocarpa(Chr13-CSD)      HGFHVHALGD TTNGCMS--- ---TGPHFNP VGKEHGAPED ENRHAGDLGN VTVGDDG--- ----------
A.thaliana-CSD                HGFHVHALGD TTNGCMS--- ---TGPHFNP DGKTHGAPED ANRHAGDLGN ITVGDDG--- ----------
P.axilleris-CSD               HGFHVHALGD TTNGCMS--- ---TGPHFNP AGKEHGSPED EVRHAGDLGN ITVGEDG--- ----------
P.bretschneideri-CSD          HGFHVHALGD TTNGCMS--- ---TGPHFNP AGKEHGAPED EDRHAGDLGN ITAGDDG--- ----------
S.lycopersicum-CSD            HGFHVHALGD TTNGCMS--- ---TGPHYNP AGKEHGAPED EVRHAGDLGN ITVGEDG--- ----------
M.truncatula-CSD              HGFHIHALGD TTNGCLS--- ---TGPHFNP NGKEHGAPED ETRHAGDLGN VTVGDDG--- ----------
V.radiata-CSD                 HGFHVHALGD TTNGCLS--- ---TGPHFNP NNKEHGAPED ENRHAGDLGN VNVGDDG--- ----------
P.atrosanguinea-CSD           HGFHVHALGD TTNGCMS--- ---TGPHFNP AGKEHGSPED ETRHAGDLGN ITVGDDG--- ----------
C.limon-CSD                   HGFHVHALGD TTNGCMS--- ---TGPHFNP AGKEHGAPED DNRHAGDLGN VNVSDDG--- ----------
C.annuum-CSD                  HGFHVHALGD TTNGCMS--- ---TGPHYNP AGKEHGAPED ENRHAGDLGN ITVGEDG--- ----------
C.aromatica-CSD               HGFHVHALGD TTNGCMS--- ---TGPHFNP AGKEHGAPED VNRHAGDLGN VTASEDG--- ----------
A.trichopoda-CSD              HGFHVHALGD TTNGCMS--- ---TGPHFNP VGKEHGAPED ENRHAGDLGN VTVGEDG--- ----------

                              ----------------> <- #7 ->                 <- #8 -> <----------------
```

Monocots (bracket: O.sativa(Chr3-CSD1) to Z.marina-CSD)

Dicots (bracket: D.carota(Chr1-CSD) to A.trichopoda-CSD)

```
                                220        230        240        250        260        270        280
                          ....|....| ....|....| ....|....| ....|....| ....|....| ....|....| ....|....|
Monocots
O.sativa(Chr3-CSD1)       ---------- ---------- ---------- ---------- ---------- --------VA NIHVVDS---
O.sativa(Chr7-CSD4)       ---------- ---------- ---------- ---------- ---------- --------VA NVNVSDS---
O.brachyantha(Chr3-CSD)   ---------- ---------- ---------- ---------- ---------- --------VA NINVVDS---
O.brachyantha(Chr7-CSD)   ---------- ---------- ---------- ---------- ---------- --------VA NVNISDS---
S.italica(Chr2-CSD)       ---------- ---------- ---------- ---------- ---------- --------VA NVNITDC---
S.italica(Chr9-CSD)       ---------- ---------- ---------- ---------- ---------- --------VA NIHVTDS---
S.bicolor(Chr1-CSD)       ---------- ---------- ---------- ---------- ---------- --------VA NISVTDS---
S.bicolor(Chr2-CSD)       ---------- ---------- ---------- ---------- ---------- --------VV NVNITDS---
E.guineensis(Chr5-CSD)    ---------- ---------- ---------- ---------- ---------- --------TV SFTITDN---
E.guineensis(Chr14-CSD)   ---------- ---------- ---------- ---------- ---------- --------TV NFTIIDS---
O.thomaeum(001-CSDa)      ---------- ---------- ---------- ---------- ---------- --------VA NINVTDC---
O.thomaeum(001-CSDb)      ---------- ---------- ---------- ---------- ---------- --------VA NINVTDC---
O.thomaeum(027-CSD)       ---------- ---------- ---------- ---------- ---------- --------VA NVNIKDS---
Z.mays(B73)(Chr7-CSD)     ---------- ---------- ---------- ---------- ---------- --------VV NVNITDS---
Z.mays(B73)(Chr9-CSD)     ---------- ---------- ---------- ---------- ---------- --------VA NINVTDS---
Z.mays(PH207)(Chr1-CSD)   ---------- ---------- ---------- ---------- ---------- --------VA NINVTDS---
Z.mays(PH207)(Chr7-CSD)   ---------- ---------- ---------- ---------- ---------- --------VV NVNITDS---
Z.mays(PH207)(Chr9-CSD)   ---------- ---------- ---------- ---------- ---------- --------VA NINVTDS---
C.americanus(Chr5-CSD)    MQWVLGTRLL ALWKLSYFCD PVDVACNSIH NLTCYVPMYE TIRHPTSLVI DFISLLSGVA NINVTDS---
C.americanus(Chr7-CSD)    ---------- ---------- ---------- ---------- ---------- --------VA NVNITDC---
A.comosus(LG4-CSD)        ---------- ---------- ---------- ---------- ---------- --------TA NVNIVDN---
A.comosus(LG14-CSD)       ---------- ---------- ---------- ---------- ---------- --------TA TFTIVDN---
M.acuminata(Chr8-CSD)     ---------- ---------- ---------- ---------- ---------- --------TA NFTMFDS---
T.aestivum(Ch2A-CSD)      ---------- ---------- ---------- ---------- ---------- --------VA SINITDC---
T.aestivum(Ch2D-CSD)      ---------- ---------- ---------- ---------- ---------- --------VA NINVTDC---
S.spontaneum(Chr1B-CSD)   ---------- ---------- ---------- ---------- ---------- --------VA NISVTDS---
S.spontaneum(Chr1C-CSD)   ---------- ---------- ---------- ---------- ---------- --------VA NISVTDSQVI
S.spontaneum(Chr1D-CSD)   ---------- ---------- ---------- ---------- ---------- --------VA NISVTDS---
S.spontaneum(Chr2C-CSD)   ---------- ---------- ---------- ---------- ---------- --------VV NVNITDS---
A.officinalis(V102-CSD)   ---------- ---------- ---------- ---------- ---------- --------TA SFSIVDS---
A.officinalis(V108-CSD)   ---------- ---------- ---------- ---------- ---------- --------TA TFSITDS---
A.officinalis(V103-CSD)   ---------- ---------- ---------- ---------- ---------- --------TA TFSITDS---
Z.japonica(Sca0001-CSD)   ---------- ---------- ---------- ---------- ---------- --------VA NVNITDS---
Z.japonica(Sca0003.1-CSD) ---------- ---------- ---------- ---------- ---------- --------VA NINVTDS---
H.vulgare(Chr2-CSD)       ---------- ---------- ---------- ---------- ---------- --------VA NINVTDC---
H.vulgare(Chr5-CSD)       ---------- ---------- ---------- ---------- ---------- --------VA NINVTDC---
H.vulgare(Chr5-CSDb)      ---------- ---------- ---------- ---------- ---------- --------VA NINVTDC---
C.sim(Sca247-CSD)         ---------- ---------- ---------- ---------- ---------- --------IV --FVSGC---
C.sim(Sca212-CSD)         ---------- ---------- ---------- ---------- ---------- --------TV NFTVVDN---
B.distachyon-CSD          ---------- ---------- ---------- ---------- ---------- --------VA NINVVDT---
S.polyrhiza-CSD           ---------- ---------- ---------- ---------- ---------- --------NV NITIVDS---
P.glaucum-CSD             ---------- ---------- ---------- ---------- ---------- --------VA NINVTDS---
Z.marina-CSD              ---------- ---------- ---------- ---------- ---------- --------TV NFTTTDT---
Dicots
D.carota(Chr1-CSD)        ---------- ---------- ---------- ---------- ---------- --------TA SFTIVDK---
D.carota(Chr9-CSD)        ---------- ---------- ---------- ---------- ---------- --------TA SFTIVDK---
M.esculanta(Chr8-CSD)     ---------- ---------- ---------- ---------- ---------- --------TA SFTIVDK---
M.esculanta(Chr9-CSD)     ---------- ---------- ---------- ---------- ---------- --------TA SFTIIDK---
E.guttata(Sca7-CSD)       ---------- ---------- ---------- ---------- ---------- --------TA AFTIIDE---
E.guttata(Sca14-CSD)      ---------- ---------- ---------- ---------- ---------- --------TA SFTITDS---
C.cajan(LG03-CSDa)        ---------- ---------- ---------- ---------- ---------- --------KV SFSITVS---
C.cajan(LG03-CSDb)        ---------- ---------- ---------- ---------- ---------- ---------M AYNFVFC---
C.cajan(SC130189-CSD)     ---------- ---------- ---------- ---------- ---------- --------KV NFSITDS---
H.bra(NW018746327.1-CSD)  ---------- ---------- ---------- ---------- ---------- --------TA SFTIVDK---
H.bra(NW018745806.1-CSD)  ---------- ---------- ---------- ---------- ---------- --------TA NFTIVDK---
H.bra(NW018746407.1-CSD)  ---------- ---------- ---------- ---------- ---------- --------TA SFTIVDK---
E.grandis(Chr2-CSD)       ---------- ---------- ---------- ---------- ---------- --------TV NFTIIDN---
E.grandis(Chr8-CSD)       ---------- ---------- ---------- ---------- ---------- --------TA TFTITDK---
C.melo(Sca3-CSD)          ---------- ---------- ---------- ---------- ---------- --------TA TFSIIDK---
C.melo(Sca25-CSD)         ---------- ---------- ---------- ---------- ---------- --------TA NFTITDN---
C.clementina(Sca5-CSDa)   ---------- ---------- ---------- ---------- ---------- --------KA KINIVDKHK-
C.clementina(Sca5-CSDb)   ---------- ---------- ---------- ---------- ---------- --------TA TFTVVDN---
B.oleracea(Chr5-CSD)      ---------- ---------- ---------- ---------- ---------- --------TA TFTITDC---
B.oleracea(Chr8-CSD)      ---------- ---------- ---------- ---------- ---------- --------TA TFTITDS---
A.chinensis(Chr3-CSD)     ---------- ---------- ---------- ---------- ---------- --------TA SFTIVDS---
A.chinensis(Chr29-CSD)    ---------- ---------- ---------- ---------- ---------- ---------- ----------
V.vinifera(Chr14-CSDa)    ---------- ---------- ---------- ---------- ---------- --------TV NFKI------
V.vinifera(Chr14-CSDb)    ---------- ---------- ---------- ---------- ---------- --------TV NFKIVDK---
M.domestica(Bb4037-CSD)   ---------- ---------- ---------- ---------- ---------- --------TA TFTIVDK---
M.domestica(Bb6170-CSD)   ---------- ---------- ---------- ---------- ---------- --------TA TFTLIDK---
T.has(NW_010961290.1-CSD) ---------- ---------- ---------- ---------- ---------- --------TA NFTIVDS---
T.has(NW_010966881.1-CSD) ---------- ---------- ---------- ---------- ---------- --------TA NFTIVDN---
G.max(Chr3-CSD)           ---------- ---------- ---------- ---------- ---------- --------TV SFTITDS---
G.max(Chr19-CSD)          ---------- ---------- ---------- ---------- ---------- --------TV SFSITDS---
B.rapa(Chr6-CSD)          ---------- ---------- ---------- ---------- ---------- --------TA TFTITDC---
B.rapa(Chr9-CSD)          ---------- ---------- ---------- ---------- ---------- --------TA TFTITDS---
N.nucifera(MSc13-CSD)     ---------- ---------- ---------- ---------- ---------- --------TV NFTIVDK---
N.nucifera(MSc9-CSD)      TVIIILQFFT AVTVLYRLHL CLKAWV-SYH ---------- ---------- ------SGTV NITIVDK---
C.canephora(Chr2-CSD)     ---------- ---------- ---------- ---------- ---------- --------TA NVLVVDN---
C.canephora(Chr3-CSD)     ---------- ---------- ---------- ---------- ---------- --------TA SFNIVDK---
P.trichocarpa(Chr5-CSD)   ---------- ---------- ---------- ---------- ---------- --------TA TFTIIDK---
P.trichocarpa(Chr13-CSD)  ---------- ---------- ---------- ---------- ---------- --------TA TVSIIDN---
A.thaliana-CSD            ---------- ---------- ---------- ---------- ---------- --------TA TFTITDC---
P.axilleris-CSD           ---------- ---------- ---------- ---------- ---------- --------TA SFTITDK---
P.bretschneideri-CSD      ---------- ---------- ---------- ---------- ---------- --------TV TFTIVDK---
S.lycopersicum-CSD        ---------- ---------- ---------- ---------- ---------- --------TA SFTITDK---
M.truncatula-CSD          ---------- ---------- ---------- ---------- ---------- --------TA SFTITDN---
V.radiata-CSD             ---------- ---------- ---------- ---------- ---------- --------TV TFSITDS---
P.atrosanguinea-CSD       ---------- ---------- ---------- ---------- ---------- --------TA CFTIVDK---
C.limon-CSD               ---------- ---------- ---------- ---------- ---------- --------TA TFTVVDN---
C.annuum-CSD              ---------- ---------- ---------- ---------- ---------- --------TA SFTITDE---
C.aromatica-CSD           ---------- ---------- ---------- ---------- ---------- --------IV AVSVVDK---
A.trichopoda-CSD          ---------- ---------- ---------- ---------- ---------- --------TA NFTIVDS---
```

#9

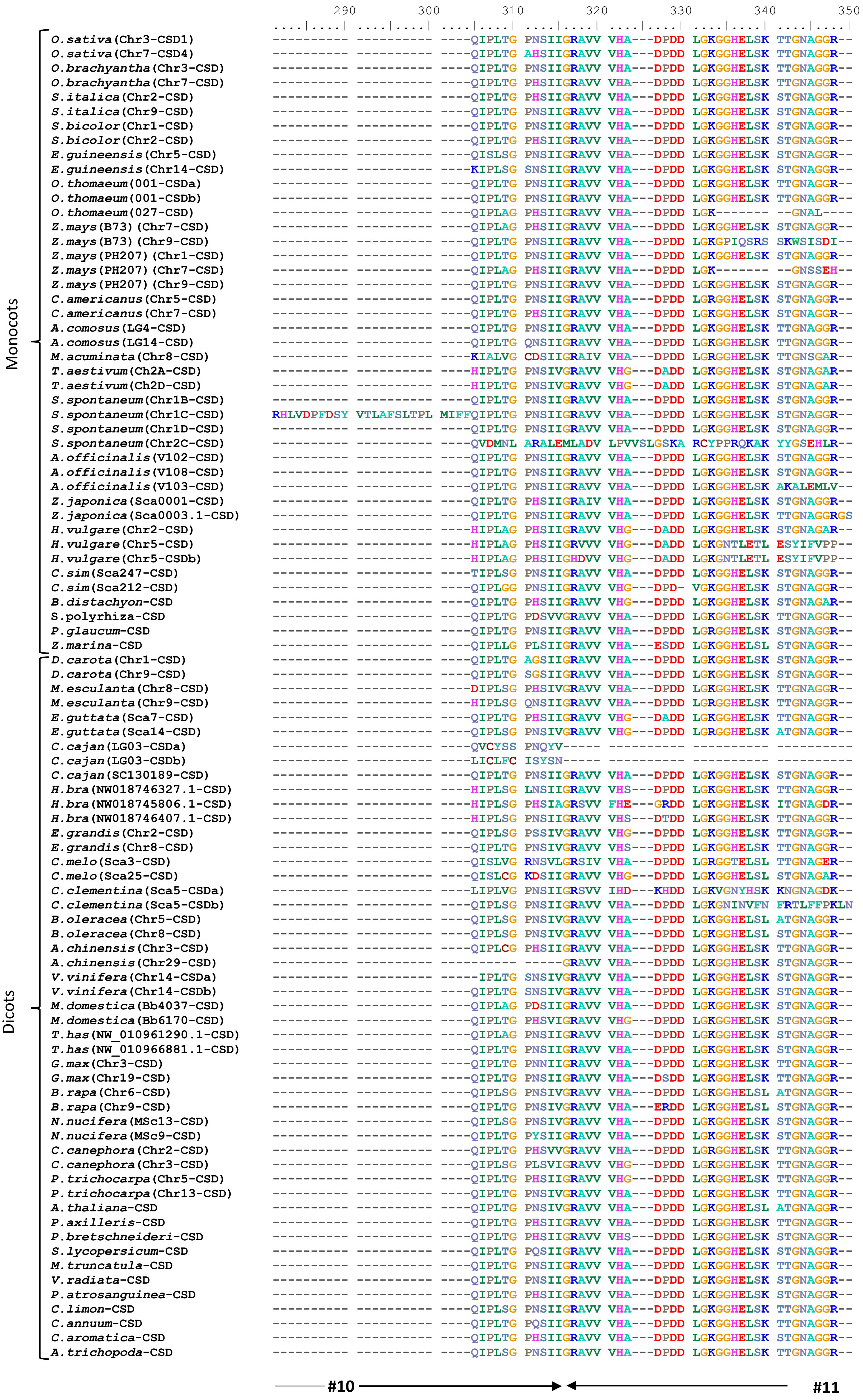

290 300 310 320 330 340 350
Monocots
O.sativa(Chr3-CSD1) ---------- ---------- ----QIPLTG PNSIIGRAVV VHA---DPDD LGKGGHELSK TTGNAGGR--
O.sativa(Chr7-CSD4) ---------- ---------- ----QIPLTG AHSIIGRAVV VHA---DPDD LGKGGHELSK TTGNAGGR--
O.brachyantha(Chr3-CSD) ---------- ---------- ----QIPLTG PNSIIGRAVV VHA---DPDD LGKGGHELSK TTGNAGGR--
O.brachyantha(Chr7-CSD) ---------- ---------- ----QIPLTG PHSIIGRAVV VHA---DPDD LGKGGHELSK TTGNAGGR--
S.italica(Chr2-CSD) ---------- ---------- ----QIPLTG PHSIIGRAVV VHA---DPDD LGKGGHELSK STGNAGGR--
S.italica(Chr9-CSD) ---------- ---------- ----QIPLTG PNSIIGRAVV VHA---DPDD LGKGGHELSK STGNAGGR--
S.bicolor(Chr1-CSD) ---------- ---------- ----QIPLTG PNSIIGRAVV VHA---DPDD LGKGGHELSK STGNAGGR--
S.bicolor(Chr2-CSD) ---------- ---------- ----QIPLTG PHSIIGRAVV VHA---DPDD LGKGGHELSK STGNAGGR--
E.guineensis(Chr5-CSD) ---------- ---------- ----QISLSG PNSIIGRAVV VHA---DPDD LGKGGHELSK STGNAGGR--
E.guineensis(Chr14-CSD) ---------- ---------- ----KIPLSG SNSIIGRAVV VHA---DPDD LGKGGHELSK STGNAGGR--
O.thomaeum(001-CSDa) ---------- ---------- ----QIPLTG PNSIIGRAVV VHA---DPDD LGKGGHELSK TTGNAGGR--
O.thomaeum(001-CSDb) ---------- ---------- ----QIPLTG PNSIIGRAVV VHA---DPDD LGKGGHELSK TTGNAGGR--
O.thomaeum(027-CSD) ---------- ---------- ----QIPLAG PHSIIGRAVV VHA---DPDD LGK------- --GNAL----
Z.mays(B73)(Chr7-CSD) ---------- ---------- ----QIPLAG PHSIIGRAVV VHA---DPDD LGKGGHELSK STGNAGGR--
Z.mays(B73)(Chr9-CSD) ---------- ---------- ----QIPLTG PNSIIGRAVV VHA---DPDD LGKGPIQSRS SKWSISDI--
Z.mays(PH207)(Chr1-CSD) ---------- ---------- ----QIPLTG PNSIIGRAVV VHA---DPDD LGKGGHELSK STGNAGGR--
Z.mays(PH207)(Chr7-CSD) ---------- ---------- ----QIPLAG PHSIIGRAVV VHA---DPDD LGK------- --GNSSEH--
Z.mays(PH207)(Chr9-CSD) ---------- ---------- ----QIPLTG PNSIIGRAVV VHA---DPDD LGKGGHELSK STGNAGGR--
C.americanus(Chr5-CSD) ---------- ---------- ----QIPLTG PNSIIGRAVV VHA---DPDD LGRGGHELSK STGNAGGR--
C.americanus(Chr7-CSD) ---------- ---------- ----QIPLTG PHSIIGRAVV VHA---DPDD LGKGGHELSK STGNAGGR--
A.comosus(LG4-CSD) ---------- ---------- ----QIPLTG PNSIIGRAVV VHA---DPDD LGKGGHELSK TTGNAGGR--
A.comosus(LG14-CSD) ---------- ---------- ----QIPLTG QNSIIGRAVV VHA---DPDD LGKGGHELSK TTGNAGGR--
M.acuminata(Chr8-CSD) ---------- ---------- ----KIALVG CDSIIGRAIV VHA---DPDD LGRGGHELSK TTGNSGAR--
T.aestivum(Ch2A-CSD) ---------- ---------- ----HIPLTG PNSIVGRAVV VHG---DADD LGKGGHELSK STGNAGAR--
T.aestivum(Ch2D-CSD) ---------- ---------- ----HIPLTG PNSIVGRAVV VHG---DADD LGKGGHELSK STGNAGAR--
S.spontaneum(Chr1B-CSD) ---------- ---------- ----QIPLTG PNSIIGRAVV VHA---DPDD LGKGGHELSK STGNAGGR--
S.spontaneum(Chr1C-CSD) RHLVDPFDSY VTLAFSLTPL MIFFQIPLTG PNSIIGRAVV VHA---DPDD LGKGGHELSK STGNAGGR--
S.spontaneum(Chr1D-CSD) ---------- ---------- ----QIPLTG PNSIIGRAVV VHA---DPDD LGKGGHELSK STGNAGGR--
S.spontaneum(Chr2C-CSD) ---------- ---------- ----QVDMNL ARALEMLADV LPVVSLGSKA RCYPPRQKAK YYGSEHLR--
A.officinalis(V102-CSD) ---------- ---------- ----QIPLTG PNSIIGRAVV VHA---DPDD LGKGGHELSK STGNAGGR--
A.officinalis(V108-CSD) ---------- ---------- ----QIPLTG PNSIIGRAVV VHA---DPDD LGKGGHELSK STGNAGGR--
A.officinalis(V103-CSD) ---------- ---------- ----QIPLTG PNSIIGRAVV VHA---DPDD LGKGGHELSK AKALEMLV--
Z.japonica(Sca0001-CSD) ---------- ---------- ----QIPLTG PHSIIGRAIV VHA---DPDD LGKGGHELSK STGNAGGR--
Z.japonica(Sca0003.1-CSD) ---------- ---------- ----QIPLTG PNSIIGRAVV VHA---DPDD LGKGGHELSK TTGNAGGRGS
H.vulgare(Chr2-CSD) ---------- ---------- ----HIPLAG PHSIIGRAVV VHG---DADD LGKGGHELSK STGNAGAR--
H.vulgare(Chr5-CSD) ---------- ---------- ----HIPLAG PHSIIGRVVV VHG---DADD LGKGNTLETL ESYIFVPP--
H.vulgare(Chr5-CSDb) ---------- ---------- ----HIPLAG PHSIIGHDVV VHG---DADD LGKGNTLETL ESYIFVPP--
C.sim(Sca247-CSD) ---------- ---------- ----TIPLSG PNSIIGRAVV VHA---DPDD LGKGGHELSK STGNAGGR--
C.sim(Sca212-CSD) ---------- ---------- ----QIPLGG PNSIIGRAVV VHG---DPD- VGKGGHELSK STGNAGGR--
B.distachyon-CSD ---------- ---------- ----QIPLTG PHSIIGRAVV VHG---DPDD LGKGGHELSK STGNAGAR--
S.polyrhiza-CSD ---------- ---------- ----QIPLTG PDSVVGRAVV VHA---DPDD LGKGGHELSK TTGNAGGR--
P.glaucum-CSD ---------- ---------- ----QIPLTG PNSIIGRAVV VHA---DPDD LGRGGHELSK STGNAGGR--
Z.marina-CSD ---------- ---------- ----QIPLLG PLSIIGRAVV VHA---ESDD LGKGGHELSL STGNAGGR--
Dicots
D.carota(Chr1-CSD) ---------- ---------- ----QIPLTG AGSIIGRAVV VHA---DPDD LGKGGHELSK STGNAGGR--
D.carota(Chr9-CSD) ---------- ---------- ----QIPLTG SGSIIGRAVV VHA---DPDD LGKGGHELSK STGNAGGR--
M.esculanta(Chr8-CSD) ---------- ---------- ----DIPLSG PHSIVGRAVV VHA---DPDD LGKGGHELSK TTGNAGGR--
M.esculanta(Chr9-CSD) ---------- ---------- ----HIPLSG QNSIIGRAVV VHA---DPDD LGRGGHELSK TTGNAGGR--
E.guttata(Sca7-CSD) ---------- ---------- ----QIPLTG PHSIIGRAVV VHG---DADD LGKGGHELSK TTGNAGGR--
E.guttata(Sca14-CSD) ---------- ---------- ----QIPLSG PNSIVGRAVV VHG---DPDD LGRGGHELSK ATGNAGGR--
C.cajan(LG03-CSDa) ---------- ---------- ----QVCYSS PNQYV----- ---------- ---------- ----------
C.cajan(LG03-CSDb) ---------- ---------- ----LICLFC ISYSN----- ---------- ---------- ----------
C.cajan(SC130189-CSD) ---------- ---------- ----QIPLTG PNSIIGRAVV VHA---DPDD LGKGGHELSK STGNAGGR--
H.bra(NW018746327.1-CSD) ---------- ---------- ----HIPLSG LNSIIGRAVV VHS---DPDD LGKGGHELSK TTGNAGGR--
H.bra(NW018745806.1-CSD) ---------- ---------- ----HIPLSG PHSIAGRSVV FHE---GRDD LGKGGHELSK ITGNAGDR--
H.bra(NW018746407.1-CSD) ---------- ---------- ----HIPLSG PNSIIGRAVV VHS---DTDD LGKGGHELSK TTGNAGGR--
E.grandis(Chr2-CSD) ---------- ---------- ----QIPLSG PSSIVGRAVV VHG---DPDD LGKGGHELSK TTGNAGGR--
E.grandis(Chr8-CSD) ---------- ---------- ----QIPLTG PNSIVGRAVV VHS---DPDD LGKGGHELSK TTGNAGGR--
C.melo(Sca3-CSD) ---------- ---------- ----QISLVG RNSVLGRSIV VHA---DPDD LGRGGTELSL TTGNAGER--
C.melo(Sca25-CSD) ---------- ---------- ----QISLCG KDSIIGRAVV VHG---DPDD LGKGGHELSL STGNAGAR--
C.clementina(Sca5-CSDa) ---------- ---------- ----LIPLVG PNSIIGRSVV IHD---KHDD LGKVGNYHSK KNGNAGDK--
C.clementina(Sca5-CSDb) ---------- ---------- ----QIPLSG PNSIIGRAVV VHA---DPDD LGKGNINVFN FRTLFFPKLN
B.oleracea(Chr5-CSD) ---------- ---------- ----QIPLSG PNSIVGRAVV VHA---DPDD LGKGGHELSL ATGNAGGR--
B.oleracea(Chr8-CSD) ---------- ---------- ----QIPLSG PNSIVGRAVV VHA---DPDD LGKGGHELSL STGNAGGR--
A.chinensis(Chr3-CSD) ---------- ---------- ----QIPLCG PHSIIGRAVV VHA---DPDD LGKGGHELSK TTGNAGGR--
A.chinensis(Chr29-CSD) ---------- ---------- ---------- -----GRAVV VHA---DPDD LGKGGHELSK STGNAGGR--
V.vinifera(Chr14-CSDa) ---------- ---------- -----IPLTG SNSIVGRAVV VHA---DPDD LGKGGHELSK STGNAGGR--
V.vinifera(Chr14-CSDb) ---------- ---------- -----IPLTG SNSIVGRAVV VHA---DPDD LGKGGHELSK STGNAGGR--
M.domestica(Bb4037-CSD) ---------- ---------- ----QIPLAG PDSIIGRAVV VHA---DPDD LGKGGHELSK STGNAGGR--
M.domestica(Bb6170-CSD) ---------- ---------- ----QIPLTG PHSVIGRAVV VHG---DPDD LGKGGHELSK STGNAGGR--
T.has(NW_010961290.1-CSD) ---------- ---------- ----QIPLAG PNSIIGRAVV VHA---DPDD LGKGGHELSK STGNAGGR--
T.has(NW_010966881.1-CSD) ---------- ---------- ----QIPLTG PNSIIGRAVV VHA---DPDD LGKGGHELSK STGNAGGR--
G.max(Chr3-CSD) ---------- ---------- ----QIPLTG PNNIIGRAVV VHA---DPDD LGKGGHELSK TTGNAGGR--
G.max(Chr19-CSD) ---------- ---------- ----QIPLTG PNSIIGRAVV VHA---DSDD LGKGGHELSK TTGNAGGR--
B.rapa(Chr6-CSD) ---------- ---------- ----QIPLSG PNSIVGRAVV VHA---DPDD LGKGGHELSL ATGNAGGR--
B.rapa(Chr9-CSD) ---------- ---------- ----QIPLTG PNSIVGRAVV VHA---ERDD LGKGGHELSL STGNAGGR--
N.nucifera(MSc13-CSD) ---------- ---------- ----QIPLSG PNSIVGRAVV VHA---DPDD LGKGGHELSK STGNAGGR--
N.nucifera(MSc9-CSD) ---------- ---------- ----QIPLTG PYSIIGRAVV VHA---DPDD LGKGGHELSK STGNAGGR--
C.canephora(Chr2-CSD) ---------- ---------- ----QIPLTG PHSVVGRAVV VHA---DPDD LGRGGHELSK TTGNAGGR--
C.canephora(Chr3-CSD) ---------- ---------- ----QIPLSG PLSVIGRAVV VHG---DPDD LGKGGHELSK TTGNAGGR--
P.trichocarpa(Chr5-CSD) ---------- ---------- ----QIPLTG PHSIIGRAVV VHG---DPDD LGKGGHELSK TTGNAGGR--
P.trichocarpa(Chr13-CSD) ---------- ---------- ----QIPLTG PNSIVGRAVV VHA---DPDD LGKGGHELSK TTGNAGGR--
A.thaliana-CSD ---------- ---------- ----QIPLTG PNSIVGRAVV VHA---DPDD LGKGGHELSL ATGNAGGR--
P.axilleris-CSD ---------- ---------- ----QIPLTG PHSIIGRAVV VHA---DPDD LGKGGHELSK TTGNAGGR--
P.bretschneideri-CSD ---------- ---------- ----QIPLTG PHSIIGRAVV VHS---DPDD LGKGGHELSK STGNAGGR--
S.lycopersicum-CSD ---------- ---------- ----QIPLTG PQSIIGRAVV VHA---DPDD LGKGGHELSK STGNAGGR--
M.truncatula-CSD ---------- ---------- ----QIPLTG PNSIIGRAVV VHA---DPDD LGKGGHELSK TTGNAGGR--
V.radiata-CSD ---------- ---------- ----QIPLTG PNSIIGRAVV VHA---DPDD LGKGGHELSK STGNAGGR--
P.atrosanguinea-CSD ---------- ---------- ----QIPLTG PHSIIGRAVV VHA---DPDD LGKGGHELSK STGNAGGR--
C.limon-CSD ---------- ---------- ----QIPLSG PNSIIGRAVV VHA---DPDD LGKGGHELSK TTGNAGGR--
C.annuum-CSD ---------- ---------- ----QIPLTG PQSIIGRAVV VHA---DPDD LGKGGHELSK TTGNAGGR--
C.aromatica-CSD ---------- ---------- ----QIPLTG PHSIIGRAVV VHA---DPDD LGKGGHELSK STGNAGGR--
A.trichopoda-CSD ---------- ---------- ----QIPLSG PNSIIGRAVV VHA---DPDD LGKGGHELSK TTGNAGGR--
#10
#11

```
                                        360        370        380        390        400        410        420
                                 ....|....| ....|....| ....|....| ....|....| ....|....| ....|....| ....|....|
Monocots
O.sativa(Chr3-CSD1)              ---------- -------VAC G--------- ---------- ---------- IIG------- ----------
O.sativa(Chr7-CSD4)              ---------- -------VAC G--------- ---------- ---------- IIG------- ----------
O.brachyantha(Chr3-CSD)          ---------- -------VAC G--------- ---------- ---------- VIG------- ----------
O.brachyantha(Chr7-CSD)          ---------- -------VAC G--------- ---------- ---------- IIG------- ----------
S.italica(Chr2-CSD)              ---------- -------VAC G--------- ---------- ---------- IIG------- ----------
S.italica(Chr9-CSD)              ---------- -------IAC G--------- ---------- ---------- IIG------- ----------
S.bicolor(Chr1-CSD)              ---------- -------VAC G--------- ---------- ---------- IIG------- ----------
S.bicolor(Chr2-CSD)              ---------- -------VAC G--------- ---------- ---------- IIG------- ----------
E.guineensis(Chr5-CSD)           ---------- -------VAC G--------- ---------- ---------- IIG------- ----------
E.guineensis(Chr14-CSD)          ---------- -------VAC G--------- ---------- ---------- IIG------- ----------
O.thomaeum(001-CSDa)             ---------- -------VAC G--------- ---------- ---------- IIG------- ----------
O.thomaeum(001-CSDb)             ---------- -------VAC G--------- ---------- ---------- IIG------- ----------
O.thomaeum(027-CSD)              ---------- ---------- ---------- ---------- ---------- ---------- ----------
Z.mays(B73)(Chr7-CSD)            ---------- -------VAC GKLYRGVFPV SWYHWAPRLK QLTSEA--NT IEG------- ----------
Z.mays(B73)(Chr9-CSD)            ---------- --NSCWFSVC TFRWARAQQE HWKRRWPCCL WDHWTPGLKT IAG------- ----------
Z.mays(PH207)(Chr1-CSD)          ---------- -------VAC G--------- ---------- ---------- IIG------- ----------
Z.mays(PH207)(Chr7-CSD)          ---------- -------LDR AFNFIGLI-F ARYHWAPRLK QLTSEA--NT IEG------- ----------
Z.mays(PH207)(Chr9-CSD)          ---------- -------VAC G--------- ---------- ---------- IIG------- ----------
C.americanus(Chr5-CSD)           ---------- -------IAC G--------- ---------- ---------- MFTGSLAKLL PDAPCHAIWN
C.americanus(Chr7-CSD)           ---------- -------VAC G--------- ---------- ---------- IIG------- ----------
A.comosus(LG4-CSD)               ---------- -------VAC E--------- ---------- ---------- --G------- ----------
A.comosus(LG14-CSD)              ---------- -------VAC GGLLA----- ---------- ---------- LLDFRGRPSS YNTLTRSTLR
M.acuminata(Chr8-CSD)            ---------- -------VAC G--------- ---------- ---------- VIG------- ----------
T.aestivum(Ch2A-CSD)             ---------- -------VAC G--------- ---------- ---------- IIG------- ----------
T.aestivum(Ch2D-CSD)             ---------- -------VAC G--------- ---------- ---------- IIG------- ----------
S.spontaneum(Chr1B-CSD)          ---------- -------VAC G--------- ---------- ---------- IIG------- ----------
S.spontaneum(Chr1C-CSD)          ---------- -------VAC G--------- ---------- ---------- IIG------- ----------
S.spontaneum(Chr1D-CSD)          ---------- -------VAC G--------- ---------- ---------- IIG------- ----------
S.spontaneum(Chr2C-CSD)          ---------- -------SCC SISLVLAP-- ---------- ---------- QFG------- ----------
A.officinalis(V102-CSD)          ---------- -------VAC G--------- ---------- ---------- IIG------- ----------
A.officinalis(V108-CSD)          ---------- -------VGC G--------- ---------- ---------- IIG------- ----------
A.officinalis(V103-CSD)          ---------- -------EEL A--------- ---------- ---------- VST------- ----------
Z.japonica(Sca0001-CSD)          ---------- -------VAC G--------- ---------- ---------- IIG------- ----------
Z.japonica(Sca0003.1-CSD)        LDSRAERRLL PSPTDNVNAS G--------- ---------- ---------- HPK------- ----------
H.vulgare(Chr2-CSD)              ---------- -------VAC G--------- ---------- ---------- IIG------- ----------
H.vulgare(Chr5-CSD)              ---------- -------CA- ---------- ---------- ---------- ---------- ----------
H.vulgare(Chr5-CSDb)             ---------- -------CA- ---------- ---------- ---------- ---------- ----------
C.sim(Sca247-CSD)                ---------- -------VAC GG-------- ---------- ---------- FLG------- ----------
C.sim(Sca212-CSD)                ---------- -------VAC GG-------- ---------- ---------- FSG------- ----------
B.distachyon-CSD                 ---------- -------VAC G--------- ---------- ---------- IIG------- ----------
S.polyrhiza-CSD                  ---------- -------VAC G--------- ---------- ---------- VIG------- ----------
P.glaucum-CSD                    ---------- -------IAC G--------- ---------- ---------- IIG------- ----------
Z.marina-CSD                     ---------- -------VAC G--------- ---------- ---------- VIG------- ----------
Dicots
D.carota(Chr1-CSD)               ---------- -------VAC G--------- ---------- ---------- IIG------- ----------
D.carota(Chr9-CSD)               ---------- -------VAC G--------- ---------- ---------- IIG------- ----------
M.esculanta(Chr8-CSD)            ---------- -------VAC G--------- ---------- ---------- VIG------- ----------
M.esculanta(Chr9-CSD)            ---------- -------VAC G--------- ---------- ---------- IIG------- ----------
E.guttata(Sca7-CSD)              ---------- -------VAC G--------- ---------- ---------- IIG------- ----------
E.guttata(Sca14-CSD)             ---------- -------VAC G--------- ---------- ---------- IIG------- ----------
C.cajan(LG03-CSDa)               ---------- ---------- ---------- ---------- ---------- ---------- ----------
C.cajan(LG03-CSDb)               ---------- ---------- ---------- ---------- ---------- ---------- ----------
C.cajan(SC130189-CSD)            ---------- -------VAC G--------- ---------- ---------- KRR------- ----------
H.bra(NW018746327.1-CSD)         ---------- -------AAC G--------- ---------- ---------- IIG------- ----------
H.bra(NW018745806.1-CSD)         ---------- -------IAC G--------- ---------- ---------- IIG------- ----------
H.bra(NW018746407.1-CSD)         ---------- -------AAC G--------- ---------- ---------- IIG------- ----------
E.grandis(Chr2-CSD)              ---------- -------VAC G--------- ---------- ---------- VIG------- ----------
E.grandis(Chr8-CSD)              ---------- -------VAC G--------- ---------- ---------- LSC------- ----------
C.melo(Sca3-CSD)                 ---------- -------IGC G--------- ---------- ---------- VIG------- ----------
C.melo(Sca25-CSD)                ---------- -------VAC G--------- ---------- ---------- MHY------- ----------
C.clementina(Sca5-CSDa)          ---------- -------IAC G--------- ---------- ---------- VIG------- ----------
C.clementina(Sca5-CSDb)          ---------- ------ILGC C--------- ---------- ---------- FVY------- ----------
B.oleracea(Chr5-CSD)             ---------- -------VAC G--------- ---------- ---------- IIG------- ----------
B.oleracea(Chr8-CSD)             ---------- -------VAC G--------- ---------- ---------- IIG------- ----------
A.chinensis(Chr3-CSD)            ---------- -------VAC DGETGA---- ---------- ---------- ILS------- ----------
A.chinensis(Chr29-CSD)           ---------- -------LAC G--------- ---------- ---------- VIG------- ----------
V.vinifera(Chr14-CSDa)           ---------- -------VAC G--------- ---------- ---------- VIG------- ----------
V.vinifera(Chr14-CSDb)           ---------- -------VAC G--------- ---------- ---------- VIG------- ----------
M.domestica(Bb4037-CSD)          ---------- -------VAC G--------- ---------- ---------- IIW------- ----------
M.domestica(Bb6170-CSD)          ---------- -------VVL V--------- ---------- ---------- VLS------- ----------
T.has(NW_010961290.1-CSD)        ---------- -------VAC G--------- ---------- ---------- IIG------- ----------
T.has(NW_010966881.1-CSD)        ---------- -------VAC G--------- ---------- ---------- IIG------- ----------
G.max(Chr3-CSD)                  ---------- -------VAC G--------- ---------- ---------- IIG------- ----------
G.max(Chr19-CSD)                 ---------- -------VAC G--------- ---------- ---------- IIG------- ----------
B.rapa(Chr6-CSD)                 ---------- -------VAC G--------- ---------- ---------- IIG------- ----------
B.rapa(Chr9-CSD)                 ---------- -------VAC G--------- ---------- ---------- IIG------- ----------
N.nucifera(MSc13-CSD)            ---------- -------VAC G--------- ---------- ---------- IIG------- ----------
N.nucifera(MSc9-CSD)             ---------- -------VAC G--------- ---------- ---------- IIG------- ----------
C.canephora(Chr2-CSD)            ---------- -------VAC G--------- ---------- ---------- VIG------- ----------
C.canephora(Chr3-CSD)            ---------- -------VAC G--------- ---------- ---------- IIG------- ----------
P.trichocarpa(Chr5-CSD)          ---------- -------VAC G--------- ---------- ---------- IIG------- ----------
P.trichocarpa(Chr13-CSD)         ---------- -------VAC G--------- ---------- ---------- VIG------- ----------
A.thaliana-CSD                   ---------- -------VAC G--------- ---------- ---------- IIG------- ----------
P.axilleris-CSD                  ---------- -------VAC G--------- ---------- ---------- IIG------- ----------
P.bretschneideri-CSD             ---------- -------VAC G--------- ---------- ---------- IIG------- ----------
S.lycopersicum-CSD               ---------- -------IAC G--------- ---------- ---------- IIG------- ----------
M.truncatula-CSD                 ---------- -------VAC G--------- ---------- ---------- IIG------- ----------
V.radiata-CSD                    ---------- -------VAC G--------- ---------- ---------- IIG------- ----------
P.atrosanguinea-CSD              ---------- -------IAC G--------- ---------- ---------- IIG------- ----------
C.limon-CSD                      ---------- -------VAC G--------- ---------- ---------- IIG------- ----------
C.annuum-CSD                     ---------- -------VAC G--------- ---------- ---------- IIG------- ----------
C.aromatica-CSD                  ---------- -------IAC G--------- ---------- ---------- IIG------- ----------
A.trichopoda-CSD                 ---------- -------VAC G--------- ---------- ---------- IIG------- ----------

                                 ---- #12 -->           <---------- #13 ---------->   <----------------
```

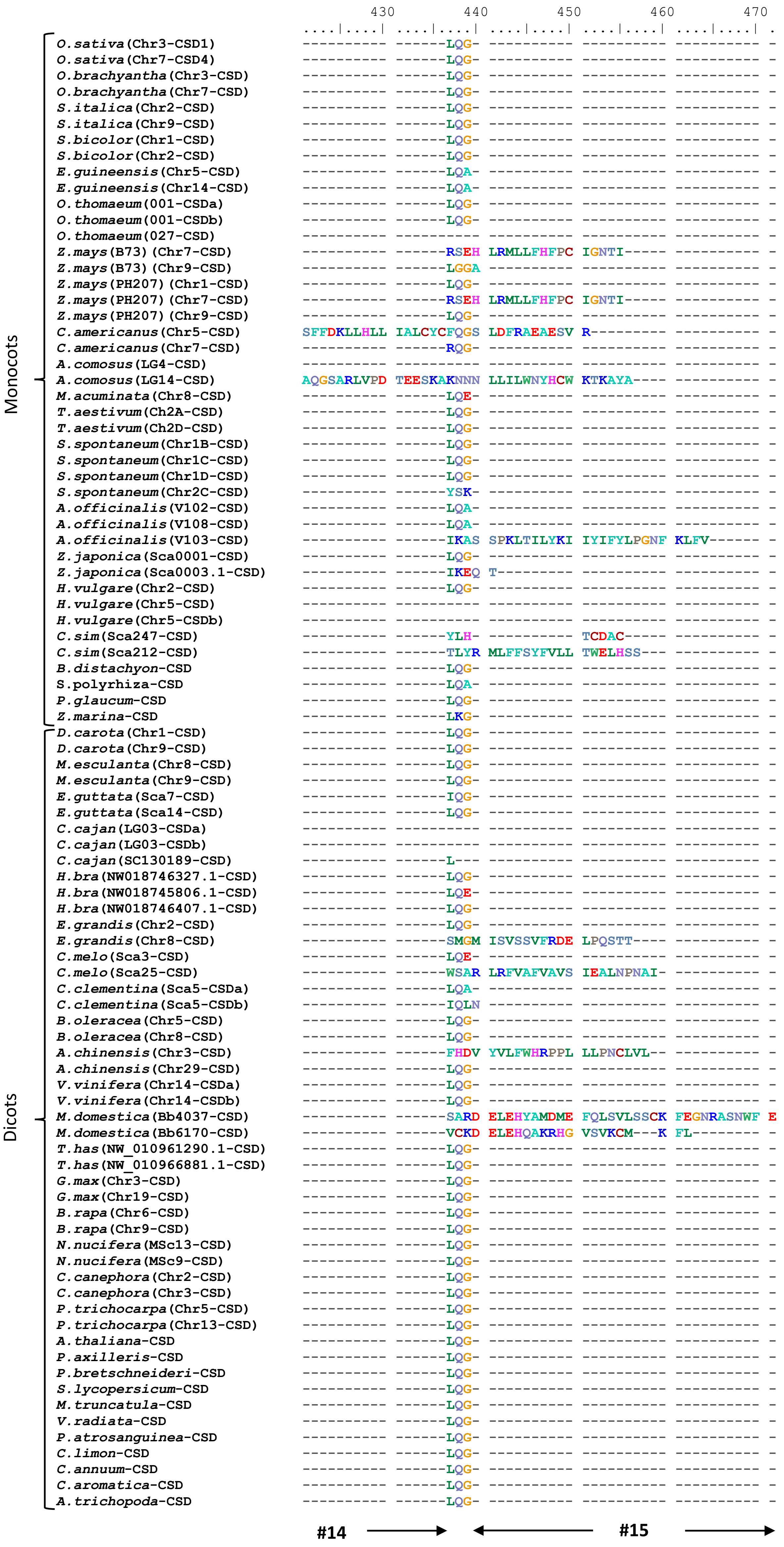

430 440 450 460 470
O.sativa(Chr3-CSD1) ---------- ------LQG- ---------- ---------- ---------- -
O.sativa(Chr7-CSD4) ---------- ------LQG- ---------- ---------- ---------- -
O.brachyantha(Chr3-CSD) ---------- ------LQG- ---------- ---------- ---------- -
O.brachyantha(Chr7-CSD) ---------- ------LQG- ---------- ---------- ---------- -
S.italica(Chr2-CSD) ---------- ------LQG- ---------- ---------- ---------- -
S.italica(Chr9-CSD) ---------- ------LQG- ---------- ---------- ---------- -
S.bicolor(Chr1-CSD) ---------- ------LQG- ---------- ---------- ---------- -
S.bicolor(Chr2-CSD) ---------- ------LQG- ---------- ---------- ---------- -
E.guineensis(Chr5-CSD) ---------- ------LQA- ---------- ---------- ---------- -
E.guineensis(Chr14-CSD) ---------- ------LQA- ---------- ---------- ---------- -
O.thomaeum(001-CSDa) ---------- ------LQG- ---------- ---------- ---------- -
O.thomaeum(001-CSDb) ---------- ------LQG- ---------- ---------- ---------- -
O.thomaeum(027-CSD) ---------- ---------- ---------- ---------- ---------- -
Z.mays(B73)(Chr7-CSD) ---------- ------RSEH LRMLLFHFPC IGNTI----- ---------- -
Z.mays(B73)(Chr9-CSD) ---------- ------LGGA ---------- ---------- ---------- -
Z.mays(PH207)(Chr1-CSD) ---------- ------LQG- ---------- ---------- ---------- -
Z.mays(PH207)(Chr7-CSD) ---------- ------RSEH LRMLLFHFPC IGNTI----- ---------- -
Z.mays(PH207)(Chr9-CSD) ---------- ------LQG- ---------- ---------- ---------- -
C.americanus(Chr5-CSD) SFFDKLLHLL IALCYCFQGS LDFRAEAESV R--------- ---------- -
C.americanus(Chr7-CSD) ---------- ------RQG- ---------- ---------- ---------- -
A.comosus(LG4-CSD) ---------- ---------- ---------- ---------- ---------- -
A.comosus(LG14-CSD) AQGSARLVPD TEESKAKNNN LLILWNYHCW KTKAYA---- ---------- -
M.acuminata(Chr8-CSD) ---------- ------LQE- ---------- ---------- ---------- -
T.aestivum(Ch2A-CSD) ---------- ------LQG- ---------- ---------- ---------- -
T.aestivum(Ch2D-CSD) ---------- ------LQG- ---------- ---------- ---------- -
S.spontaneum(Chr1B-CSD) ---------- ------LQG- ---------- ---------- ---------- -
S.spontaneum(Chr1C-CSD) ---------- ------LQG- ---------- ---------- ---------- -
S.spontaneum(Chr1D-CSD) ---------- ------LQG- ---------- ---------- ---------- -
S.spontaneum(Chr2C-CSD) ---------- ------YSK- ---------- ---------- ---------- -
A.officinalis(V102-CSD) ---------- ------LQA- ---------- ---------- ---------- -
A.officinalis(V108-CSD) ---------- ------LQA- ---------- ---------- ---------- -
A.officinalis(V103-CSD) ---------- ------IKAS SPKLTILYKI IYIFYLPGNF KLFV------ -
Z.japonica(Sca0001-CSD) ---------- ------LQG- ---------- ---------- ---------- -
Z.japonica(Sca0003.1-CSD) ---------- ------IKEQ T--------- ---------- ---------- -
H.vulgare(Chr2-CSD) ---------- ------LQG- ---------- ---------- ---------- -
H.vulgare(Chr5-CSD) ---------- ---------- ---------- ---------- ---------- -
H.vulgare(Chr5-CSDb) ---------- ---------- ---------- ---------- ---------- -
C.sim(Sca247-CSD) ---------- ------YLH- ---------- TCDAC----- ---------- -
C.sim(Sca212-CSD) ---------- ------TLYR MLFFSYFVLL TWELHSS--- ---------- -
B.distachyon-CSD ---------- ------LQG- ---------- ---------- ---------- -
S.polyrhiza-CSD ---------- ------LQA- ---------- ---------- ---------- -
P.glaucum-CSD ---------- ------LQG- ---------- ---------- ---------- -
Z.marina-CSD ---------- ------LKG- ---------- ---------- ---------- -
D.carota(Chr1-CSD) ---------- ------LQG- ---------- ---------- ---------- -
D.carota(Chr9-CSD) ---------- ------LQG- ---------- ---------- ---------- -
M.esculanta(Chr8-CSD) ---------- ------LQG- ---------- ---------- ---------- -
M.esculanta(Chr9-CSD) ---------- ------LQG- ---------- ---------- ---------- -
E.guttata(Sca7-CSD) ---------- ------IQG- ---------- ---------- ---------- -
E.guttata(Sca14-CSD) ---------- ------LQG- ---------- ---------- ---------- -
C.cajan(LG03-CSDa) ---------- ---------- ---------- ---------- ---------- -
C.cajan(LG03-CSDb) ---------- ---------- ---------- ---------- ---------- -
C.cajan(SC130189-CSD) ---------- ------L--- ---------- ---------- ---------- -
H.bra(NW018746327.1-CSD) ---------- ------LQG- ---------- ---------- ---------- -
H.bra(NW018745806.1-CSD) ---------- ------LQE- ---------- ---------- ---------- -
H.bra(NW018746407.1-CSD) ---------- ------LQG- ---------- ---------- ---------- -
E.grandis(Chr2-CSD) ---------- ------LQG- ---------- ---------- ---------- -
E.grandis(Chr8-CSD) ---------- ------SMGM ISVSSVFRDE LPQSTT---- ---------- -
C.melo(Sca3-CSD) ---------- ------LQE- ---------- ---------- ---------- -
C.melo(Sca25-CSD) ---------- ------WSAR LRFVAFVAVS IEALNPNAI- ---------- -
C.clementina(Sca5-CSDa) ---------- ------LQA- ---------- ---------- ---------- -
C.clementina(Sca5-CSDb) ---------- ------IQLN ---------- ---------- ---------- -
B.oleracea(Chr5-CSD) ---------- ------LQG- ---------- ---------- ---------- -
B.oleracea(Chr8-CSD) ---------- ------LQG- ---------- ---------- ---------- -
A.chinensis(Chr3-CSD) ---------- ------FHDV YVLFWHRPPL LLPNCLVL-- ---------- -
A.chinensis(Chr29-CSD) ---------- ------LQG- ---------- ---------- ---------- -
V.vinifera(Chr14-CSDa) ---------- ------LQG- ---------- ---------- ---------- -
V.vinifera(Chr14-CSDb) ---------- ------LQG- ---------- ---------- ---------- -
M.domestica(Bb4037-CSD) ---------- ------SARD ELEHYAMDME FQLSVLSSCK FEGNRASNWF E
M.domestica(Bb6170-CSD) ---------- ------VCKD ELEHQAKRHG VSVKCM---K FL-------- -
T.has(NW_010961290.1-CSD) ---------- ------LQG- ---------- ---------- ---------- -
T.has(NW_010966881.1-CSD) ---------- ------LQG- ---------- ---------- ---------- -
G.max(Chr3-CSD) ---------- ------LQG- ---------- ---------- ---------- -
G.max(Chr19-CSD) ---------- ------LQG- ---------- ---------- ---------- -
B.rapa(Chr6-CSD) ---------- ------LQG- ---------- ---------- ---------- -
B.rapa(Chr9-CSD) ---------- ------LQG- ---------- ---------- ---------- -
N.nucifera(MSc13-CSD) ---------- ------LQG- ---------- ---------- ---------- -
N.nucifera(MSc9-CSD) ---------- ------LQG- ---------- ---------- ---------- -
C.canephora(Chr2-CSD) ---------- ------LQG- ---------- ---------- ---------- -
C.canephora(Chr3-CSD) ---------- ------LQG- ---------- ---------- ---------- -
P.trichocarpa(Chr5-CSD) ---------- ------LQG- ---------- ---------- ---------- -
P.trichocarpa(Chr13-CSD) ---------- ------LQG- ---------- ---------- ---------- -
A.thaliana-CSD ---------- ------LQG- ---------- ---------- ---------- -
P.axilleris-CSD ---------- ------LQG- ---------- ---------- ---------- -
P.bretschneideri-CSD ---------- ------LQG- ---------- ---------- ---------- -
S.lycopersicum-CSD ---------- ------LQG- ---------- ---------- ---------- -
M.truncatula-CSD ---------- ------LQG- ---------- ---------- ---------- -
V.radiata-CSD ---------- ------LQG- ---------- ---------- ---------- -
P.atrosanguinea-CSD ---------- ------LQG- ---------- ---------- ---------- -
C.limon-CSD ---------- ------LQG- ---------- ---------- ---------- -
C.annuum-CSD ---------- ------LQG- ---------- ---------- ---------- -
C.aromatica-CSD ---------- ------LQG- ---------- ---------- ---------- -
A.trichopoda-CSD ---------- ------LQG- ---------- ---------- ---------- -
Monocots
Dicots
#14
#15

Supplementary Figure 8: Analysis of domains/important features of the proteins encoded by CuZn SOD (*CSD*) genes originated due to block/tandem chromosomal duplication events in monocot plant genomes. The duplication events were analyzed at Monocots PLAZA 4.5 database (Bel et al., 2018), while the analysis of conserved domains was carried out at Conserved Domain Database (CDD, https://www.ncbi.nlm.nih.gov/structure/cdd/). Following designation for genes is used below for different SOD isoforms; CytCSD: cytosolic CuZn SOD, ChlCSD: chloroplastic CuZn SOD and PerCSD: peroxisomal CuZn SOD. The designation used to indicate genomic locations (chromosome, scaffold, linkage group etc.) are as per the PLAZA database (Chr: chromosome, Sca: Scaffold, MSca: Megascaffold, LG: Linkage groups etc. Domains, and important amino acids are indicated.

A) ***Oryza sativa***: Domain analysis of two cytosolic CSDs (CytCSDs: OsCSD1, OsCSD4) originated in due to a block duplication event between Chr3 and Chr7

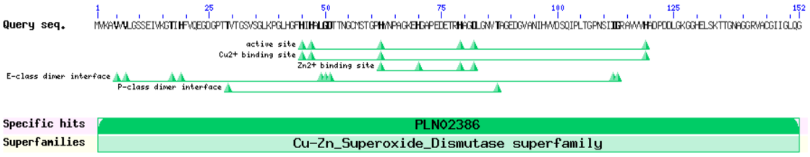

Chr3-OsCSD1

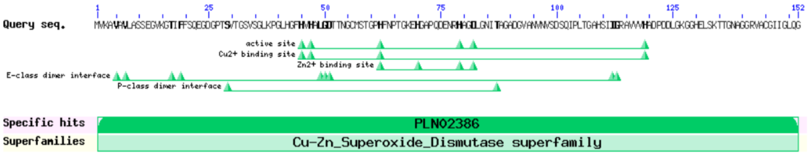

Chr7-OsCSD4

B) ***Oryza brachyantha***: Domain analysis of two cytosolic CSDs (CytCSDs) originated due to a block duplication between Chr3 and Chr7

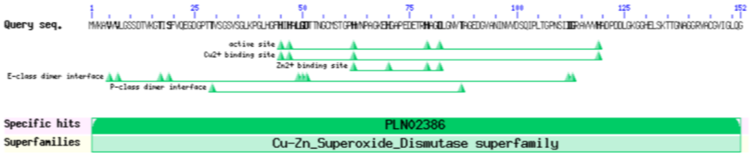

Chr3-CytCSD

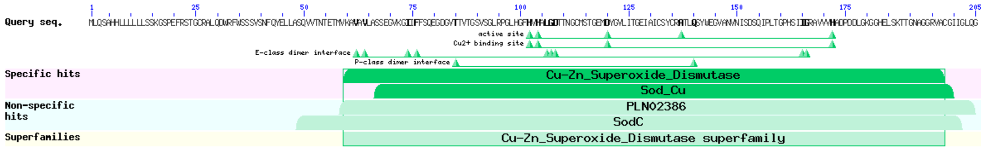

Chr7-CytCSD

C) ***Setaria italica***: Domain analysis of two cytosolic CSDs (CytCSDs) originated due to a block duplication event between Chr9 and Chr2.

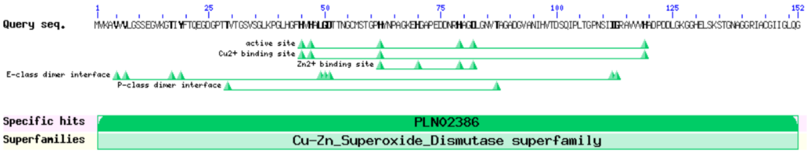

Chr9-CytCSD

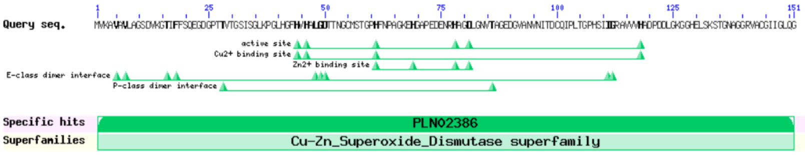

Chr2-CytCSD

D) ***Sorghum bicolor***: Domain analysis of two cytosolic CSDs (CytCSDs) originated due to a block duplication event between Chr1 and Chr2.

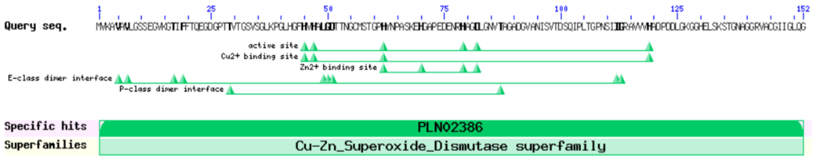

Chr1-CytCSD

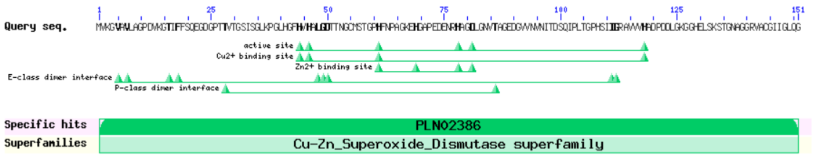

Chr7-CytCSD

E) ***Elaeis guineensis***: Domain analysis of three cytosolic CSDs (CytCSDs) originated due to two block duplication between Chr5 and Chr14.

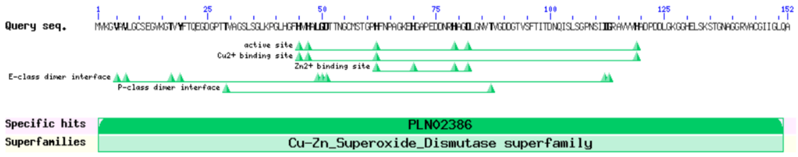

Chr5-CytCSD

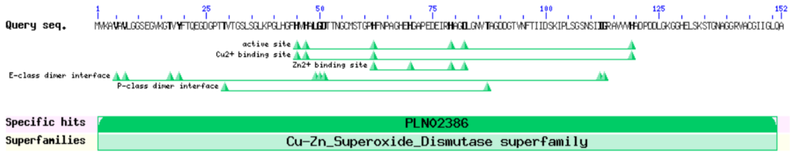

Chr14-CytCSDa

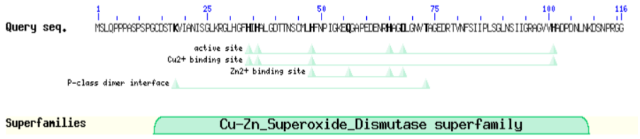

Chr14-CytCSDb

F) ***Oropetium thomaeum***: Domain analysis of three cytosolic CSDs (CytCSDs) originated due to a block duplication event between chromosome Og_2014112_001 and Og_2014112_027, and a tandem duplication event (gene ids: Oropetium_20150105_01325 and Oropetium_20150105_01319) within chromosome Og_2014112_001

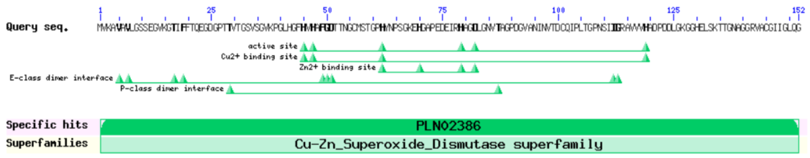

Og_2014112_001-CytCSDa

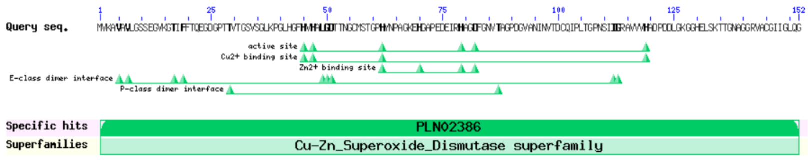

Og_2014112_001-CytCSDb

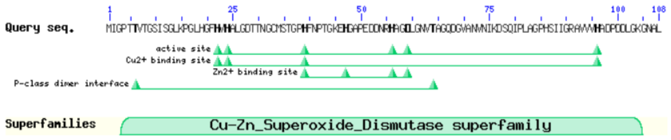

Og_2014112_027-CytCSD

G) ***Zea mays* (B73):** Domain analysis of two cytosolic CSDs (CytCSDs) originated due to a block duplication event between Chr7 and Chr9.

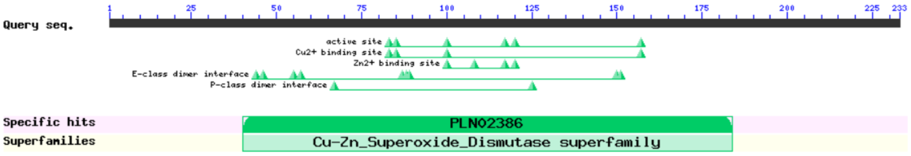

Chr7-CytCSD

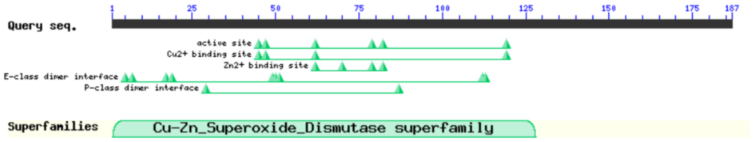

Chr9-CytCSD

H) ***Zea mays* (PH207):** Domain analysis of three cytosolic CSDs (CytCSDs) originated due to two block duplication events (between Chr1 and Chr9; Chr7 and Chr9).

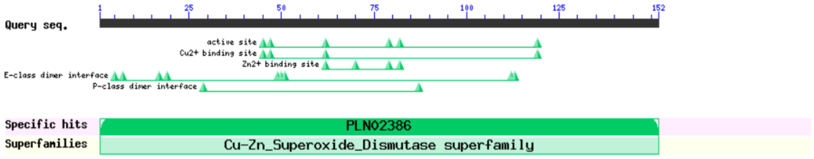

Chr1-CytCSD

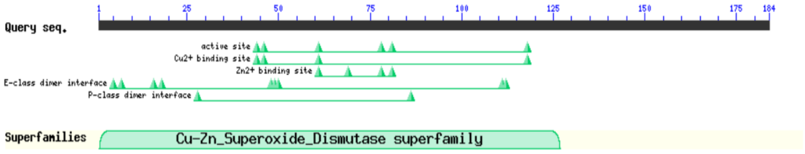

Chr7-CytCSD

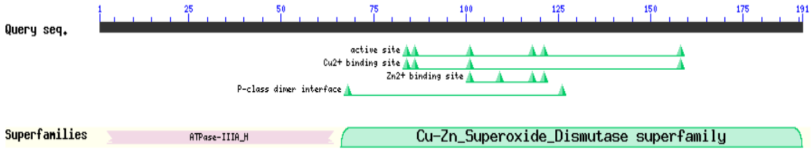

Chr9-CytCSD

I) ***Cenchrus americanus*:** Domain analysis of two cytosolic CSDs (CytCSDs) originated due to a block duplication event between Chr7 and Chr5.

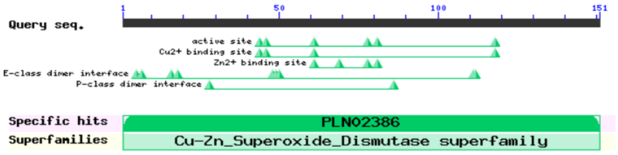

Chr7-CytCSD

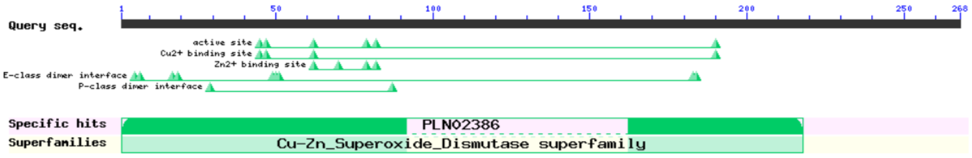

Chr5-CytCSD

J) ***Ananas comosus*:** Domain analysis of two cytosolic CSDs (CytCSDs) originated due to a block duplication event between LG4 and LG14.

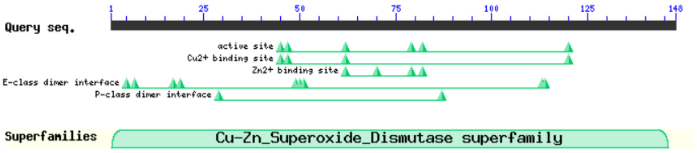

LG4-CytCSD

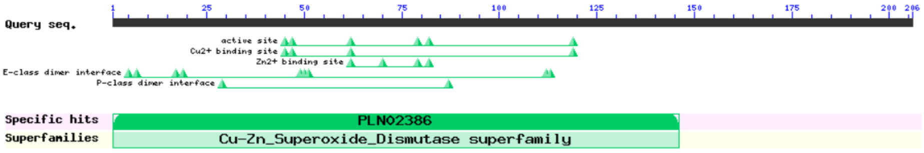

LG14-CytCSD

K) ***Musa acuminata*:** Domain analysis of two chloroplastic CSDs (ChlCSDs) originated due to a block duplication between Sca00015.1 and Sca00078.1.

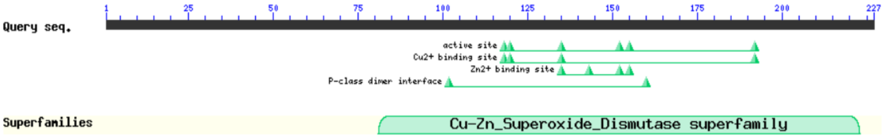

Chr9-ChlCSD

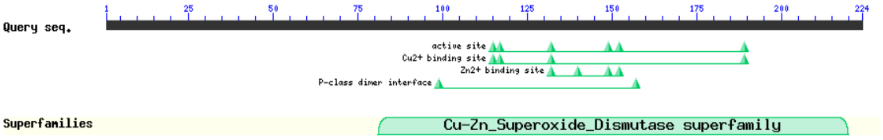

Chr10-ChlCSD

L) ***Asparagus officinalis*:** Domain analysis of two chloroplastic CSDs (ChlCSDs) originated due to a block duplication between chromosome number V1_04 and V1_10.

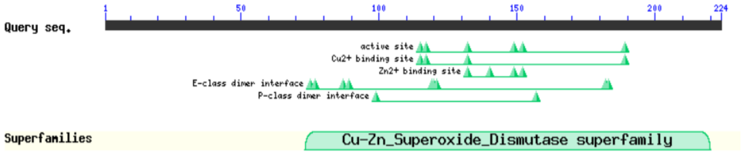

V1_04-ChlCSD

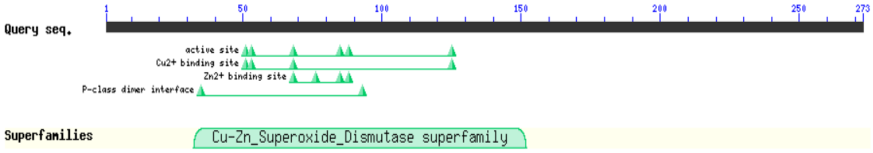

V1_10-ChlCSD

M) ***Zoysia japonica* ssp. *nagirizaki*:** Domain analysis of two chloroplastic CSDs (ChlCSDs) originated due to a block duplication event between chromosome number Sca00015.1 and Sca00078.1.

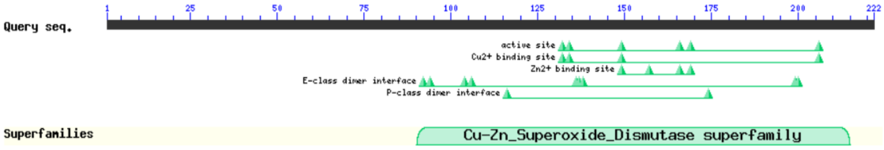

Sca00015.1-ChlCSD

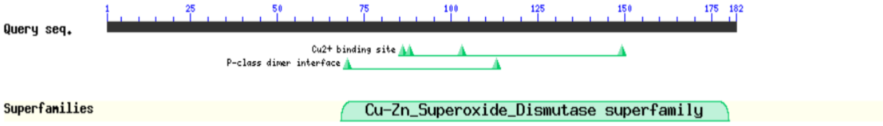

Sca00078.1-ChlCSD

N) ***Triticum aestivum*:**

i) Domain analysis of two cytosolic CSDs (CytCSDs) originated due to a block duplication between chromosome Chr2A and Chr2D.

Chr2A-CytCSD

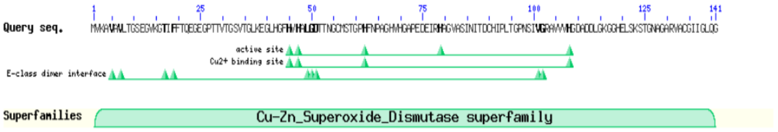

Chr2D-CytCSD

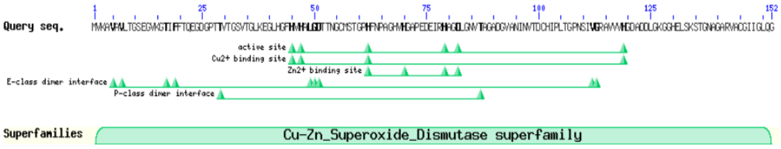

ii) Domain analysis of three chloroplastic CSDs (ChlCSDs) originated due to block duplication events between chromosome Chr7A, Chr7B and Chr7D.

Chr7A-ChlCSD

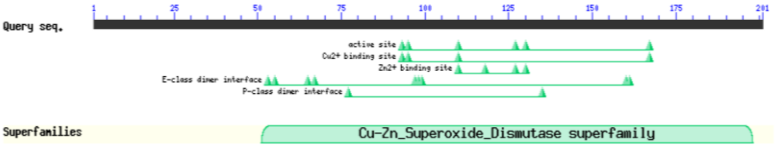

Chr7B-ChlCSD

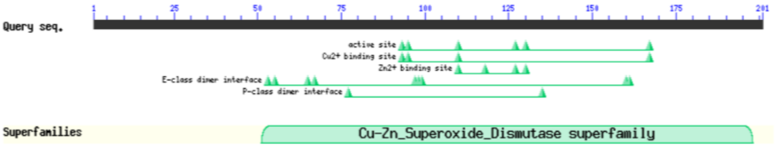

Chr7D-ChlCSD

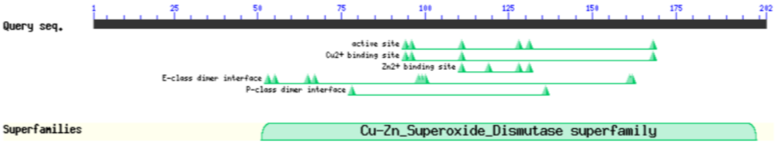

iii) Domain analysis of three peroxisomal CSDs (PerCSDs) due to block duplication events between chromosome Chr4A, Chr4B and Chr4D.

Chr4A-PerCSD

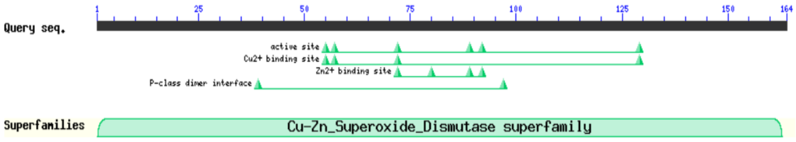

Chr4B-PerCSD

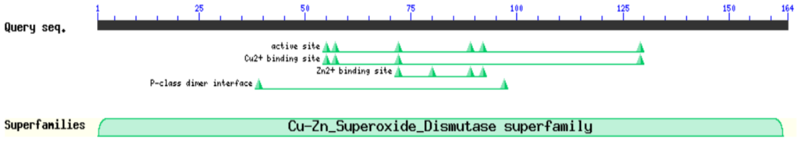

Chr4D-PerCSD

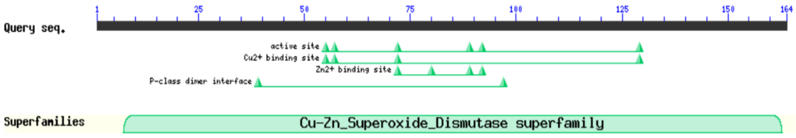

O) ***Saccharum spontaneum*:**

i) Domain analysis of four cytosolic CSDs (CytCSDs) originated due to block duplication events involving four chromosomes (Chr1B, IC, ID, and 2C).

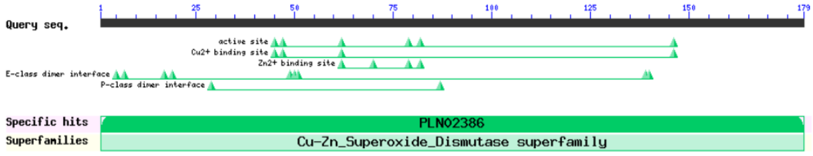

Chr1C-CytCSD

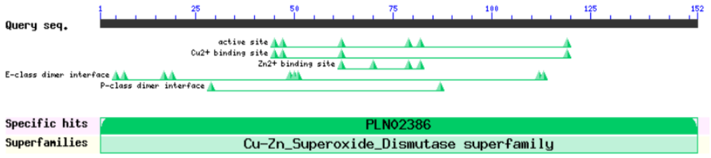

Chr1D-CytCSD

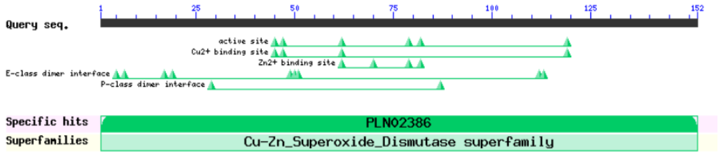

Chr1B-CytCSD

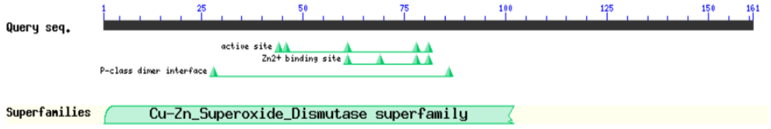

Chr2C-CytCSD

ii) Domain analysis of two chloroplastic CSDs (ChlCSDs) originated due to a block duplication involving events between Chr6B and Chr6D.

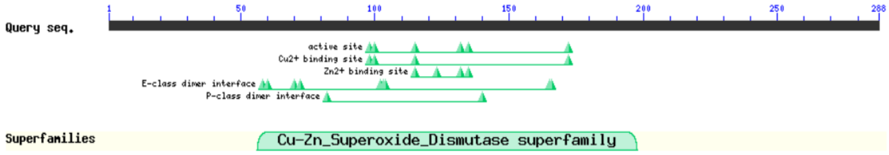

Chr6B-ChlCSD

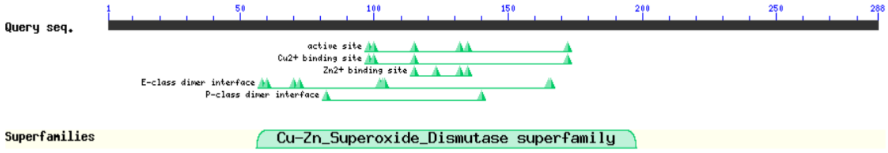

Chr6D-ChlCSD

iii) Domain analysis of four peroxisomal CSDs (PerCSDs) originated due to block duplication events involving four chromosomes (Chr1A, 1B, 1C, and 1D).

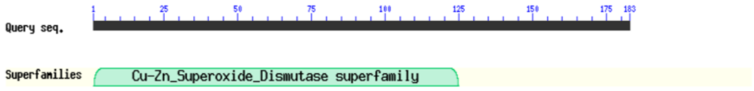

Chr1A-PerCSD3

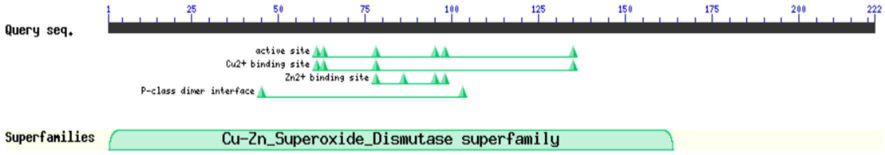

Chr1B-PerCSD

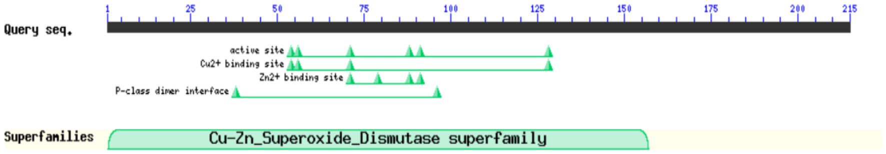

Chr1C-PerCSD

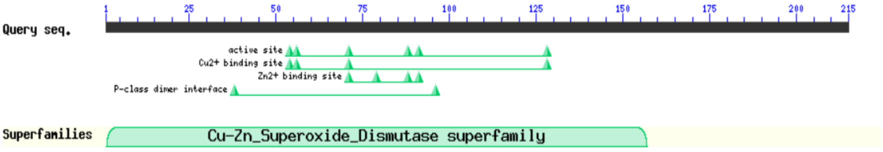

Chr1D-PerCSD

P) ***Hordeum vulgare*:** Domain analysis of three cytosolic CSDs (CytCSD) present in the genome. No block/tandem duplication events predicted

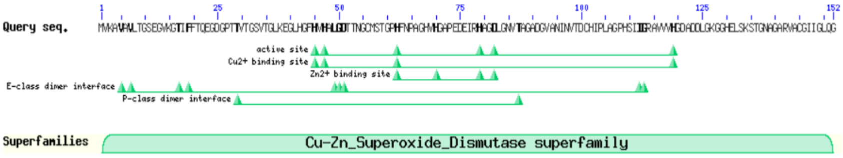

Chr2-CytCSD

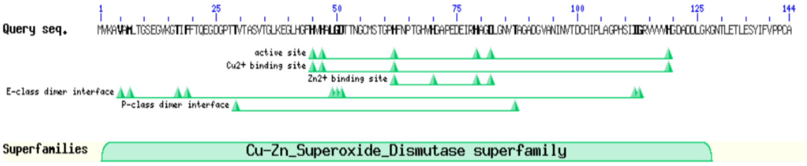

Chr5-CytCSDa

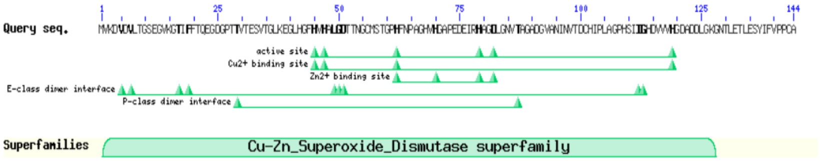

Chr5-CytCSDb

Supplementary Figure 9: Analysis of domains/important features of the proteins encoded by CuZn SOD (*CSD*) genes originated due to block/tandem chromosomal duplication events in dicot plant genomes. The duplication events were analyzed at Monocots PLAZA 4.5 database (Bel et al., 2018), while the analysis of conserved domains was carried out at Conserved Domain Database (CDD, https://www.ncbi.nlm.nih.gov/structure/cdd/). Following designation for genes is used below for different SOD isoforms; CytCSD: cytosolic CuZn SOD, ChlCSD: chloroplastic CuZn SOD and PerCSD: peroxisomal CuZn SOD. The designation used to indicate genomic locations (chromosome, scaffold, linkage group etc.) are as per the PLAZA database (Chr: chromosome, Sca: Scaffold, MSca: Megascaffold, LG: Linkage groups etc. Domains, and important amino acids are indicated.

A) ***Daucus carota***: Domain analysis of two cytosolic CSDs (CytCSDs) originated in due to a block duplication event between Chr1 and Chr9.

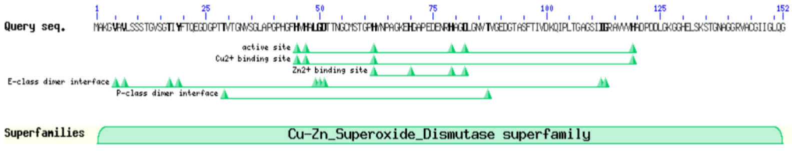

Chr1-CytCSD

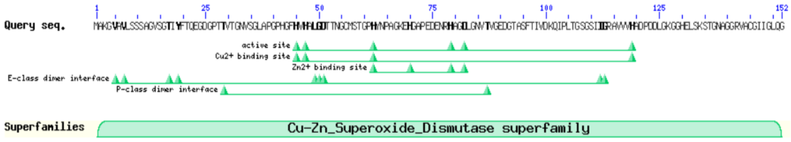

Chr9-CytCSD

B) ***Manihot esculanta***: Domain analysis of two cytosolic CSDs (CytCSDs) originated due to a block duplication event between chromosomes Chr8 and Chr9.

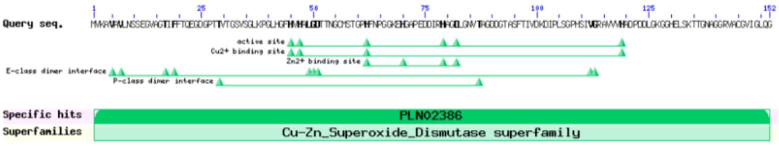

Chr8-CytCSD

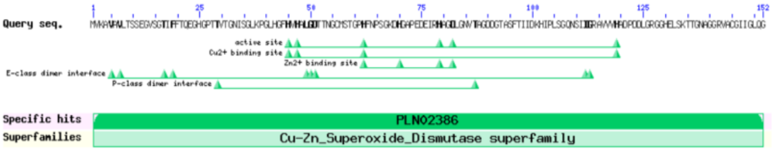

Chr9-CytCSD

C) ***Erythranthe guttata***: Domain analysis of two cytosolic CSDs (CytCSDs) originated in due to a block duplication event between chromosome Sca7 and Sca14.

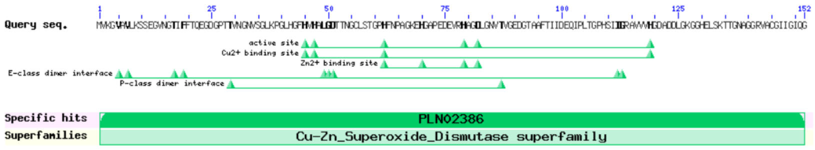

Sca7-CytCSD

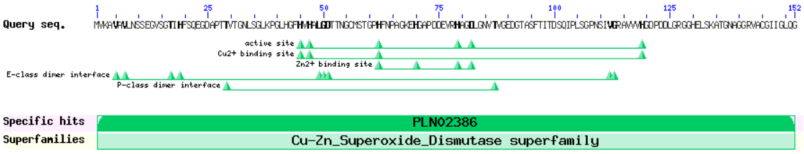

Sca14-CytCSD

D) ***Cajanus cajan***: Domain analysis of two cytosolic CSDs (CytCSDs) originated in due to a tandem duplication event within chromosome CcLG03 (gene ids: 10731.g and 10734.g). Another CytCSD on chromosome Sca0819 (gene id: g.4037.5) does not show evidence of any duplication event.

Sca0819-CytCSD

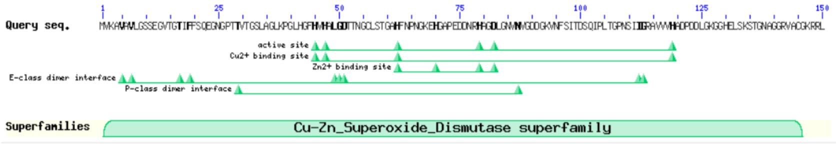

CcLG03-CytCSD (10731.g)

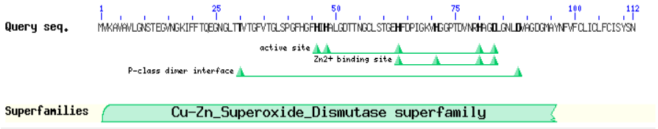

CcLG03-CytCSD (10734.g)

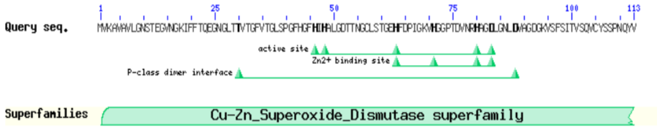

E) ***Hevea brasiliensis***: Domain analysis of three cytosolic CSDs (CytCSDs) originated due to a block duplication events between chromosomes NW_018746327.1, NW_018745806.1 and NW_018746407.1

NW_018746327.1-CytCSD

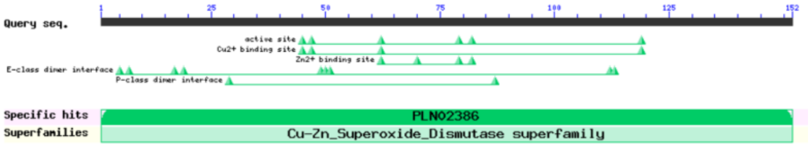

NW_018745806.1-CytCSD

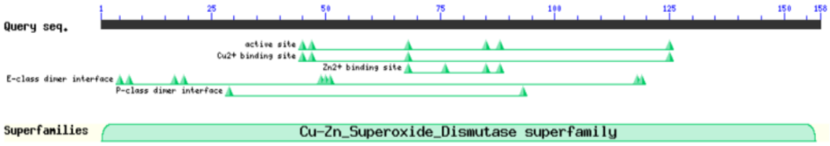

NW_018746407.1-CytCSD

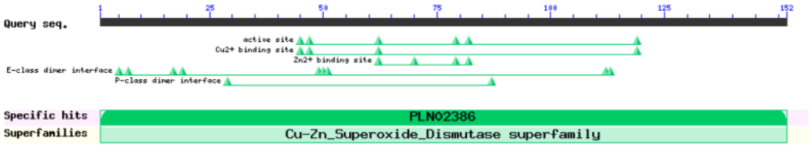

F) ***Eucalyptus grandis***: Domain analysis of two cytosolic CSDs (CytCSDs) originated in due to a block duplication event between chromosome Chr2 and Chr8.

Chr2-CytCSD

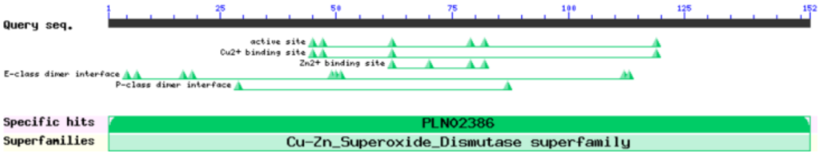

Chr8-CytCSD

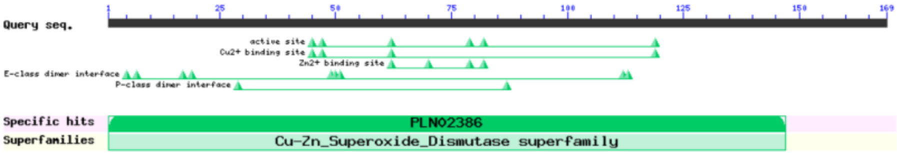

G) ***Cucumis melo***: Domain analysis of two cytosolic CSDs (CytCSDs) originated due to a block duplication between chromosomes Sca3and Sca25.

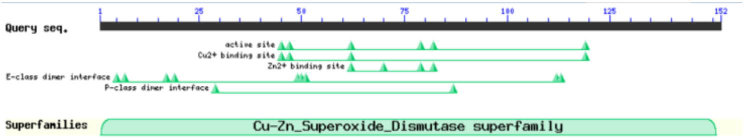

Sca3-CytCSD

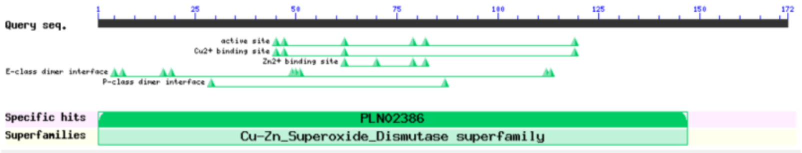

Sca25-CytCSD

H) ***Citrus clementina***: Domain analysis of two cytosolic CSDs (CytCSDs) originated due to a tandem duplication event (gene ids: Ciclev10002756m.g and Ciclev10002619m.g) within chromosome Sca5.

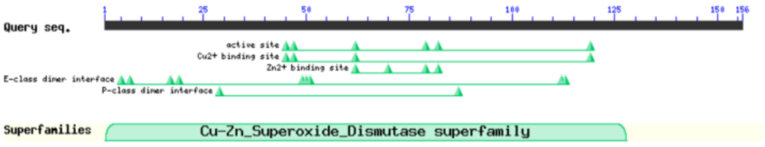

Sca5-CytCSD (10002756m.g)

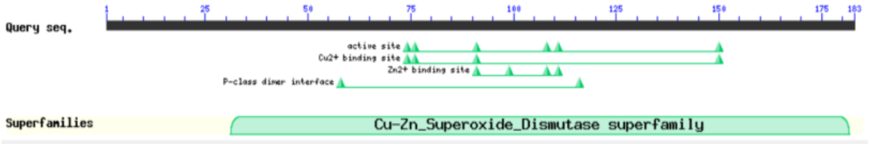

Sca5-CytCSD (10002619m.g)

I) ***Brassica oleracea***: Domain analysis of two cytosolic CSDs (CytCSDs) originated due to a block duplication event between Chr5 and Chr8.

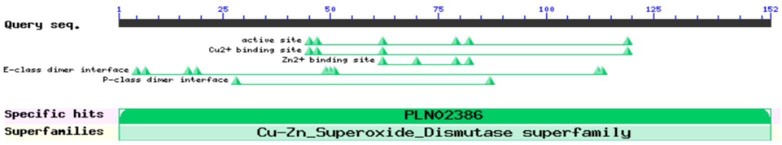

Chr5-CytCSD

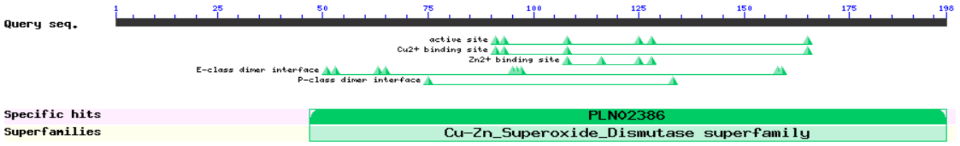

Chr8-CytCSD

J) ***Actinidia chinensis***: Domain analysis of two cytosolic CSDs (CytCSDs) originated in due to a tandem duplication event between Chr3 and Chr29.

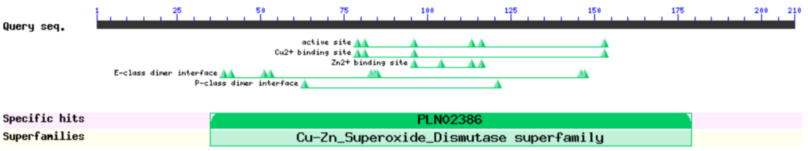

Chr3-CytCSD

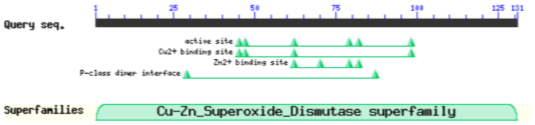

Chr29-CytCSD

K) ***Vitis vinifera***: Domain analysis of three cytosolic CSDs (CytCSDs) originated due to tandem duplication events (gene ids: GSVIVG01021922001, GSVIVG01021949001 and GSVIVG01021959001) within chromosome Chr14.

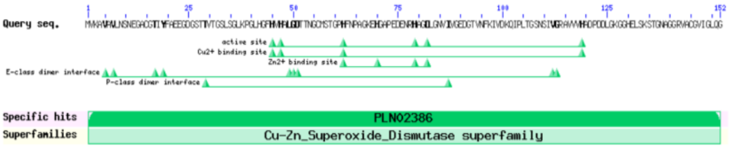

Chr14-CytCSD (G01021959001)

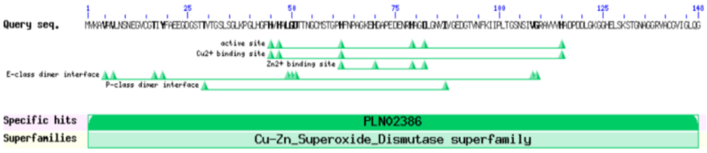

Chr14-CytCSD (G01021949001)

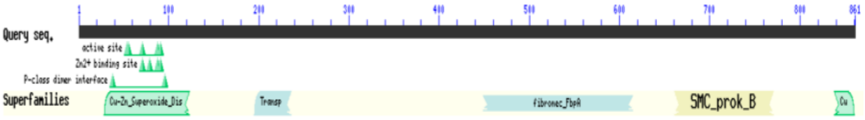

Chr14-CytCSD (G01021922001)

L) ***Malus domestica***: Domain analysis of two cytosolic CSDs (CytCSDs) originated in due to a tandem duplication event within chromosome backbone Bb6170 (gene ids: g.6170.7 and g.6170.8). Another CytCSD on chromosome backbone Bb4037 (gene id: g.4037.5) shows no indication of any duplication event.

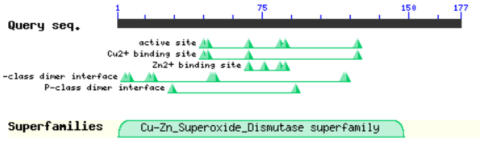

Bb4037-CytCSD (g.4037.5)

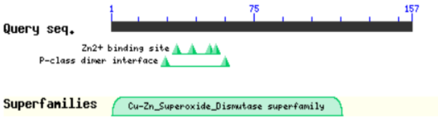

Bb6170-CytCSD (g.6170.7)

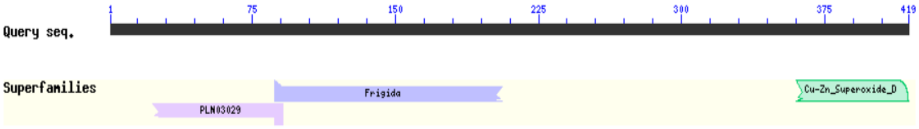

Bb6170-CytCSD (g.6170.8)

M) ***Vigna radiata var. radiata***: Domain analysis of two chloroplastic CSDs (ChlCSD) originated due to a block duplication between chromosome Chr2 and Chr4.

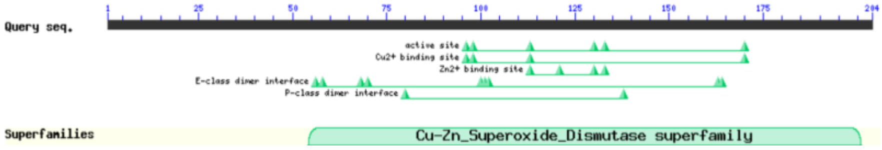

Chr2-ChlCSD

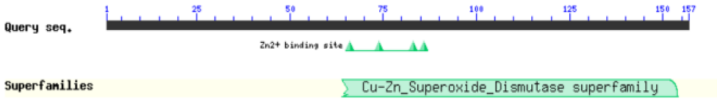

Chr4-ChlCSD

N) ***Arachis ipaensis***: Domain analysis of two chloroplastic CSDs (ChlCSDs) originated due to a block duplication event between chromosome Araip.B03 and Araip.B08.

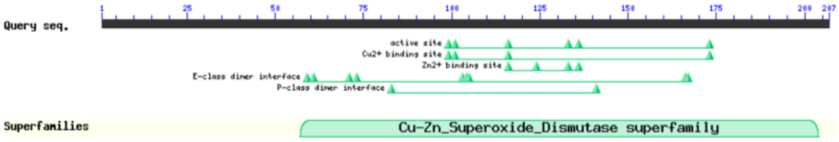

Araip.B03-ChlCSD

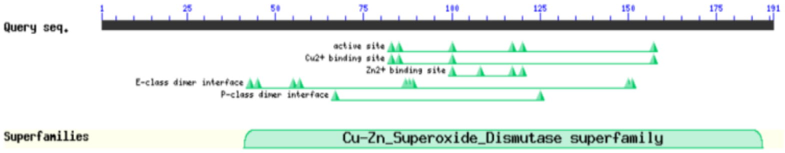

Araip.B08-ChlCSD

O) ***Tarenaya hassleriana***:

i) Domain analysis of two cytosolic CSDs (CytCSD) originated due to a block duplication event between chromosome NW_010961290.1 and NW_010966881.1.

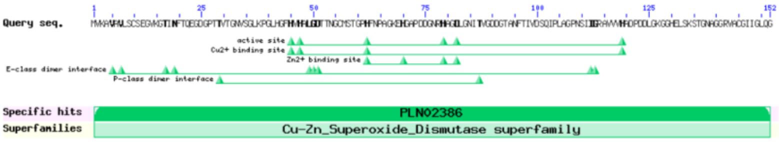

NW_010961290.1-CytCSD

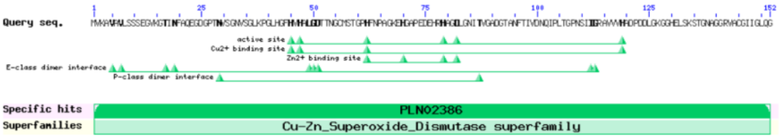

NW_010966881.1-CytCSD

ii) Domain analysis of two chloroplastic CSDs (ChlCSDs) originated due to a block duplication event between chromosome NW_010967707.1 and NW_010965696.1.

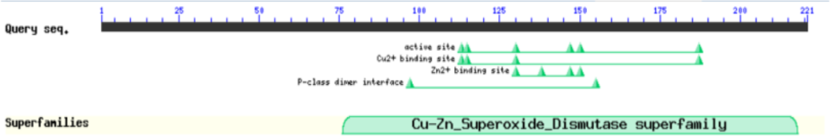

NW_010967707.1-ChlCSD

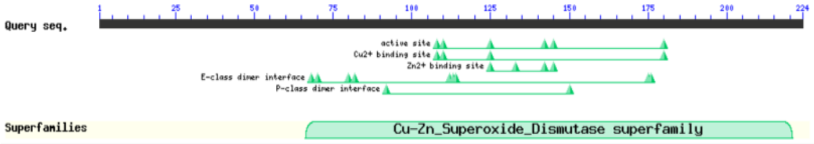

NW_010965696.1-ChlCSD

P) ***Glycine max***:

i) Domain analysis of two cytosolic CSDs (CytCSD) originated due to a block duplication event between Chromosome Chr3 and Chr19.

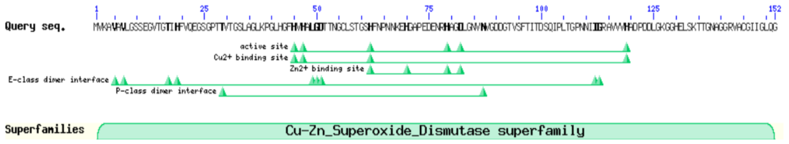

Chr3-CytCSD

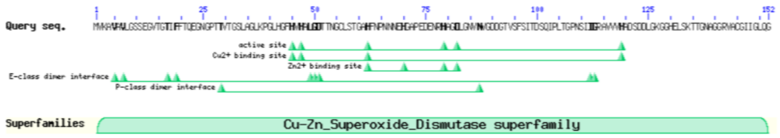

Chr19-CytCSD

ii) Domain analysis of three chloroplastic CSDs (ChlCSDs) originated due to two block duplication events between Chromosome Chr11 and Chr12.

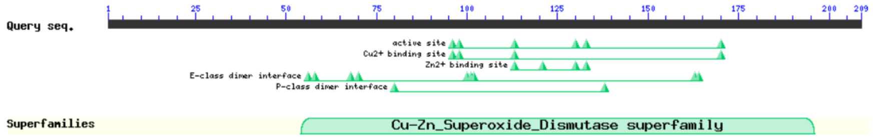

Chr11-ChlCSD

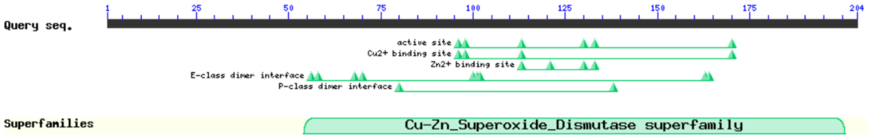

Chr12-ChlCSDa

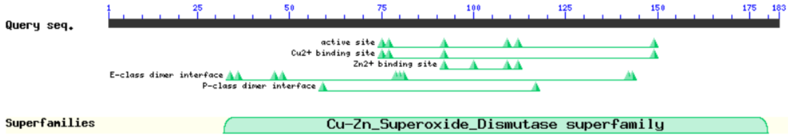

Chr12-ChlCSDb

Q) ***Brassica rapa*:**

i) Domain analysis of two cytosolic CSDs (CytCSDs) originated due to a block duplication event between chromosome Chr6 and Chr9

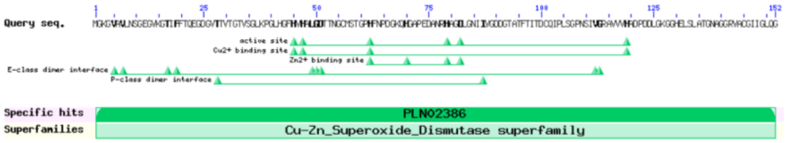

Chr6-CytCSD

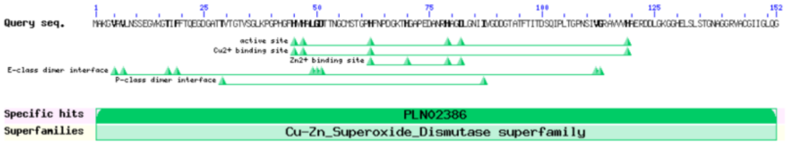

Chr9-CytCSD

ii) ***Brassica rapa*:** Domain analysis of two chloroplastic CSDs (ChlCSDs) originated due to a block duplication event between chromosome Chr4 and Chr7

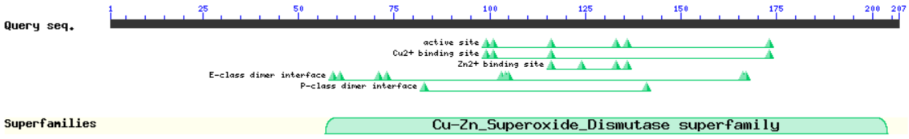

Chr4-CytCSD

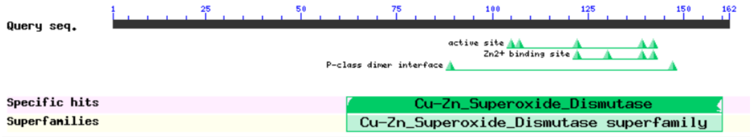

Chr7-CytCSD

R) ***Nelumbo nucifera*:**

i) Domain analysis of two cytosolic CSDs (CytCSDs) originated due to a block duplication event between chromosome MSca9 and MSca13.

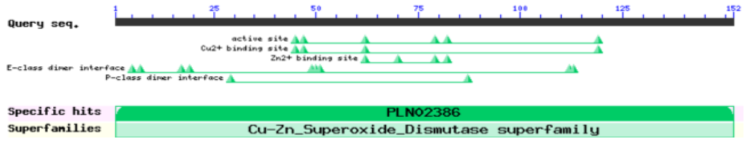

MSca9-CytCSD

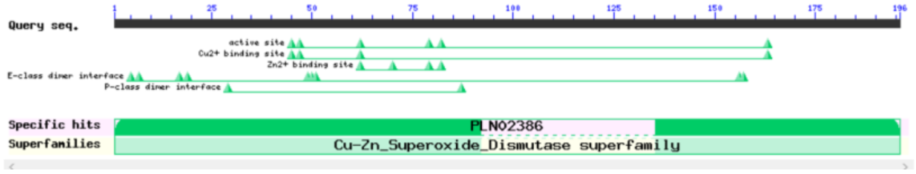

MSca13-CytCSD

ii) ***Nelumbo nucifera*:** Domain analysis of two chloroplastic CSDs (ChlCSDs) originated due to a block duplication event between chromosome MSca2 and MSca3.

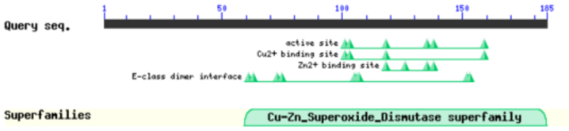

MSca2-ChlCSD

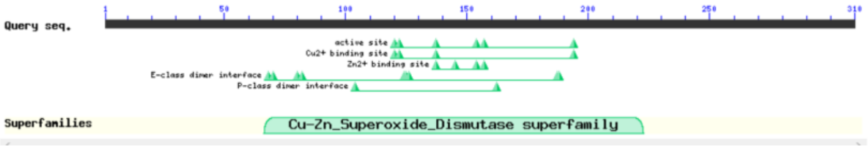

MSca3-ChlCSD

S) ***Coffea canephora*:**

i) Domain analysis of two cytosolic CSDs (CytCSDs) originated due to a block duplication between Chromosome Chr2 and Chr3.

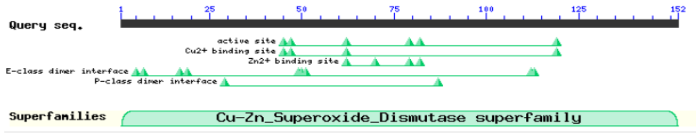

Chr2-CytCSD

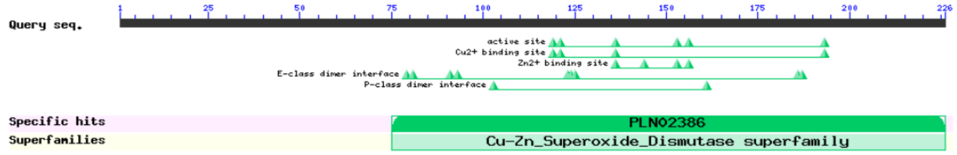

Chr3-CytCSD

ii) ***Coffea canephora*:** Domain analysis of two peroxisomal CSDs (PerCSD) originated due to a tandem duplication event (gene ids: Cc06_g23140 and Cc06_g23170) within chromosome Chr6.

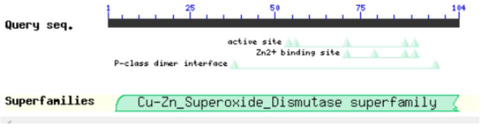

Chr6-PerCSD (Cc06_g23140)

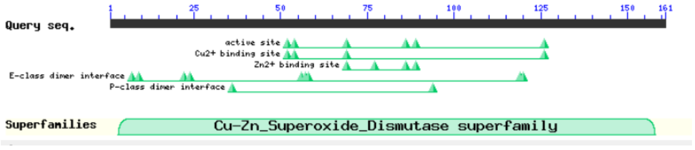

Chr6-PerCSD (Cc06_g23170)

T) ***Pyrus bretschneideri*:**

i) Domain analysis of two chloroplastic CSDs (ChlCSD) originated due to a block duplication between chromosome Sca52 and Sca89

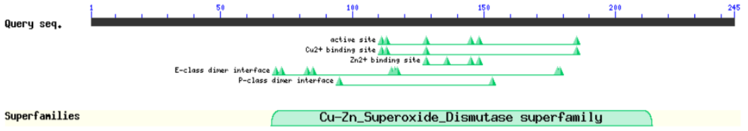

Sca52-ChlCSD

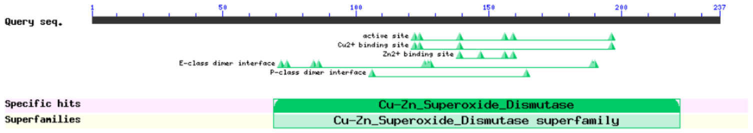

Sca89-ChlCSD

ii) ***Pyrus bretschneideri*:** Domain analysis of two peroxisomal CSDs (PerCSD) originated due to a block duplication between chromosome Sca25 and Sca143

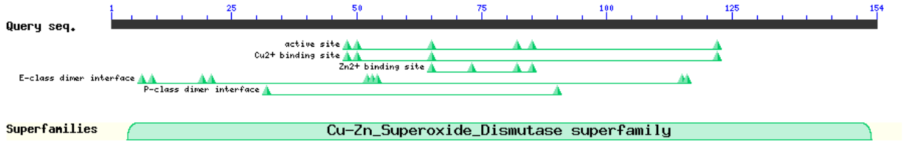

Sca25-PerCSD

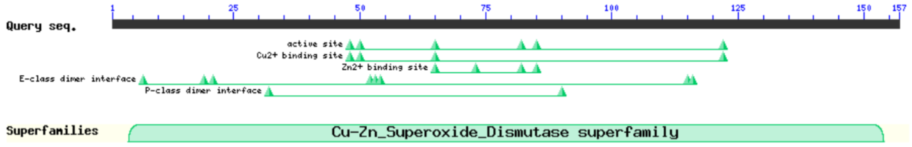

Sca143-PerCSD

U) ***Populous trichocarpa*:**

i) Domain analysis of two cytosolic CSDs (CytCSD) originated due to a block duplication event between chromosome Chr5 and Chr13

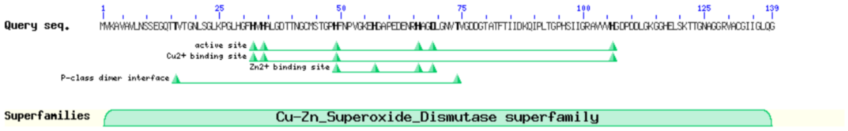

Chr5-CytCSD

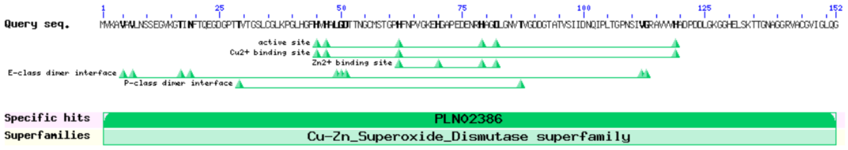

Chr13-CytCSD

ii) ***Populous trichocarpa*****:** Domain analysis of two chloroplastic CSDs (ChlCSD) originated due to a block duplication event between chromosome Chr4 and Chr9

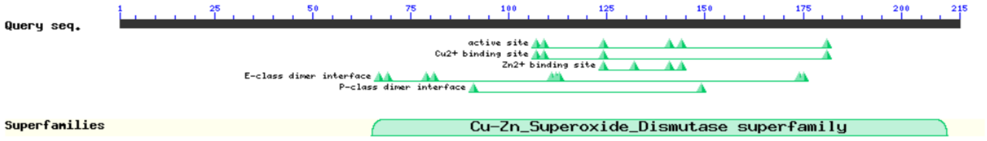

Chr4-ChlCSD

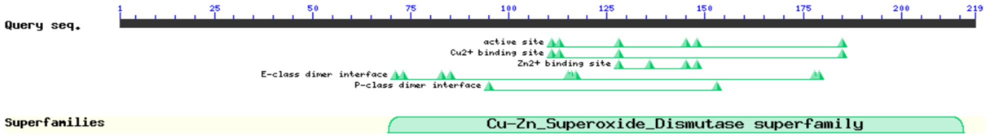

Chr9-ChlCSD

iii) ***Populous trichocarpa*****:** Domain analysis of two peroxisomal CSDs (PerCSD) originated due to a block duplication event between chromosome Chr13 and Chr19

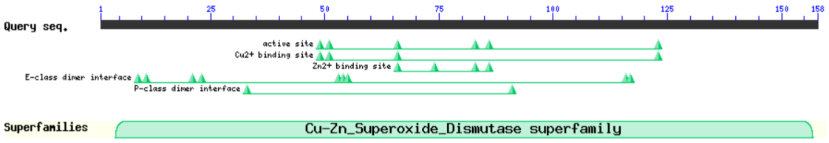

Chr13-PerCSD

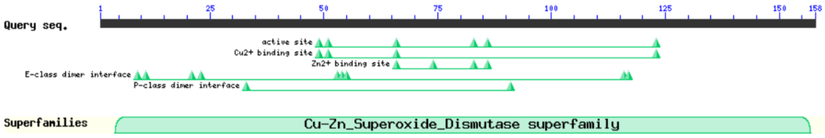

Chr12-PerCSD

Supplementary Figure 10: Schematic representation of organization of *OsCSD1* gene structure (locus designation: Os03g351300) as per Rice Annotation Project Database (https://rapdb.dna.affrc.go.jp). Scale on the top shows the relative location of exons (grey boxes), introns (dotted line), UTRs (white boxes) across the length of the gene (w.r.t. RGAP predicted *OsCSD1* gene). The position of the 'ATG' (in the is shown by a vertical arrow and the exons corresponding to the length of the coding sequence (CDS, 459 bp) is also shown below the gene. For comparison, RGAP predicted exon1, 2 and 3 (not predicted in RAP-DB) are also shown by white boxes with dotted boundary line.

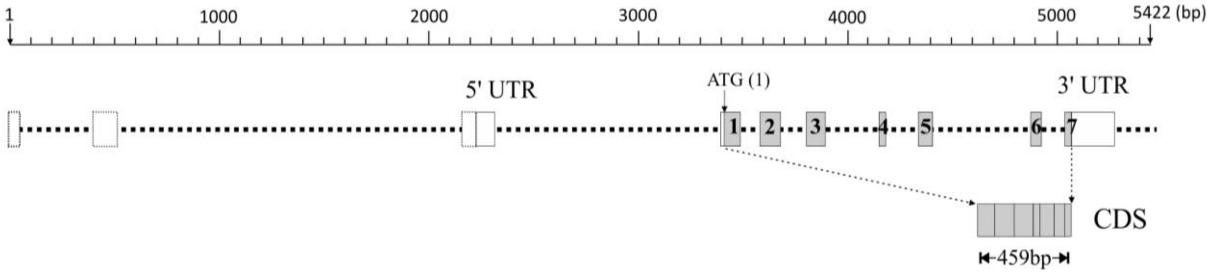

Locus Os03g351500 (*OsCSD1*)
