# Supplementary material for: Molecular and Biochemical Analysis of Duplicated Cytosolic CuZn Superoxide Dismutases of Rice and in silico Analysis in Plants
Source: Front Plant Sci. 2022 May 30;13:864330. doi: 10.3389/fpls.2022.864330 (PMC9191229; doi:10.3389/fpls.2022.864330)
Supplement: Supplementary file 2 [file Table_1.pdf]

Supplementary Table 1. Oligonucleotide primers used for PCR amplification of gene/cDNA, exon validation, and RT-qPCR analysis of rice cytosolic CuZn superoxide dismutases, *OsCSD1* and *OsCSD4*.

S. No.	Primer Name	Gene	Primer Sequence (5´-3´)	Purpose
1	CSD1-E1$_F$	*OsCSD1* (LOC_Os03g22810)	GGGAATTCCATATGGTTCAGGTCATCAGTGATG	FL-cDNA (813 bp) amplification directly (S. No. 1+2)/ joining two overlapping (S. No. 1+4 & 2+3) fragments[1]
2	CSD1$_R$		CGGAATTCTCAGCCTTGAAGTCCGATGATC	
3	CSD1-E4$_F$		ATCACATTAACAATGGTGAAGGCTG	
4	CSD1-E4a$_R$		AGTGCCCTTAACAATCTCACTGCTACC	
5	CSD1-E4a$_F$		AGCTGCGCATTCCCATATGGTGAAGGCTGTTGTTGTGCTTGG	Amplification of validated (459 bp) *OsCSD1* cDNA[2]
6	CSD4$_F$	*OsCSD4* (LOC_Os07g46990)	GGGAATTCCATATGATGGTGAAGGCTGTTGCTGTGC	FL- cDNA (459 bp) amplification
7	CSD4$_R$		CGGAATTCCTAACCCTGGAGTCCGATG	
8	CSD1-E1$_F$	*OsCSD1* (LOC_Os03g22810)	ATGGTTCAGGTCATCAGTGATGAGC	Exon-1 and exon-2 (partial) validation
9	CSD1-E2a$_R$		CTTGGCGCTAGATCCGGTGTCCA	
10	CSD1-E2$_F$		AACACGGGGCAAAGGAGGATATAT	Exon-2 region validation
11	CSD1-E2$_R$		CTTGCGCCCAAGGTCACGGACTGC	
12	CSD1-E3a$_F$		GGCCCTCAATGTTGGCTGGTG	Exon-3 region validation
13	CSD1-E3$_R$		CTCAGGCGACCCCCGCGGCGACG	
14	CSD1-E4$_F$		ATCACATTAACAATGGTGAAGGCTG	Exon-4 to exon 10 region validation[3]
15	CSD1-E3a$_F$		GGCCCTCAATGTTGGCTGGTG	Validation of transcription status of different regions of exon-3 when used in combination with CSD1$_R$ in the PCR reaction
16	CSD1-E3b$_F$		GGTGGCTGCAACTGCAAGCTGCCA	
17	CSD1-E3c$_F$		CTGCCAGTTTGACTACAAATACCACCGCA	
18	CSD1-E3d$_F$		ACCGCACACCGCTGGAGGGAGGGGAACCTTC	
19	CSD1-E3e$_F$		CCTTCCAGAAGCTCCAGATTCCAAACCAG	
20	CSD1-E3f$_F$		ACCAGCAGGAGTCGCCTCGCCTCCTC	
21	CSD1-E3g$_F$		CTCCTCCTTCATCCTCCTCGTCGTC	
22	CSD1-E3h$_F$		TCGTCGCCGCGGGGGTCGCCTGAG	
23	CSD1-qRT$_F$	*OsCSD1* (LOC_Os03g22810)	GCATGTCAACTGGGCCACACTACA	RT-qPCR analysis
24	CSD1-qRT$_R$		CATGGATATTAGCAACACCATCTTC	
25	CSD4-qRT$_F$	*OsCSD4* (LOC_Os07g46990)	CATGTCAACTGGACCACACTTCAA	RT-qPCR analysis
26	CSD4-qRT$_R$		ATTGACATTAGCAACACCATCTGC	
27	Ref1-qRT$_F$	*Actin-2* (LOC_Os10g36650)	CTAGTGGACGTACTACTGGTATTG	Reference genes in RT-qPCR analysis
28	Ref1-qRT$_R$		GATCCCTACCAGCAAGATCAAGAC	
29	Ref2-qRT$_F$	*GAPDH* (LOC_Os08g03290)	GTGACAGCAGGTCGAGCATCTTCG	
30	Ref2-qRT$_R$		GTCGATGACACGGTTGCTGTAACC	

Note: RGAP database predicted exon organization of *OsCSD1* was validated using different primer combinations (Sr. 8 to 21). Underlined sequences indicate the restriction sites (NdeI: ForP; EcoRI: RevP) used for cloning of full-length cDNA in plasmid vectors. [1]Primer combinations used for obtaining full-length (813 bp) *OsCSD1* cDNA by amplification and joining of two overlapping fragments. [2]Primer combination (CSD1-E4a$_F$ + CSD1$_R$) used for amplification of validated *OsCSD1* CDS of 459 bp. [3]Primer combination (CSD1-E4$_F$ + CSD1$_R$) used for amplification of *OsCSD1* CDS corresponding from exon-4 to exon-10.

Supplementary Table 2: List of CuZn superoxide dismutase sequences of monocot and dicot plant species retrieved from the PLAZA and GenBank databases.

Species	Gene IDs of sequences of different CuZn SOD isoforms of monocot and dicot plants retrieved from PLAZA web server			GenBank Accession Number
	Cyt	Chl	Per	Cyt
Monocots				
Ananas comosus	Aco002403, Aco006325	Aco009158, Aco010239	-	
Asparagus officinalis	evm.TU.AsparagusV1_02.2051.V1, evm.TU.AsparagusV1_08.3115.V1, evm.TU.AsparagusV1_07.1493.V1, evm.TU.AsparagusV1_03.1276.V1	evm.TU.AsparagusV1_04.331.V1, evm.TU.AsparagusV1_10.1836.V1	-	
Brachypodium distachyon	Bradi1g18340	Bradi3g43070	Bradi1g69680	
Calamus simplicifolius	CALSI_Maker00022562, ALSI_Maker00003457, CALSI_Maker00003056, CALSI_Maker00030858, CALSI_Maker00019055	CALSI_Maker00040337, CALSI_Maker00042855	-	
Cenchrus americanus	Pgl_GLEAN_10022927, Pgl_GLEAN_10003861	Pgl_GLEAN_10023687	Pgl_GLEAN_10014381	
Elaeis guineensis	p5.00_sc00021_p0011, p5.00_sc00010_p0197, p5.00_sc00032_p0132, p5.00_sc00040_p0004, p5.00_sc11605_p0001	p5.00_sc00017_p0189	-	
Hordeum vulgare	HORVU5Hr1G066930, HORVU5Hr1G066230, HORVU2Hr1G021110,	HORVU7Hr1G060130	HORVU4Hr1G067390	
Lolium perenne	LP021884	LP016678	LP002462	
Musa acuminata	Ma02_g04310, Ma08_g09720, Ma07_g03790	Ma09_g20440, Ma10_g07820	Ma04_g06850,	
Oropetium thomaeum	Oropetium_20150105_01325, Oropetium_20150105_01319, Oropetium_20150105_10285	Oropetium_20150105_14462	Oropetium_20150105 _00527	
Oryza brachyantha	OB03G26970, OB07G30860	OB08G30550	OB03G18920	
Oryza sativa	LOC_Os03g22810 (OsCSD1), LOC_Os07g46990 (OsCSD4)	LOC_Os08g44770 (OsCSD2)	LOC_Os03g11960 (OsCSD3)	MW091043, MW091044
Pennisetum glaucum	-	-	-	ABP65325
Saccharum spontaneum	Sspon.01G0009830-3C, Sspon.01G0009830-2B, Sspon.01G0009830-4D, Sspon.02G0017960-2C, Sspon.02G0017960-1A	Sspon.06G0020870-3D, Sspon.06G0020870-1B,	Sspon.01G0007640-4D, Sspon.01G0007640-3C, Sspon.01G0007640-1A, Sspon.01G0007640-2B	
Setaria italica	Seita.2G422500, Seita.9G403600	Seita.6G251600	Seita.9G488200	
Sorghum bicolor	Sobic.002G407900, Sobic.001G371900	Sobic.007G166600	Sobic.001G453800	
Triticum aestivum	TraesCS2D02G123300, TraesCS2A02G121200	TraesCS7A02G292100, TraesCS7D02G290700, TraesCS7B02G197300	TraesCS4A02G065800, TraesCS4B02G243200, TraesCS4D02G242800	
Triticum turgidum	TRITD2Av1G034290, TRITD2Bv1G043230	TRITD7Av1G138010, TRITD7Bv1G110910	TRITD4Bv1G146820, TRITD4Av1G023760	
Zea mays (B73)	Zm00001d047479, Zm00001d022505	Zm00001d031908	Zm00001d028232	
Zea mays (PH207)	Zm00008a029573, Zm00008a001493, Zm00008a035329	Zm00008a003546	Zm00008a000783	

Zoysia japonica (ssp. *nagirizaki*)	Zjn_sc00003.1.g00540.1.sm.mkhc, Zjn_sc05198.1.g00010.1.sm.mk, Zjn_sc00001.1.g01520.1.sm.mk	Zjn_sc00015.1.g08080.1.sm.mkhc, Zjn_sc00078.1.g03310.1.sm.mkhc	-	
Dicots				
Actinidia chinensis	Achn185871, Achn237031	-	-	
Arabidopsis lyrata	AL1G19260	AL4G21940	AL6G29300	
Arabidopsis thaliana	AT1G08830 (AtCSD1)	AT2G28190 (AtCSD2)	AT5G18100 (AtCSD3)	
Arachis ipaensis	Araip.ZF8SH	Araip.0J9BI, Araip.N9ZCQ	-	
Avicennia marina	-	-	-	ACA50531.1
Brassica oleracea	Bo5g009310, Bo8g112320	Bo4g165150	Bo9g157460	
Brassica rapa	Brara.I05293, Brara.F00559	Brara.D01685, Brara.G01354	Brara.J01751	
Cajanus cajan	C.cajan_10734.g, C.cajan_10731.g, C.cajan_46566.g	C.cajan_21594.g	C.cajan_16150.g	
Capsella rubella	Carubv10010551m.g	Carubv10024021m.g	Carubv10002096m.g	
Capsicum annuum	CAN.G223.4	CAN.G546.49	CAN.G904.37	
Caragana jubata	-	-	-	EF530044
Citrus clementina	Ciclev10002756m.g, Ciclev10002619m.g	Ciclev10029196m.g	Ciclev10022494m.g	
Citrus limon	-	-	-	AF318938
Coffea canephora	Cc02_g32280, Cc03_g02460	Cc01_g10280	Cc06_g23170, Cc06_g23140	
Cucumis melo	MELO3C004342, MELO3C015374	MELO3C026955	MELO3C008809	
Curcuma aromatica	-	-	-	FJ5896638
Daucus carota	DCAR_030571, DCAR_003297, DCAR_002436	DCAR_015506	DCAR_017048	
Erythranthe guttata	Migut.N00127, Migut.G00220	Migut.D02293	Migut.J01882	
Eucalyptus grandis	Eucgr.H04426, Eucgr.B01760	Eucgr.B03930	Eucgr.A02107	
Glycine max	Glyma.03G242900, Glyma.19G240400	Glyma.12G081300, Glyma.11G192700, Glyma.12G178800	Glyma.16G153900	
Hevea brasiliensis	HBR2289G022, HBR0795G030, HBR0802G005	HBR2355G036	HBR2235G041	
Malus domestica	MDO.mRNA.g.4037.5, MDO.mRNA.g.6170.8, MDO.mRNA.g.6170.7	MDO.mRNA.g.1966.3	-	
Manihot esculanta	Manes.09G160400, Manes.08G125400	-	Manes.08G145300	
Medicago truncatula	Medtr7g114240,	Medtr4g057240	Medtr6g029200	
Nelumbo nucifera	NNU_06895, NNU_18839, NNU_24182	NNU_16959, NNU_23241	NNU_03014	
Petunia axillaris	Peaxi162Scf01067g00114, Peaxi162Scf00071g00745	Peaxi162Scf00983g00213	Peaxi162Scf00463g00133	
Populus trichocarpa	Potri.005G044400, Potri.013G031100	Potri.009G005100, Potri.004G216700	Potri.013G056900, Potri.019G035800	
Potentilla atrosanguinea	-	-	-	EU532614
Pyrus bretschneideri	Pbr014198.1.g, Pbr034143.1.g	Pbr040434.1.g, Pbr030368.1.g	Pbr007701.1.g, Pbr016910.1.g	
Solanum lycopersicum	Solyc01g067740.2	Solyc11g066390.1	Solyc03g062890.2	
Solanum tuberosum	PGSC0003DMG400000417	PGSC0003DMG400010660	PGSC0003DMG40002308 6	
Tarenaya hassleriana	THA.LOC104805838, THA.LOC104823740	THA.LOC104826391, THA.LOC104815734	THA.LOC104802873	
Vigna radiata (*var. radiata*)	Vradi10g05230	Vradi04g03170, Vradi02g06330	Vradi01g11610	
Vitis vinifera	GSVIVG01021959001, GSVIVG01021922001, GSVIVG01021949001	GSVIVG01031462001	GSVIVG01033413001	

Note: The gene ids listed are as per PLAZA web resource for plant genomes (https://bioinformatics.psb.ugent.be/plaza/), while for eight sequences the GenBank accession numbers are indicated. Cyt: Cytosolic, Chl: Chloroplastic, and Per: Peroxisomal.

Supplementary Table 3: Characteristics of the duplicated CuZn superoxide dismutase genes among the monocot species analyzed in this study.

S. No.	Species	CuZn SOD genes	Duplication events (Chromosomes involved, event type, and CuZn SOD isoforms affected)			[2]Heterogeneity between duplicated CSDs		[3]Impact on SOD domain and important structural-functional features in the duplicated CuZn SODs (CSDs)
		Total (Dup)	[1]Chromosomes	Event	Isoform	Length	Sequence	
1	*O. sativa*	04 (02)	Chr3, Chr7	Block	Cyt	0	18	The SOD domain and all important features present in the duplicated CSD copies.
2	*S. italica*	04 (02)	Chr2, Chr9	Block	Cyt	1	16	
3	*S. bicolor*	04 (02)	Chr1, Chr2	Block	Cyt	1	15	
4	*O. brachyantha*	04 (02)	Chr3, Chr7	Block	Cyt	53	42	Length variation at N-Ter in Chr7-CSD, Chr7-CSD lack all Zn^{+2} binding residues (and part of active site).
5	*E. guineensis*	06 (03)	Chr5, Chr14, Chr14	Block	Cyt	0-36	13-40	One of the Chr14-CSD show C-Ter truncation, deletion at N-Ter & in middle, and lack of one Cys involved in the disulfide bond.
6	*O. thomaeum*	05 (03)	Og_20141112_001,<br>Og_20141112_001,<br>Og_20141112_027	Tandem<br><br>Block	Cyt	44	4-17	Og_2014112_027 CSD (block duplication) showed N-ter and C-Ter truncations and lacked one Cys (disulfide bond) and E-class dimer interface.
7	*Z. mays* (B73)	04 (02)	Chr7, Chr9	Block	Cyt	46	64	Length variation at both N-Ter and C-Ter in Chr7-CSD.
8	*Z. mays* (PH207)	05 (03)	Chr1, Chr7, Chr9	Block	Cyt	7-39	19-44	C-Ter variation in Chr7-CSD; Chr9-CSD contain ATPase-IIIA-H domain at N-Ter, and lacked E-class dimer interface.
9	*C. americanus*	04 (02)	Chr5, Chr7	Block	Cyt	117	20	Disruption of SOD domain and C-Ter length variation in Chr5-CSD.
10	*A. comosus*	04 (02)	LG4, LG14	Block	Cyt	58	11	Length variation at C-Ter in LG14-CSD.
11	*M. acuminata*	06 (02)	Chr9, Chr10	Block	Chl	3	17	SOD domain and all important features present in CSD copies.
12	*A. officinalis*	06 (02)	V1_04, V1_10	Block	Chl	49	27	V1_10-CSD show N- & C-Ter variation and lack E-class interface.
13	*Z. japonica* (ssp. *nagirizaki*)	05 (02)	Sca00015.1, Sca00078.1	Block	Chl	40	49	Sca00078.1-CSD show N-Ter truncation and lacks all Zn^{+2} binding residues (including active site residue) and E-class dimer interface.
14	*T. aestivum*	08 (02)	Chr2A, Chr2D	Block	Cyt	11	3	All Zn^{+2} binding residues (including active site residue) and P-class interface missing in Chr2A- CSD.
		(03)	Chr7A, Chr7B, Chr7D	Block	Chl	1	5	SOD domain and important features present in CSD copies.
		(03)	Chr4A, Chr4B, Chr4D	Block	Per	0	6	E-class interface missing in all three PerCSD copies.
15	*S. spontaneum*	11 (04)	Chr1B, Chr1C, Chr1D, Chr2C	Block	Cyt	0-38	1-60	Length variation in Chr1C-CSD (middle); Chr2C-CSD lacks all Cu^{+2} binding residues and E-class dimer interface.
		(02)	Chr6B, Chr6D	Block	Chl	0	0	SOD domain and all important features present in CSD copies.
		(04)	Chr1A, Chr1B, Chr1C, Chr1D	Block	Per	7-39	1-4	Minor length variation at N-Ter, loss of E-class interface in Chr1B-CSD; Length variation & lack of all SOD features in Chr1A-CSD.

Note: PLAZA monocots 4.5 database website: https://bioinformatics.psb.ugent.be/plaza/versions/plaza_v4_5_monocots/. [1]Chromosome designations (Chr, Vi, Og, LG, Sca etc.) indicated above are as per the monocot PLAZA 4.5 database (Bel et al., 2018). [2] Length and sequence heterogeneity of the amino acid sequences of duplicated CSDs was analyzed by pairwise alignment using ClustalX (Thompson et al. 1997). [3]Analysis of domains/important features of the proteins was carried out at Conserved Domain Database (https://www.ncbi.nlm.nih.gov/Structure/cdd/, Marchler-Bauer et al., 2011) at NCBI. Dup: number of duplicated CSDs; Cyt: Cytosolic CSD, Chl: Chloroplastic CSD, Per: Peroxisomal CSD, N/C-Ter: N/C-Terminal of protein sequence.

Supplementary Table 4: Characteristics of the duplicated CuZn superoxide dismutase genes among the dicots analyzed in this study.

S. No.	Species	CuZn SOD genes	Duplication events (Chromosomes involved, event type, and CuZn SOD isoforms affected)			[2]Heterogeneity between duplicated CSDs		[3]Presence/absence of important SOD features/domains based on CDD analysis
		Total (Dup)	[1]Chromosomes	Event	Isoform	Length	Sequence	
1	*D. carota*	05 (02)	Chr1, Chr9	Block	Cyt	0	2	The SOD domain and all important features present in the duplicated CSD copies.
2	*M. esculanta*	03 (02)	Chr8, Chr9	Block	Cyt	0	16	
3	*E. guttata*	04 (02)	Sca7, Sca14	Block	Cyt	0	20	
4	*H. brasiliensis*	05 (03)	NW_018746327.1, NW_018745806.1, NW_018746407.1	Block	Cyt	0-6	2-28	Minor length variation at C-Ter in NW_018745806.1-CSD.
5	*C. cajan*	05 (02)	CcLG03	Tandem	Cyt	1-38	18-42	CcLG03-CSDs lack Cu^{+2} binding sites, E-class dimer interface, one Cys for disulfide bond, and truncated at C-Ter (compared to third CSD1 on Sca130189CSD1, duplication not predicted).
6	*E. grandis*	04 (02)	Chr2, Chr8	Block	Cyt	17	21	Length variation at C-Ter in Chr8-CSD.
7	*C. melo*	04 (02)	Sca3, Sca25	Block	Cyt	20	44	Length variation at C-Ter in Sca25-CSD.
8	*C. clementina*	04 (02)	Sca5	Tandem	Cyt	27	70	Length variation at N-Ter and lack of E-class dimer interface in Sca5-CSD.
9	*B. oleracea*	04 (02)	Chr5, Chr8	Block	Cy	46	8	Length variation at N-Ter in Chr8-CSD.
10	*A. chinensis*	03 (02)	Chr3, Chr29	Block	Cyt	79	26	Length variation at N- and C-Ter and lack of E-class dimer interface in Chr29-CSD.
11	*V. vinifera*	05 (03)	Chr14	Tandem	Cyt	4-761	1-28	Minor deletion (4 bp) in Chr14-CSD copy (G01021949001), Chr-14 CSD copy (G0102192200) lack Cu^{+2} binding sites, E-class dimer interface, and showed disrupted SOD domain due to three partial domains (Transposase_28, fibronec_FbpA, SMC_prok_B).
12	*M. domestica*	04 (02)	Bb6170	Tandem	Cyt	263	140	Compared to Bb4037-CSD (no duplication predicted), Bb6170-CSD copy (g.6170.7) lack Cu^{+2} binding sites, active site, and E-class dimer interface, while the tandem duplicate copy (g.6170.8) show substantial length and sequence heterogeneity, contain partial SOD domain (C-Ter) and non-SOD type domains (PLN03029, Frigida).
13	*V. radiata* (*var. radiata*)	04 (02)	Chr2, Chr4	Block	Chl	47	41	Chr4-CSD showed length variation in the middle and towards N-Ter, and lack Cu^{+2} binding site, active site and, both E- and P- class interaction interfaces.
14	*A. ipaensis*	03 (02)	B03, B08	Block	Chl	16	49	Multiple blocks of deletions towards N-Ter in B08-CSD.

15	*T. hassleriana*	05	(02)	NW_010961290.1, NW_010966881.1	Block	Cyt	0	9	SOD domain and all important features present in CSD copies.
			(02)	NW_010967707.1, NW_010965696.1	Block	Chl	3	28	NW_010967707.1-CSD lacked E-class dimer interface.
16	*G. max*	06	(02)	Chr3, Chr19	Block	Cyt	0	8	SOD domain and all important features present in CSD copies.
			(03)	Chr11, Chr12, Ch12	2 Block events	Chl	4-26	14-44	Chr-12 CSD (12G178800) contained deletions towards N- and C-Ter; other copy (12G081300) contained small deletion at C-Ter.
17	*B. rapa*	05	(02)	Chr6, Chr9	Block	Cyt	0	9	SOD domain and all important features present in CSD copies.
			(02)	Chr4, Chr7	Block	Chl	46	18	Chr7-CSD showed C-Ter truncation, and lacked Cu^{+2} binding sites and surface interaction residues.
18	*N. nucifera*	06	(02)	MSca9, MSca13	Block	Cyt	44	10	SOD domain in MSca9-CSD disrupted by a 44 amino acid region.
			(02)	MSca2, MSca3	Block	Chl	125	21	MSca2-CSD and MSca3-CSD showed length variation at N-Ter, middle and C-Ter, MSca3-CSD lacked E-class interaction interface.
19	*C. canephora*	05	(02)	Chr2, Chr3	Block	Cyt	74	28	Chr3-CSD showed length variation at N-Ter region.
			(02)	Chr6	Tandem	Per	57	1	One tandem Chr6-CSD (Cc06_g23140) showed C-Ter truncation and lacked all Cu^{+2} binding sites and E-class interaction interface.
20	*P. bretschneideri*	06	(02)	Sca52, Sca89	Block	Chl	8	30	Sca52-CSD showed deletion in the middle while Sca89-CSD showed truncation at C-Ter region.
			(02)	Sca25, Sca143	Block	Per	3	9	SOD domain and all important features present in CSD copies. Sca143-CSD showed 3 amino acids deletion at C-Ter region.
21	*P. trichocarpa*	06	(02)	Chr5, Chr13	Block	Cyt	13	10	Chr5-CSD showed 133 amino acids deletion towards N-Ter truncation, and lack E-class surface interaction interface.
			(02)	Chr4, Chr9	Block	Chl	4	28	SOD domain and all important features present in CSD copies, Chr4-CSD showed minor deletion towards N-Ter region.
			(02)	Chr13, Chr19	Block	Per	0	8	SOD domain and all important features present in CSD copies.

Note: PLAZA dicots 4.5 database website: https://bioinformatics.psb.ugent.be/plaza/versions/plaza_v4_5_dicots/. [1]Chromosome designations indicated above (Chr, Vi, Og, LG, Sca etc.) are as per the dicot PLAZA 4.5 web resource (Bel et al., 2018). [2] Length and sequence heterogeneity of the amino acid sequences of duplicated CSDs was analyzed by pairwise alignment using ClustalX (Thompson et al. 1997). [3]Analysis of domains/important features of the proteins was carried out at Conserved Domain Database (https://www.ncbi.nlm.nih.gov/Structure/cdd/, Marchler-Bauer et al., 2011) at NCBI. Dup: number of duplicated CSDs; Cyt: Cytosolic CSD, Chl: Chloroplastic CSD, Per: Peroxisomal CSD, N/C-Ter: N/C-Terminal of protein sequence.
